# HIF-1 and SKN-1 Coordinate the Transcriptional Response to Hydrogen Sulfide in *Caenorhabditis elegans*

**DOI:** 10.1371/journal.pone.0025476

**Published:** 2011-09-29

**Authors:** Dana L. Miller, Mark W. Budde, Mark B. Roth

**Affiliations:** 1 Department of Biochemistry, University of Washington School of Medicine, Seattle, Washington, United States of America; 2 Division of Basic Sciences, Fred Hutchinson Cancer Research Center, Seattle, Washington, United States of America; 3 University of Washington Molecular and Cellular Biology Graduate Program, Seattle, Washington, United States of America; University of Pennslyvania, United States of America

## Abstract

Hydrogen sulfide (H_2_S) has dramatic physiological effects on animals that are associated with improved survival. *C. elegans* grown in H_2_S are long-lived and thermotolerant. To identify mechanisms by which adaptation to H_2_S effects physiological functions, we have measured transcriptional responses to H_2_S exposure. Using microarray analysis we observe rapid changes in the abundance of specific mRNAs. The number and magnitude of transcriptional changes increased with the duration of H_2_S exposure. Functional annotation suggests that genes associated with protein homeostasis are upregulated upon prolonged exposure to H_2_S. Previous work has shown that the hypoxia-inducible transcription factor, HIF-1, is required for survival in H_2_S. In fact, we show that *hif-1* is required for most, if not all, early transcriptional changes in H_2_S. Moreover, our data demonstrate that SKN-1, the *C. elegans* homologue of NRF2, also contributes to H_2_S-dependent changes in transcription. We show that these results are functionally important, as *skn-1* is essential to survive exposure to H_2_S. Our results suggest a model in which HIF-1 and SKN-1 coordinate a broad transcriptional response to H_2_S that culminates in a global reorganization of protein homeostasis networks.

## Introduction

Exogenous H_2_S has dramatic effects on mammalian physiology that can improve survival in changing environmental conditions. Mice exposed to H_2_S enter into a hibernation-like state that allows them to endure periods of low metabolic rate and decreased core body temperature without apparent ill effects [1]. The H_2_S-induced state enables mice to survive exposure to otherwise lethal hypoxic conditions [2], and improves outcome in rodent models of severe blood loss [3], myocardial infarction [4], aortic occlusion [5] and hepatic ischemia/reperfusion [6].

*C. elegans* grown in H_2_S have increased thermotolerance and lifespan [7]. Increased lifespan and thermotolerance require the conserved sirtuin homologue *sir-2.1*, though mutant animals with deletions in *sir-2.1* grow normally in H_2_S. In contrast, the *hif-1* transcription factor is required to survive exposure to H_2_S [8]. *hif-1* is a highly conserved bHLH transcription factor that is well-known for its role in coordinating the transcriptional response to hypoxia in all animals, including *C. elegans* [9], [10]. Sirtuins have been shown to modulate lifespan in yeast, worms, flies and mice [11]. Recent work has demonstrated that HIF-1 activity can influence lifespan in *C. elegans* [12], [13], [14]. Thus, the response to H_2_S involves at least two genes, *hif-1* and *sir-2.1*, which influence lifespan.

In this study, we investigated the transcriptional response to H_2_S in *C. elegans*. Using an unbiased microarray approach, we show that there are rapid and progressive changes in mRNA abundance associated with exposure to H_2_S. Functional genomic analysis suggests that adaptation to H_2_S results in significant changes to protein homeostasis pathways. We found that *hif-1* is required for nearly all of the early changes observed, though there is little overlap between genes regulated in response to H_2_S and those that have been reported to change in hypoxia. Moreover, our data show that other factors contribute to coordinate the response to H_2_S, as we found that some H_2_S-induced transcriptional changes require the *skn-1* transcription factor. We demonstrate that, like *hif-1*, *skn-1* is required to survive in low concentrations of H_2_S. These data suggest a possible model in which HIF-1 and SKN-1 act together to coordinate a transcriptional response to H_2_S that ultimately leads to alterations in the expression of genes involved in protein homeostasis.

## Results

### H_2_S exposure leads to rapid and progressive changes in mRNA abundance

**Figure 1:**
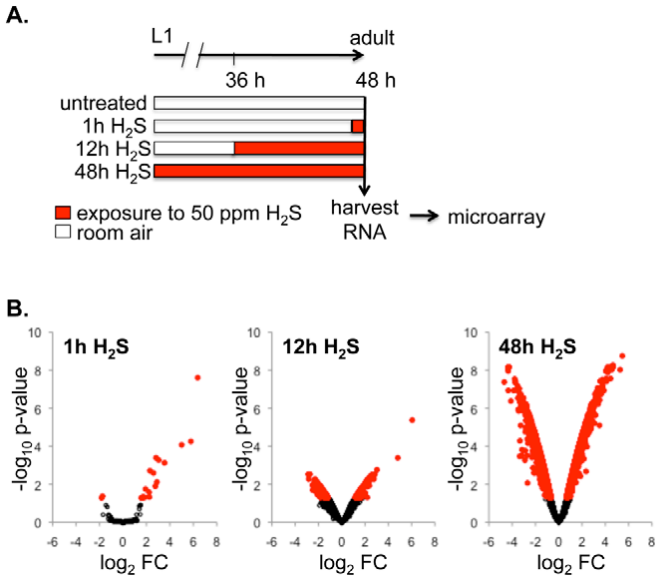
Exposure to H_2_S induces rapid and progressive changes in mRNA abundance. A. Experimental design schematic. *C. elegans* were grown from synchronized first-stage larvae (L1) for 48 h to young adult before being collected for RNA extraction. Each bar represents 48 h from L1 to first-day adult for one experimental group. Time in room air is indicated in white and time in H_2_S indicated in red. Exposure to H_2_S (50 ppm in room air) was always immediately prior to isolating RNA. B. Changes in mRNA abundance measured by microarray. Plots show magnitude of change in transcript level (log_2_ FC) as a function of adjusted p-value (log_10_ p-value). Each point is data from one gene product. Significant changes (adj. p-value<0.05) are red. After 1 h exposure to H_2_S (left), 16 genes were significantly up-regulated and one was down-regulated (Table 1). After 12 h exposure to H_2_S (middle), 445 transcripts were significantly changed, with 259 up-regulated (Tables 1 and S1). After 48 h in H_2_S (right), 5089 transcripts were significantly altered relative to untreated controls (Table S2).

To investigate the transcriptional responses to H_2_S in *C. elegans*, we employed a microarray approach to identify mRNAs that were altered in abundance by exposure to H_2_S. In these experiments, we exposed synchronized cultures of *C. elegans* to 50 ppm H_2_S for 1, 12 or 48 h (schematized in Fig. 1A). We measured the response to 50 ppm H_2_S, as this is the same concentration of H_2_S that increases lifespan and thermotolerance [7]. Exposure to H_2_S was immediately prior to harvest. We previously showed that developmental rate is not affected by this concentration of H_2_S [7], ensuring that all animals were first-day adults when RNA was harvested. This experimental design enabled us to compare transcript abundance without confounding effects from comparing different developmental stages.

The number of transcriptional changes we observed in animals exposed to H_2_S increased with the duration of exposure (Figure 1B). After 1 h exposure we detected significantly altered mRNA levels of 17 transcripts (adjusted p-value<0.05, see Experimental Procedures). All but one of these transcripts was more abundant in the animals exposed to H_2_S compared to untreated controls (Table 1), suggesting that the transcription of these genes was increased upon exposure to H_2_S. The effect of H_2_S on transcript abundance progressed with increased duration of exposure to H_2_S, both in magnitude of effect on mRNA level and number of gene products affected. After 12 h exposure to H_2_S, we observed 445 mRNA that were significantly changed, 259 (58%) of which were more abundant after exposure to H_2_S (Table S1). Nine of the 16 gene products that were increased after 1 h exposure to H_2_S were still significantly increased after 12 h in H_2_S. The scope and magnitude of transcript alterations was even more pronounced after 48 h in H_2_S, the time required to observe an increase in lifespan and thermotolerance [7]. We observed 5,089 genes that had significantly altered mRNA levels after 48 h exposure to H_2_S (Table S2), which represents 44% of the gene products included in the analysis. Of the significantly altered transcripts, 143 were increased by at least 5.5-fold and 126 were decreased by at least 5.5-fold. Together, these data indicate that there is a rapid and progressive induction of transcriptional activity upon exposure to H_2_S.

**Table 1:** Changes in mRNA abundance associated with exposure to H_2_S.

Significant changes after 1 h exposure to 50 ppm H_2_S			
Probe	gene name; description	fold-change	adj. p a
Y38E10A.25	*nspe-6*; nematode specific peptide family, group E	84.4	2.5×10^−8^
Y38E10A.12	*nspe-3*; nematode specific peptide family, group E	55.7	5.6×10^−5^
F37B1.8	*gst-19*; glutathione S-transferase	32	8.5×10^−5^
ZK1058.6	*nit-1*; carbon-nitrogen hyodrolase	7	4.1×10^−4^
T05B4.1	*lgc-1*; predicted ligand-gated ion channel	8	5.3×10^−4^
Y38E10A.15	*nspe-7*; nematode specific peptide, group E	11.3	7.4×10^−4^
R08E5.1	not annotated	4.9	1.9×10^−3^
Y38E10A.26	*nspe-2*; nematode specific peptide family, group E	6.1	2.5×10^−3^
F02H6.5	*sqrd-1*; sulfide∶quinone oxidoreductase	7.5	7.6×10^−3^
K10H10.3a	*dhs-8*; dehydrogenase with different specificities	7	0.013
K10H10.2	*cysl-2*; cysteine synthase related	3.7	0.018
T05B4.2	*nhr-57*; hormone receptors	4.6	0.026
C18H7.1	von Willebrand factor and related coagulation proteins	−3.2	0.043
C31C9.2	D-3-phosphoglycerate dehydrogenase	3.2	0.046
W07A12.7	*rhy-1*; regulator of *hif-1*, predicted acyltransferase	4.6	0.047
Y38E10A.16	*nspe-5*; nematode specific peptide family, group E	3.2	0.049
Y38E10A.11	*nspe-4*; nematode specific peptide family, group E	3.5	0.049

Most increased transcripts in abundance after 12 h exposure to 50 ppm H_2_S			
Probe	gene name; description	fold-change	adj. p a
F37B1.8	*gst-19*; glutathione S-transferase	66	4.2×10^−6^
R08F11.4	SAM-dependent methyltransferases	28	4.1×10^−4^
F02H6.5	*sqrd-1*; sulfide∶quinone oxidoreductase	8.2	1.7×10^−3^
T24B8.5	ShK-like toxin peptide containing a domain rich in cysteine	7.2	3.0×10^−3^
Y41C4A.17	not annotated	6.8	5.3×10^−3^
F58E6.7	not annotated	6.6	5.3×10^−3^
F15B9.1	not annotated	6.5	5.3×10^−3^
K11G9.6	*mtl-1*; predicted metallothionein, cysteine-rich	6.3	5.3×10^−3^
ZK899.6	not annotated	6.2	3.7×10^−3^
R08E5.1	not annotated	6.1	3.1×10^−3^
Y110A2AL.3	not annotated	5.5	3.6×10^−3^
F46C5.1	not annotated	5.2	1.2×10^−2^
C52D10.7	*skr-9*; SCF ubiquitin ligase, Skp1 component	5.0	7.4×10^−3^
F18E3.2	*srw-67*; 7-transmembrane olfactory receptor	4.8	0.01
C03E10.6	*clec-222*; C-type lectin	4.7	5.3×10^−3^
M162.5.1	Permease of the major facilitator superfamily	4.7	5.3×10^−3^
C45G7.2	*ilys-2*; invertebrate lysozyme	4.6	0.049
ZC412.6	*nspa-5*; nematode specific peptide, class A	4.6	0.013
Y22D7AR.10	Histone acetyltransferase (MYST family)	4.5	0.01
H12D21.1	*nspa-1*; nematode specific peptide, class A	4.5	6.0×10^−3^
R09E12.9	not annotated	4.2	7.4×10^−3^
H12D21.13	*nspa-4*; nematode specific peptide, class A	4.1	0.011
H12D21.12	*nspa-2*; nematode specific peptide, class A	4.1	8.2×10^−3^
F01D5.3	Secreted surface protein	4.0	0.029
C54F6.5	not annotated	4.0	0.011
C34E11.4	not annotated	4.0	0.011
H12D21.14	*nspa-6*; nematode specific peptide, class A	4.0	0.011
Y50E8A.17	*nspa-10*; nematode specific peptide, class A	4.0	0.013
Y27F2A.8	not annotated	4.0	0.011
K10G6.5	not annotated	3.9	0.01

a adjusted p-value, corrected for multiple testing and false discovery rate.

We used quantitative reverse transcript PCR (qRT-PCR) to validate changes in mRNA levels that we observed by microarray. We focused on transcripts that changed after brief exposure to H_2_S, reasoning that these early transcriptional changes represent the initial response to H_2_S and may be important to set up later, more progressive changes. When wild-type animals were grown on *E. coli* strain OP50, 6 of the 11 gene products that were predicted to be increased based on the microarray data were more abundant after 1 h exposure to H_2_S (Table 2). Several of the gene products trended toward higher expression, but did not reach significance in this assay. For many of these, we noticed that the level of transcript measured in the untreated sample was near background, which may have increased the variance in the measurements. These changes did reach significance when animals were grown on HT115(DE3) RNAi control food. In these conditions, 10 of the 11 gene products tested were upregulated after 1 h exposure to H_2_S (Table 2). In general, the magnitude of H_2_S-induced changes in transcript abundance was greater on RNAi food than on OP50. The source of this variation is unclear, but may hint at an effect of nutritional status on adaptation to H_2_S. Indeed, the HT115(DE3) bacterial food used for feeding RNAi has previously been shown to affect survival in hypoxia (DLM and MBR, unpublished observation and [15]), consistent with the idea that nutritional status can influence responses to environmental changes. The one gene product that was less abundant after exposure to H_2_S by microarray, C18H7.1, was not significantly altered after exposure to H_2_S in either nutrient condition in qRT-PCR measurements. Further validating these results, our microarray data corroborate previous studies that showed T05B4.2 (*nhr-57*) and K10H10.2 (*cysl-2*) are upregulated after short exposure to H_2_S [8]. We conclude that our microarray experiments identified transcripts that change in mRNA abundance associated with exposure to H_2_S.

**Table 2:** qRT-PCR Validation of changes in transcript abundance after 1 h H_2_S.

fold-change:	microarray^a^	OP50^b^	HT115
Y38E10A.12	56	73.0±2.8*	144.8±2.4*
F37B1.8	32	7.4±1.6*	153.9±1.7*
ZK1058.6	7	11.2±2.4*	18.4±1.2*
T05B4.1	8	6.9±3.0	16.2±1.7*
R08E5.1	4.9	2.1±2.6	7.4±1.4
F02H6.5	7.5	3.0±1.9	12.9±1.2*
K10H10.3a	7	1.8±1.3	10.2±1.2*
K10H10.2	3.7	18.9±7.5*	20.6±1.6*
T05B4.2	4.6	5.0±3.7	7.4±2.1*
C18H7.1	−3.2	−1.7±2.0	1.4±1.5
C31C9.2	3.2	2.2±1.2*	9.4±2.7*
W07A12.7	4.6	3.1±1.9*	10.4±1.2*

a Fold-change of transcript as measured by microarray, as in Table 1.

b Fold-change of transcript as measured by qRT-PCR. Animals were grown on E. coli OP50 strain or the HT115(DE3) strain containing the control RNAi plasmid L440.

*p<0.05.

### Function annotation of genes induced by exposure to H_2_S

Only 16 gene products were more abundant after exposure to H_2_S for 1 h, which precluded the use of bioinformatic analysis to measure enrichment of functional classes. However, we noticed that many of the genes on this list are annotated to be involved in cellular metabolic processes (Table 1). The most-highly expressed gene in response to H_2_S is a glutathione-S transferase, F37B1.8 (*gst-19*). Two other up-regulated genes are predicted to have a role in amino acid metabolism, including the rate-limiting enzyme in serine production, phosphoglycerate dehydrogenase (C31C9.2), and an enzyme with homology to cysteine synthase (*cysl-2*, K10H10.2). We also observed upregulation of *nit-1* (ZK1058.6), a predicted carbon-nitrogen hydrolase. In addition to metabolic enzymes, exposure to H_2_S also resulted in the upregulation of 6 of the 8 *nspe* (nematode specific peptide, class E) genes. There is little known about these genes, other than they code for short peptides, 70–75 amino acids long, that are annotated to be integral to the membrane. Although the *nspe* transcripts were greatly increased in abundance after 1 h exposure to H_2_S, they were not significantly changed after 12 h exposure to H_2_S, suggesting that they were only transiently upregulated. Instead, after 12 h in H_2_S we found that 8 of the 12 *nspa* (nematode specific peptide, class A) transcripts were significantly upregulated (Table S1).

To evaluate functional categories of genes over-represented in the microarray dataset of transcripts changed after longer exposure to H_2_S, we employed the online Database for Annotation, Visualization and Integrated Discovery (DAVID, v6.7) functional annotation clustering tool [16], [17]. We focused on gene products that were most increased in response to H_2_S, as we hypothesize that these might be important for the phenotypic changes that we observe and likely to be most robust. This analysis showed that there were two highly enriched functional clusters in the genes increased by at least 3-fold after 12 h exposure to H_2_S (Table 3, Table S3). The most enriched cluster included gene ontology terms related to aging and stress resistance. This result is consistent with our previous observation that adaptation to H_2_S increases lifespan and thermotolerance [7].

**Table 3:** Functional annotation of gene products increased after exposure to H_2_S.

12 h exposure to H_2_S^a^			
Aging and Stress Response cluster; enrichment score = 2.4			
Term	count	fold enrichment	p
GO:0009408: response to heat	3	40	0.0022
GO:0007568: aging	5	7.7	0.0029
GO:0008340: determination of adult life span	5	7.7	0.0029
GO:0010259: multicellular organismal aging	5	7.7	0.0029
GO:0009266: response to temperature stimulus	3	25	0.0055
GO:0009628: response to abiotic stimulus	3	13	0.021

F-box associated cluster; enrichment score = 1.2			
Term	count	fold enrichment	p
SM00256: FBOX	5	3.4	0.048
IPR001810: Cyclin-like F-box	5	3.4	0.057
IPR012885: F-box associated type 2	3	3.3	0.12

48 h exposure to H_2_S^b^			
F-box associated cluster; enrichment score = 20.3			
Term	count	fold enrichment	p
IPR012885: F-box associated type 2	25	27	1×10^28^
IPR001810: Cyclin-like F-box	25	11	2×10^19^
SM00256: FBOX	25	6.7	5×10^15^

BTB/POZ associated cluster; enrichment score = 1.1			
Term	count	fold enrichment	p
IPR011333: BTB/POZ fold	5	6.0	0.009
IPR013069: BTB/POZ	3	3.2	0.09
IPR000210: BTB/POZ-like	3	3.2	0.18
SM00225: BTB	3	3.2	0.37

transcription associated cluster; enrichment score = 0.8			
Term	count	fold enrichment	p
GO:0030528: transcription regulator activity	6	3.3	0.02
GO:0045449: regulation of transcription	6	2.5	0.07
GO:0006355: regulation of transcription, DNA-dependent	5	2.5	0.1
GO:0051252: regulation of RNA metabolic process	5	2.5	0.1
GO:0003700: transcription factor activity	4	2.7	0.2
GO:0003677: DNA binding	4	1.7	0.4
SP_PIR_KEYWORD: dna-binding	3	1.1	0.8
SP_PIR_KEYWORD: nucleus	4	0.9	0.8

metal binding cluster; enrichment score = 0.8			
Term	count	fold enrichment	p
IPR000571:Zinc finger, CCCH-type	3	16	0.01
SM00356:ZnF_C3H1	3	9.5	0.04
GO:0046914∼transition metal ion binding	7	1.6	0.19
GO:0008270∼zinc ion binding	6	1.8	0.19
GO:0046872∼metal ion binding	7	1.4	0.35
GO:0043169∼cation binding	7	1.3	0.38
GO:0043167∼ion binding	7	1.3	0.38
SP_PIR_KEYWORD: zinc	5	1.3	0.51
SP_PIR_KEYWORD: metal-binding	6	1.3	0.51

a 12 h gene list included 91 genes whose mRNA was significantly increased after 12 h exposure to H_2_S (greater than 3-fold change in mRNA abundance, corrected p<0.05).

b 48 h gene list included 95 genes whose mRNA was significantly increased after 48 h exposure to H_2_S (greater than 5.6-fold change in mRNA abundance, corrected p<1×10^−5^).

We observed that proteins with the F-box motif were enriched in the genes upregulated by H_2_S. F-box proteins were first identified as members of the SCF (Skp-cullin-F-box) ubiquitin ligase complex that targets proteins for ubiquitination and eventual degradation [18], [19]. F-box proteins were significantly enriched in genes upregulated after 12 h exposure to H_2_S (Table 3). These data raise the possibility that the response to H_2_S alters the stability of proteins regulated by the ubiquitin-proteosome system. When we extended this analysis to define the functional clusters in gene products upregulated by at least 5-fold after 48 h exposure to H_2_S, we observed an even greater enrichment of F-box containing proteins (Table 3). Moreover, the second most enriched cluster included genes associated with the BTB/POZ domain, another protein-protein interaction motif that has been associated with SCF function [20]. We did not observe an enrichment of genes involved in aging or stress resistance after 48 h exposure to H_2_S. Together, these data show that the transcriptional response to H_2_S includes many gene products involved in protein turnover mediated by the ubiquitin proteosome system.

### The hif-1 transcription factor is required for H_2_S-induced transcriptional changes

HIF-1 is the *C. elegans* homologue of the highly conserved hypoxia inducible transcription factor, best known for its role in coordinating the transcriptional response to decreased O_2_ [9], [10], [21]. Recently, it has been demonstrated that *C. elegans* require *hif-1* to survive exposure to H_2_S [8]. On exposure to H_2_S, the HIF-1 protein accumulates and is localized to the nucleus. At least some transcriptional responses to H_2_S result from the activation of HIF-1, including K10H10.2 (*cysl-2*), *nhr-57*, and *sqrd-1* [8], [22]. These observations motivated us to consider the possibility that our microarray data might reveal other *hif-1*-dependent transcriptional responses to H_2_S.

**Figure 2:**
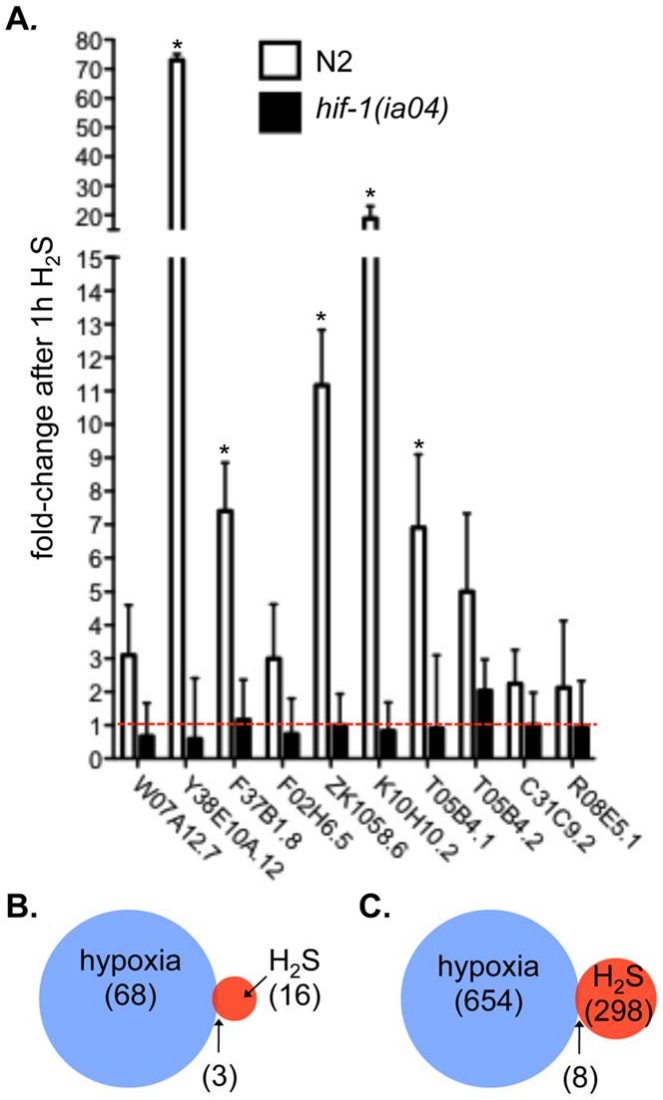
HIF-1 is required for early transcriptional responses to H_2_S. A. H_2_S-induced transcriptional changes require HIF-1. Changes in mRNA abundance after 1 h exposure to H_2_S were measured by qRT-PCR in wild-type (N2, open bars) and *hif-1(ia04)* mutant animals (filled bars). Three biological replicates for each group were performed, and each PCR reaction was run in duplicate. Error bars represent the standard deviation of the biological replicates, as propagated through the ΔΔC_t_ and fold-change calculations. *Difference between induction in wild-type (N2) is statistically different than in *hif-1(ia04)* mutant animals, p<0.05. Red dashed line demarks where transcript levels in H_2_S are the same as in room air. B. Transcriptional changes after 1 h exposure to H_2_S overlap slightly with *hif-1*-dependent changes in response to hypoxia. 3 of 16 transcripts upregulated in response to 1 h exposure to H_2_S were identified as *hif-1*-dependent targets in hypoxia (n = 68) [15]. The probability of observing this overlap randomly is 0.001. C. There is minimal overlap between the transcriptional responses to hydrogen sulfide and hypoxia. Venn diagram shows overlap between genes induced by exposure to 12 h H_2_S (n = 298) and all genes products that are altered by hypoxia (n = 654) [15]. The probability of randomly observing an overlap of 8 genes between these datasets is 0.006.

We measured the abundance of mRNA in *hif-1(ia04)* mutant animals exposed to H_2_S for 1 h by qRT-PCR to evaluate if *hif-1* is required for transcriptional changes that occur upon exposure to H_2_S. This short exposure to H_2_S was sufficient to induce transcriptional changes in wild-type, but was not lethal to the *hif-1(ia04)* mutant animals. In fact, after 1 h in H_2_S the *hif-1(ia04)* mutant animals were still moving normally [8]. We found that for 5 of 11 mRNAs tested, transcript levels in *hif-1(ia04)* mutant animals were significantly lower than wild-type (Figure 2). This includes the most highly-induced mRNAs. In the *hif-1(ia04)* mutant animals we observed very little change in any transcript abundance after exposure to H_2_S, both for messages that were highly induced as well as the lower-expressed transcripts where statistical significance of p<0.05 was not achieved. We conclude that *hif-1* has a centrally important function in inducing transcriptional changes associated with exposure to H_2_S, particularly those changes that occur immediately upon exposure to H_2_S.

Although the response to hypoxia and H_2_S both require the *hif-1* transcription factor, there is little overlap between genes regulated in these two conditions. A previous report identified 68 hypoxia-induced, HIF-1-dependent transcriptional changes in *C. elegans* [15]. Of the 16 genes upregulated after 1 h exposure to H_2_S, only 3 (19%) are also regulated by *hif-1* in hypoxia: *nhr-57*, *rhy-1* and K10H10.2 (*cysl-2*) (Figure 2B, Table S4). The slight overlap between these data sets is greater than would be expected by chance (hypergeometric probability 0.001), consistent with our observation that that *hif-1* is required for both responses. Similarly, of the 298 transcripts that are more abundant after 12 h exposure to H_2_S, 8 (3%) are also regulated by hypoxia (hypergeometric probability 0.006; Figure 2C, Table S4). Since *hif-1(ia04)* mutant animals die after 12 h exposure to H_2_S we could not determine which of these changes were *hif-1*-dependent. Thus, we included all hypoxia-induced genes, regardless of whether they require *hif-1*. These results show that there is statistically significant, though rather minimal similarity between transcriptional responses to H_2_S and hypoxia. Although our microarrays were not performed under exactly the same conditions as the previous hypoxia studies, these results suggest the interesting possibility that HIF-1 activates different spectrum of targets depending on whether it is activated by hypoxia or H_2_S. Consistent with this view, HIF-1 activity, as measured by an *nhr-57::GFP* transcriptional reporter, is in different tissues of animals exposed to hypoxia as compared to H_2_S [8].

### Role of SKN-1 in response to H_2_S

We noted many Sdz genes were upregulated after 12 or 48 h exposure to H_2_S, including several with F-box domains (Tables S1 and S2). Sdz genes were named for their *skn-1* dependent zygotic expression during embryogenesis between the 4- and 12- cell stage [23]. Several Sdz transcripts that were more abundant after exposure to H_2_S contained F-box domains (Table S3). *skn-1* is a maternally-supplied factor required early in embryogenesis for specification of the EMS blastomere that gives rise to mesendodermal lineages [24], and acts postembryonically in the intestine to control the phase II response to oxidative stress [25] and in the two ASI neurons to control the effects of dietary restriction on lifespan [26]. The abundance of Sdz genes on the list led us to consider the possibility that *skn-1* is also involved in the response to H_2_S.

**Figure 3:**
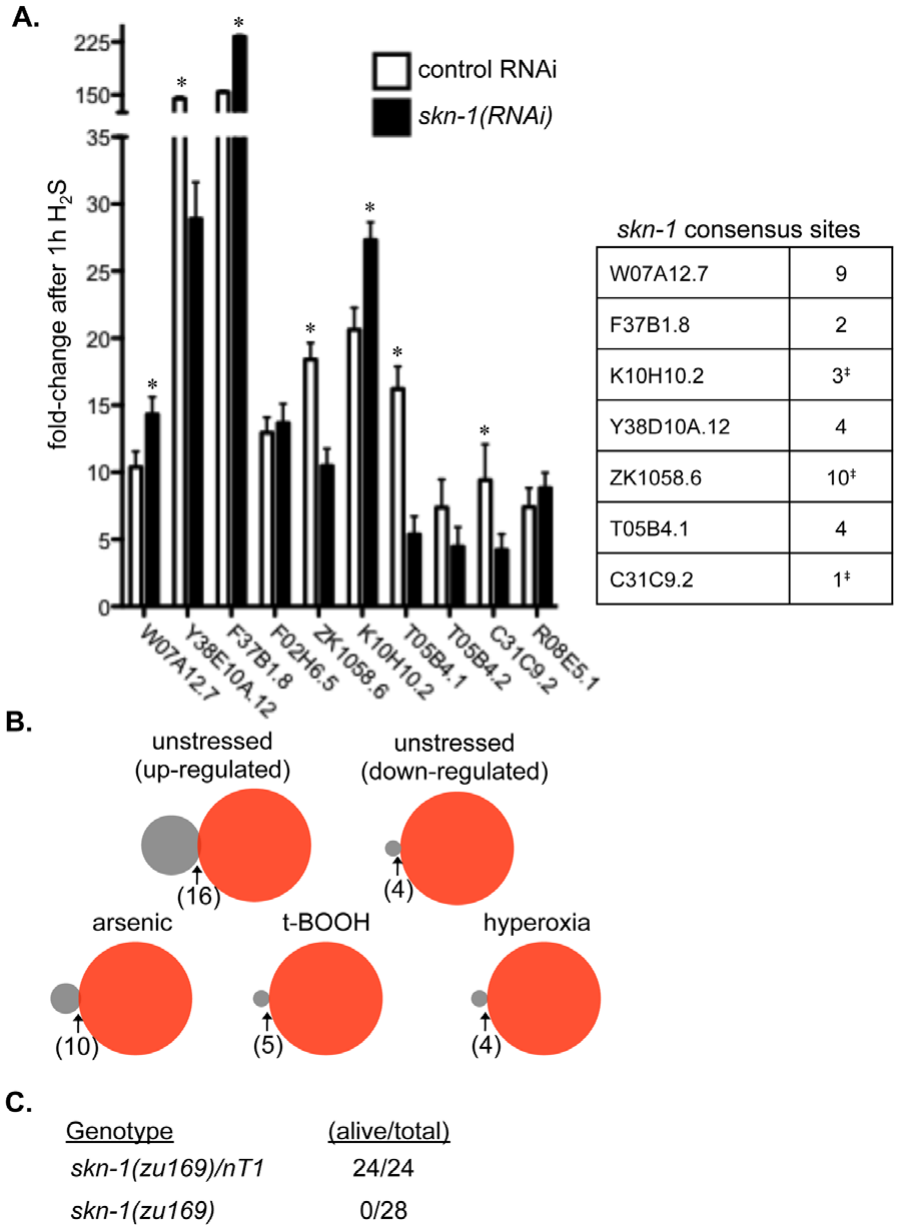
SKN-1 is essential for appropriate response to H_2_S. A. Some H_2_S-induced transcriptional changes require *skn-1*. Changes in mRNA abundance after 1 h exposure to H_2_S were measured by qRT-PCR in N2 animals grown on control RNAi food L4440 (open bars) or on *skn-1(RNAi)* (filled bars). Three biological replicates for each group were performed, and each PCR reaction was run in duplicate. Error bars represent the standard deviation of the biological replicates, propagated through the ΔΔC_t_ and fold-change calculations. *Difference between induction in control is significantly different than *skn-1(RNAi)* p<0.05. Table shows the frequency that core *skn-1* consensus sites (RTACT, [27]) are found within the upstream 2 kb flanking region of each transcript whose regulation in response to H_2_S was altered by *skn-1(RNAi)*. ^‡^genes reported to have SKN-1 bound in the promoter in the ModENCODE database [38]. B. There is little similarity between response to H_2_S and other *skn-1*-dependent transcriptional responses. The overlap between the H_2_S-regulated genes after 12 h (n = 445) was greater than chance when compared with *skn-1*-dependent gene products in unstressed conditions (n = 233, 16 common transcripts, hypergeometric probability 0.006) and for genes that require *skn-1* for arsenic-induced upregulation (n = 118, 10 common transcripts, hypergeometric probability 0.01) [27]. There was not significant overlap between transcripts altered by exposure to H_2_S and *skn-1* dependent transcripts that are downregulated in unstressed conditions (n = 63, hypergeometric probability 0.13), upregulated by tert-butyl hydroperoxide (n = 64, hypergeometric probability 0.06) or hyperoxia (n = 68, hypergeometric probability 0.15). C. *skn-1* is required to survive exposure to H_2_S. Unc animals (*skn-1/nT1* heterozygotes) were compared to non-Unc, *skn-1* homozygotes for sensitivity to H_2_S (#animals alive/total after exposure to 50 ppm H_2_S).

To test the possibility that H_2_S-induced transcriptional changes depended on *skn-1*, we measured mRNA abundance by qRT-PCR from N2 wild-type animals raised on *skn-1(RNAi)* and exposed to H_2_S for 1 h as adults. These animals laid only dead eggs, demonstrating that SKN-1 levels were reduced below those required for early embryonic development. We found that 7 of the gene products showed *skn-1*-dependent changes in abundance after 1 h exposure to H_2_S (Figure 3). Three genes regulated in a *skn-1*-dependent manner in response to H_2_S, C31C9.2, K10H10.2 and ZK1058.6 (*nit-1*), were previously shown to be regulated by *skn-1* postembryonically [27], as was embryonic expression of ZK1058.6 in the EMS lineage after the 4-cell stage and in E descendants after the 300-cell stage [23]. In addition to previously-identified *skn-1* dependent transcripts, H_2_S-induced upregulation of Y38D10A.12 and T05B4.1 also required *skn-1*. The promoter region of all these genes contain core *skn-1* consensus binding sequences, RTACT [27] (Figure 3A). We further observed that three gene products up-regulated by 1 h exposure to H_2_S were changed even more dramatically in the *skn-1(RNAi)* animals: K01H10.2, F37B1.8 (*gst-19*) and W07A12.7 (*rhy-1*). These data are consistent with reports that *skn-1* can act to negatively regulate the expression of genes involved in the response to some oxidative stresses [27]. Our data show that *skn-1* acts to both up and down regulate genes in response to H_2_S.

There is minimal overlap between transcriptional responses to H_2_S and previously characterized, post-embryonic *skn-1* dependent transcripts (Figure 3B, Table S5). We observed significant overlap between the set of genes changed in response to H_2_S and transcripts that require *skn-1* for normal expression in unstressed conditions (16 genes in common, hypergeometric probability 0.006) [27], though 4 of these 16 genes were less abundant after exposure to H_2_S. Similarly, 10 H_2_S-regulated genes were identified as *skn-1* depenendent targets in response to arsenic stress (hypergeometric probability 0.01) [27]. In contrast, we did not observe significant overlap with *skn-1*-dependent targets induced by exposure to t-butyl hydroperoxide (hypergeometric probability 0.06) [27], those downregulated in unstressed conditions (hypergeometric probability 0.13) [27], or messages regulated in response to hyperoxia (hypergeometric probability 0.15) [28]. Our observations suggest that *skn-1*-dependent transcriptional responses to H_2_S are somewhat distinct from *skn-1* mediated responses to oxidative and xenobiotic stress. This is consistent with accumulating evidence that *skn-1* transcriptional outputs are context dependent [27].

We monitored the viability of *skn-1* mutant animals exposed to H_2_S to evaluate the functional significance of the *skn-1-*dependent transcriptional response (Figure 3C). Like wild-type animals, all *skn-1(zu169)/nT1* control animals survived exposure to 50 ppm H_2_S (n = 24, 2 independent experiments). In contrast, none of the *skn-1(zu169)* homozygous mutant animals tested survived (n = 28 in same 2 experiments). To rule out the possibility that H_2_S-induced death was a result of the *nT1* balancer chromosome, we crossed the balancer away from the *zu169* allele. 24% (16/68) of the self-progeny from *zu169*/+ heterozygotes died when exposed to 50 ppm H_2_S. This is not statistically different the expected frequency of *skn-1(zu169)* homozygotes (25%; χ^2^ = 0.078, df = 1, p>0.05). Of the survivors, 14/15 were fertile, indicating that these animals were not homozygous for the *skn-1* allele, which confers a maternal-effect lethal phenotype. We conclude that the *skn-1(zu169)* homozygous animals died when exposed to H_2_S. These data demonstrate that SKN-1 activity is essential to appropriately respond to H_2_S.

## Discussion

Our results indicate that *hif-1* and *skn-1* cooperate to orchestrate a progressive transcriptional response to H_2_S. Previous studies have demonstrated *hif-1* dependent responses to H_2_S [8], [22]. We have extended this observation using an unbiased microarray approach that identified several new *hif-1*-dependent transcriptional changes upon exposure to H_2_S. In addition, we have identified *skn-1* as another essential factor during exposure to H_2_S.

H_2_S protects mice from otherwise lethal whole-body hypoxia [2] and improves outcome in a variety of rodent models of ischemia/reperfusion injury [4], [29], [30]. The mammalian orthologue of HIF-1 has been implicated in protection against ischemia/reperfusion in mammals [31]. Thus, the observation that HIF-1 is activated by H_2_S suggests a mechanistic basis for the beneficial effects of H_2_S [8]. Curiously, our data suggest that there is little overlap between transcriptional targets of HIF-1 in hypoxia and H_2_S. These results may indicate that H_2_S does not mediate protection against ischemia simply by inducing a standard hypoxia response. Further understanding this conserved adaptive response to H_2_S will provide new insight into mechanisms that can improve homeostasis in changing conditions.

Our finding that SKN-1 plays a role in the response to H_2_S is consistent with a recent report that nuclear accumulation of NRF2, a mammalian homologue of SKN-1, is correlated with H_2_S-induced protection against from ischemia-induced heart failure [30]. SKN-1 controls the Phase II response to toxins and oxidative stress [25]. However, we do not favour the hypothesis that H_2_S activates the Phase II response. The canonical Phase II targets *gst-4* nor *gcs-1* were not dramatically induced by H_2_S, and the H_2_S-induced upregulation of another glutathione S-transferase, *gst-19*, was exaggerated in *skn-1(RNAi)* animals. Moreover, we observed little overlap in genes regulated by *skn-1* in response to xenobiotic or oxidative stress and those that are changed in H_2_S. Instead, we found at least 7 of the gene products included in the F-box and BTB/POZ clusters (Table 3) were identified as *skn-1*-dependent zygotic transcripts [23]. Finally, we did not observe obvious accumulation of *skn-1::GFP* in the intestinal nuclei of animals exposed to H_2_S using epifluoresence microscopy (not shown), although we cannot rule out the possibility that the nuclear enrichment of GFP was below the detection limit in this experiment. We suggest that, during adaptation to H_2_S, *skn-1* may play a role in remodeling the protein turnover machinery.

Protein homeostasis is increasingly appreciated for its importance to aging and age-associated decline [32]. We propose that one consequence of adaptation to H_2_S is to increase transcription of genes related to protein turnover by the ubiquitin ligase and proteasome system, including F-box and BTB/POZ domain proteins. In this model, the effect of H_2_S to increase lifespan and thermotolerance may be attributed, at least in part, from effects on protein homeostasis. Further understanding the mechanisms by which adaptation to H_2_S can improve homeostasis and influence lifespan may provide novel insights into the mechanisms that mediate the beneficial effects of H_2_S in mammals.

## Materials and Methods

### Nematode strains and culture

Strains used were N2 wild-type (Bristol), ZG31 *hif-1(ia04)*, and EU35 *skn-1(zu169)/nT1[unc-?(n754) let-?] (IV;V).* EU35 and RNAi strains mentioned below were a gift from Dr. Jim Priess (Division of Basic Sciences, Fred Hutchinson Cancer Research Center, Seattle, WA). The *ia04* mutation deletes the second through fourth exons of HIF-1 and is a predicted molecular null [9]. *zu169* is an ochre mutation in an exon of *skn-1* shared among all isoforms. The *zu169* mutation is maternal effect lethal [33], abrogates paraquat-induced expression of *gst-4* and *gcs-1* in the intestine [25] and prevents increased lifespan in response to dietary restriction [26].

*C. elegans* were grown on nematode growth medium plates seeded with live *Escherichia coli* OP50 food (NGM/OP50 plates) as described previously [34]. All experiments and worm culturing were conducted at room temperature to avoid effects resulting from changing temperature. Exposure to H_2_S was in continuous flow H_2_S chambers that were created as previously described [7], by continuously diluting 5000 ppm H_2_S/balance N_2_ (Airgas, Seattle, WA USA) with house air to a final concentration of 50 ppm H_2_S. For viability assays, worms were exposed to H_2_S as fourth-stage larvae (L4) and scored for survival after 18–24 h. The effect of *skn-1(zu169)* mutations on viability in H_2_S was determined by picking sterile, non-Unc progeny of *skn-1*/*nT1* animals, and compared to Unc heterozygous siblings. To cross the *zu169* allele away from the *nT1* balancer, N2 males were crossed with *skn-1/nT1* mutant hermaphrodites. Non-Unc heterozygous F1 were allowed to produce F2 progeny, which were scored for sensitivity to H_2_S and the *skn-1* maternal effect lethal phenotype.

*skn-1(RNAi)* animals were generated by feeding N2 from starved L1 on HT115(DE3) bacteria carrying either the *skn-1* clone or empty vector control (L4440) from the Ahringer library [35]. RNAi by feeding was essentially as described [35]. Bacteria expressing the dsRNA was diluted from an overight culture grown in LB containing 25 mM carbenicillin and 10 mM tetracycline, regrown to OD_600_∼0.6 in LB with 25 mM carbenicillin and then seeded onto NGM-lite plates that contained 3 mM isopropyl β-D-1-thiogalactopyranoside and 25 mM carbenicillin. RNAi plates were allowed to dry overnight, stored at 4 C, and used within 5 days of being seeded. *skn-1(RNAi)* adults laid only dead embryos.

### RNA sample isolation

For microarray and qRT-PCR experiments, animals were synchronized as starved first-stage larvae (L1) after isolating embryos by hypochlorite treatment. For microarray analysis, 3000 starved L1 were distributed onto 15 cm NGM/OP50 plates, with each independent replicate performed on a different day. For quantitative RT-PCR (qRT-PCR), 1000 L1 larvae were distributed onto 10 cm NGM/OP50 plates. *C. elegans* were exposed to H_2_S on plates for the amount of time indicated immediately prior to harvest. All animals were harvested as first-day gravid adults (schematized in Figure 1A). For nematode harvest, plates were removed from H_2_S, the worms were immediately rinsed off the plates with distilled water, caught on a 43-micron nylon filter and collected by centrifugation. 100 µL of sedimented worms were added to 900 µL Trizol (Invitrogen, Carlsbad, CA), frozen in liquid nitrogen and stored at −70 C. Less than 2 min elapsed from when plates were removed from H_2_S until samples were frozen. Frozen samples were thawed, vortexed for 30 s, and the RNA was isolated following the protocol included with the Trizol manual, followed by isopropanol precipitation.

### Microarray expression profiling and analysis

Each RNA sample was labelled, hybridized to a single-channel Nimblegen 4×72 K (build 160) expression array, and scanned following manufacturer's suggested protocols by the Fred Hutchinson Cancer Research Center's DNA Array Facility. Three biological replicates for each H_2_S-treated sample (1, 12 and 48 h exposure) and 5 biological replicates from untreated controls were used. Data were RMA normalized and probe-level data were summarized with the NimbleScan software. Genes with weak signal intensity across all groups and those with low variability across samples were excluded from further analysis. Each H_2_S-treated sample was statistically compared to a matched untreated control using the Bioconductor package *limma* [36]. The false discovery rate (FDR) method of Benjamini and Hochberg [37] was used to adjust p-values for multiple testing. An adjusted p-value≤0.05 was used to define differential expression. Results were annotated using WormBase WS190 (www.wormbase.org). Expression results and microarray raw intensity files, in compliance with MIAME guidelines, can be accessed through the Gene Expression Omnibus (www.ncbi.nlm.nih.gov/geo/) and are accessible through GEO series accession number GSE25199. Functional annotation clustering analysis was performed using the Database for Annotation, Visualization and Integrated Discovery (DAVID) v6.7 (http://david.abcc.ncifcrf.gov/home.jsp). Gene list submitted for 12 h exposure to H_2_S included only the 91 gene products with logFC>1.6 (fold change>3). For the 48 h dataset, the 95 gene products with logFC>2.5 (fold change>5.5) were included. In both cases, the *C. elegans* background list in the database was used with default analysis parameters. Using only gene products included in the analysis after filtering did not alter the results. Annotation clusters that included at least one term with p<0.05 were considered to be functionally enriched clusters.

Hypergeometric probabilities were calculated including all 11,522 features included in the microarray analysis as the population, with successes and sample size as defined in the text (http://stattrek.com/tables/hypergeometric.aspx). The number of successes in each sample (overlap) was determined by manually comparing data from H_2_S-induced changes measured by our microarray experiments and hypoxia-induced genes [15] or *skn-1*-dependent transcripts [27], [28]. Probabilities less than 0.05 were considered significant. Core *skn-1* consensus sites in the promoter region of candidate transcripts were defined manually, based on the published consensus RTCAT [27]. The promoter region was defined as 2 kb upstream of the start site. The ModENCODE database [38] was searched to determine if any of the 7 transcripts changed in a *skn-1*-dependent changes in response to H_2_S were shown to have SKN-1::GFP bound in the promoter.

### Real-time RT-PCR (qRT-PCR)

Quantitative RT-PCR (qRT-PCR) was used to validate microarray measurements and determine if H_2_S-dependent changes occurred in *hif-1(ia04)* or *skn-1(RNAi)* animals. mRNA was isolated as described above, and cDNA was synthesized from 300 ng of RNA using the included random primers using the ProtoScript M-MuLV First Strand cDNA Synthesis Kit (New England Biolabs) according to manufacturer's suggested protocol. Primers to amplify cDNA targets were designed using Primer3 (http://frodo.wi.mit.edu/primer3/). When possible, primer pairs spanned a small intron so that genomic and cDNA amplification products could be distinguished by agarose gel electrophoresis. Primer sequences are available upon request. Primers were tested to ensure amplification of the correct size genomic target, and then calibrated against serial dilutions of genomic DNA. qRT-PCR reactions were performed using an *ep realplex^2^* S (Eppendorf). Each 10 µL reaction contained 5 µL 2X KAPA SYBR green Master Mix (Kapa Biosystems), 0.45 µL cDNA and 3 µL primers (10 µM each primer). Reactions were performed in duplicate and at least three independent biological replicates were included for each condition. Each experiment included primers that amplified only genomic DNA (negative controls to identify background signal levels) as well as 4 control targets (*sir-2.1*, *tba-1*, *irs-2*, and *hil-1*) that are not affected by H_2_S exposure for normalization. ΔC_t_ for each gene product was calculated by subtracting C_t_ values from the geometric mean of the control targets [39]. ΔC_t_ were averaged across the three experiments. Student's t-test was used to evaluate differences between ΔC_t_ values of treated samples and untreated controls (EXCEL). For differences between genotypes (Figures 2 and 3), p-values were calculated by one-way Anova from summary statistics (mean, standard deviation, n) (www.statpages.org). Reported fold-changes were calculated as 2∧−ΔΔC_t_ [40], where ΔΔC_t_ = ΔC_t_(H_2_S)−ΔC_t_(untreated). Error bars on graphs represent standard deviation, which was carried through the fold-change calculation using standard error propagation (reported as “variance”).

## Supporting Information

Table S1 Transcripts that are significantly changed after 12 h exposure to H2S, listed in order of magnitude fold-change.
(PDF)

Table S2 Transcripts that are significantly changed after 48 h exposure to H2S, listed in order of magnitude fold-change.
(PDF)

Table S3 Genes included in functional annotation clusters.
(PDF)

Table S4 Transcripts altered by both 1 h exposure to H2S and hypoxia.
(PDF)

Table S5 Transcripts regulated by *skn-1* in other studies that are altered by exposure to H_2_S.
(PDF)
