# Supplementary material for: HIF-1 and SKN-1 Coordinate the Transcriptional Response to Hydrogen Sulfide in Caenorhabditis elegans
Source: PLoS One. 2011 Sep 29;6(9):e25476. doi: 10.1371/journal.pone.0025476 (PMC3183046; doi:10.1371/journal.pone.0025476)
Supplement: Table S1 — Transcripts that are significantly changed after 12 h exposure to H2S, listed in order of magnitude fold-change. (PDF) [file pone.0025476.s001.pdf]

Supporting Table 1: Significant changes after 12h in H_2S			
gene	logFC (12hr)	fold-change	adj.P.Val (12hr)
F37B1.8	6.05	66.43	4.21E-06
R08F11.4	4.81	28.14	4.08E-04
F02H6.5	3.03	8.17	1.72E-03
T24B8.5	2.85	7.19	3.01E-03
Y41C4A.17	2.76	6.79	0.005
F58E6.7	2.72	6.57	0.005
F15B9.1	2.70	6.49	0.005
K11G9.6	2.66	6.33	0.005
ZK899.6	2.63	6.17	0.004
R08E5.1	2.60	6.05	0.003
Y110A2AL.3	2.45	5.48	0.004
F46C5.1	2.38	5.19	0.012
C52D10.7	2.31	4.97	0.007
F18E3.2	2.27	4.81	0.010
C03E10.6	2.23	4.70	0.005
M162.5.1	2.23	4.69	0.005
C45G7.2	2.20	4.59	0.049
ZC412.6	2.20	4.58	0.013
Y22D7AR.10	2.18	4.53	0.010
H12D21.1	2.17	4.50	0.006
R09E12.9	2.07	4.19	0.007
H12D21.13	2.05	4.14	0.011
H12D21.12	2.03	4.08	0.008
F01D5.3	2.01	4.03	0.029
C54F6.5	2.00	4.00	0.011
C34E11.4	2.00	3.99	0.011
H12D21.14	1.99	3.98	0.011
Y50E8A.17	1.99	3.98	0.013
Y27F2A.8	1.98	3.95	0.011
K10G6.5	1.97	3.92	0.010
ZK849.6	1.97	3.91	0.011
F22E5.20	1.94	3.83	0.012
C10G8.4	1.94	3.83	0.028
C06A5.12	1.90	3.74	0.012
Y110A2AL.9	1.90	3.73	0.012
Y48G1C.9.1	1.90	3.72	0.012
ZK1251.2	1.89	3.70	0.012
F59F4.2.1	1.88	3.67	0.011
F44F1.7	1.86	3.62	0.012
H23N18.2	1.86	3.62	0.013
F12E12.7	1.85	3.61	0.012
T28A11.22	1.81	3.50	0.016
C08F11.12	1.79	3.46	0.016
C08F1.3	1.79	3.46	0.014
Y57G11C.51	1.79	3.45	0.016
F26D10.12	1.77	3.41	0.017
Y60A9.3	1.77	3.40	0.014

Supporting Table 1: Significant changes after 12h in H_2S			
gene	logFC (12hr)	fold-change	adj.P.Val (12hr)
Y46B2A.1	1.77	3.40	0.016
Y46H3A.3	1.76	3.38	0.028
F34D10.8	1.73	3.33	0.015
B0213.6	1.73	3.32	0.018
T05B4.1	1.73	3.31	0.020
Y51H7C.8	1.73	3.31	0.017
T09A12.5.2	1.73	3.31	0.020
C44C10.8	1.72	3.30	0.020
M05D6.6.2	1.72	3.28	0.020
C17E7.12	1.72	3.28	0.029
F53G2.8	1.71	3.28	0.018
Y116A8C.19	1.71	3.27	0.020
Y51B9A.4	1.71	3.27	0.016
Y48G9A.7	1.71	3.26	0.016
E03H4.10	1.70	3.26	0.048
T27E4.3	1.70	3.26	0.032
C40H5.1	1.70	3.24	0.026
F40B5.1	1.69	3.23	0.049
K02D7.6	1.68	3.21	0.018
F09G8.7	1.68	3.20	0.020
C33A12.7	1.67	3.19	0.020
ZK1058.6	1.67	3.19	0.018
C31C9.2.1	1.67	3.18	0.018
R52.1	1.66	3.16	0.018
F31A9.2	1.66	3.16	0.020
W06A7.5	1.65	3.15	0.018
C53H9.3	1.65	3.15	0.023
F59A6.12	1.65	3.14	0.020
F36D1.4	1.65	3.14	0.022
F49F1.1	1.65	3.14	0.033
R02E12.6.1	1.65	3.14	0.030
C31G12.1	1.64	3.13	0.018
F56F10.3	1.64	3.12	0.021
F15A4.10	1.64	3.12	0.037
B0252.8	1.64	3.11	0.034
F55H12.2	1.63	3.10	0.039
F10G2.4	1.63	3.10	0.021
Y37H2A.10	1.62	3.08	0.020
F01D5.1	1.62	3.07	0.026
C54F6.7	1.61	3.06	0.020
K07A1.15	1.61	3.06	0.029
F56G4.2	1.61	3.06	0.020
F02D10.6	1.61	3.05	0.028
F58D5.2a	1.61	3.04	0.025
R11G1.7	1.60	3.02	0.020
K10D2.4	1.59	3.01	0.020
Y39F10B.1a	1.59	3.00	0.049

Supporting Table 1: Significant changes after 12h in H_2S			
gene	logFC (12hr)	fold-change	adj.P.Val (12hr)
Y45G12B.3	1.59	3.00	0.047
T09A12.5.1	1.58	2.99	0.027
C16C8.5	1.58	2.99	0.020
F37B1.5	1.58	2.99	0.037
C38D4.1b	1.58	2.99	0.020
F31C3.5	1.58	2.98	0.027
F36H1.5	1.58	2.98	0.042
Y82E9BR.17b	1.57	2.97	0.023
D1025.6	1.57	2.97	0.020
T05C12.5	1.56	2.95	0.027
Y38E10A.14	1.55	2.93	0.027
Y38H6C.16	1.55	2.93	0.023
F59F4.2.2	1.55	2.92	0.021
M01D1.3	1.55	2.92	0.028
Y75B8A.18	1.55	2.92	0.029
B0304.5	1.55	2.92	0.028
C13G3.1	1.54	2.92	0.022
T22B7.5	1.54	2.92	0.034
F23F12.12	1.54	2.91	0.028
F15A4.2	1.54	2.91	0.030
K07C6.5	1.54	2.90	0.045
M01D1.8	1.53	2.89	0.029
M04D8.3	1.53	2.89	0.028
R02C2.6	1.53	2.89	0.032
Y71H2B.4	1.53	2.89	0.038
F58D5.2b.2	1.53	2.88	0.035
W04E12.2	1.52	2.87	0.045
F58D5.2b.1	1.52	2.87	0.029
M05D6.6.1	1.52	2.87	0.026
F54E7.5	1.52	2.86	0.027
K08H10.9	1.51	2.86	0.029
Y25C1A.6.1	1.51	2.84	0.033
C17F3.1	1.51	2.84	0.028
T22G5.6	1.51	2.84	0.036
Y71G12B.27	1.50	2.84	0.027
Y37D8A.25	1.50	2.82	0.030
M116.1	1.50	2.82	0.031
T28D6.9	1.50	2.82	0.046
F46B6.12.1	1.49	2.82	0.042
C49A9.10	1.49	2.81	0.028
Y116A8B.1	1.49	2.81	0.049
Y92C3B.1	1.49	2.81	0.026
Y82E9BL.5	1.49	2.80	0.028
Y39G10AR.15	1.49	2.80	0.028
Y45F10C.2	1.48	2.80	0.026
W09G12.1	1.48	2.79	0.029
R12E2.8	1.48	2.78	0.027

Supporting Table 1: Significant changes after 12h in H_2S			
gene	logFC (12hr)	fold-change	adj.P.Val (12hr)
Y51H7BR.1	1.48	2.78	0.037
T03D8.7	1.47	2.77	0.028
F23F1.5	1.47	2.76	0.031
Y46G5A.8	1.46	2.76	0.029
Y106G6H.16	1.46	2.76	0.042
T27E4.4	1.46	2.76	0.027
C52E2.1	1.46	2.76	0.027
Y54G2A.23.1	1.46	2.75	0.028
F35E12.3	1.46	2.75	0.029
K06A4.1	1.45	2.74	0.040
F40H6.4	1.45	2.74	0.029
Y46E12A.4	1.45	2.73	0.035
ZK381.3.2	1.45	2.72	0.049
F28D1.2	1.44	2.72	0.028
Y45G5AM.5	1.44	2.71	0.029
Y56A3A.10	1.44	2.71	0.029
C34B2.2	1.44	2.71	0.029
T03F7.3	1.43	2.70	0.030
F11A5.15	1.43	2.70	0.030
Y51H7BR.2	1.43	2.70	0.030
D1025.8	1.43	2.69	0.028
K10H10.2.1	1.43	2.69	0.029
ZK616.3	1.42	2.68	0.030
T04G9.7	1.42	2.67	0.029
Y11D7A.10.1	1.41	2.66	0.038
R03A10.1	1.41	2.66	0.033
F08F1.4a	1.41	2.65	0.046
F23A7.8	1.41	2.65	0.029
Y11D7A.10.2	1.41	2.65	0.030
F19B10.2	1.40	2.65	0.036
D1025.4	1.40	2.65	0.032
C44C1.3	1.40	2.64	0.047
T12A2.3	1.40	2.63	0.046
F46B6.12.2	1.40	2.63	0.045
C44B12.3	1.39	2.62	0.033
K08F4.7	1.39	2.62	0.040
F02H6.4	1.39	2.62	0.049
R10A10.2	1.38	2.61	0.030
Y47G6A.31	1.38	2.61	0.035
F08F1.4b.2	1.38	2.61	0.046
C52E2.6	1.38	2.59	0.036
C14B1.8	1.37	2.59	0.041
W07A12.7	1.37	2.59	0.045
C45B11.5	1.37	2.59	0.037
Y73F4A.3	1.37	2.59	0.037
W03G9.8	1.37	2.58	0.049
F55C9.3	1.37	2.58	0.042

Supporting Table 1: Significant changes after 12h in H_2S

gene	logFC (12hr)	fold-change	adj.P.Val (12hr)
B0416.7a	1.37	2.58	0.032
T05G5.4	1.36	2.58	0.039
ZK1098.11	1.36	2.58	0.042
T08B6.5	1.36	2.58	0.037
Y71G12B.28	1.36	2.57	0.036
F15D3.6	1.36	2.57	0.045
Y39B6A.9	1.36	2.57	0.035
C29F3.6	1.36	2.56	0.037
C45G9.7	1.35	2.55	0.049
C38D4.9.1	1.35	2.55	0.036
F38C2.5	1.35	2.55	0.046
R08H2.1	1.35	2.54	0.036
K11D12.13	1.35	2.54	0.045
B0416.4	1.34	2.53	0.037
F09C8.1	1.34	2.52	0.035
F22E5.21	1.33	2.51	0.046
C28F5.1	1.33	2.51	0.041
B0304.4	1.33	2.51	0.035
C26B2.7	1.33	2.51	0.042
Y47H9C.1	1.33	2.51	0.035
F41G3.4	1.32	2.50	0.043
C46C2.3	1.32	2.50	0.037
F08D12.10	1.32	2.50	0.037
Y105C5B.13	1.32	2.50	0.044
Y52E8A.2	1.32	2.50	0.044
W02D9.7	1.32	2.50	0.044
F19B10.9	1.32	2.49	0.035
F57A10.4	1.31	2.49	0.038
Y48G8AL.15	1.31	2.49	0.035
B0281.5	1.31	2.48	0.039
T11F8.2	1.30	2.47	0.037
F22D3.5	1.30	2.47	0.041
F59H6.4	1.30	2.46	0.046
C34B7.3	1.30	2.46	0.042
F45F2.9.1	1.30	2.45	0.040
F42F12.9	1.29	2.45	0.037
D1025.7	1.29	2.45	0.044
F49F1.5	1.29	2.44	0.047
C06E7.6	1.29	2.44	0.046
C25E10.8	1.29	2.44	0.047
Y46H3A.2	1.28	2.44	0.046
T21C9.8	1.28	2.42	0.047
F57C2.4	1.28	2.42	0.039
F11C7.7	1.27	2.42	0.043
Y58A7A.1	1.27	2.41	0.045
T28C6.3	1.27	2.41	0.040
Y51H4A.4	1.27	2.41	0.044

**Supporting Table 1: Significant changes after 12h in H_2S**			
gene	logFC (12hr)	fold-change	adj.P.Val (12hr)
T10F2.5	1.27	2.40	0.046
F01D5.5	1.27	2.40	0.049
F23B2.13	1.27	2.40	0.049
Y43F11A.1	1.27	2.40	0.049
F45C12.13	1.25	2.39	0.045
Y73C8C.2	1.25	2.38	0.044
F23A7.4	1.25	2.37	0.045
T10B9.10	1.24	2.37	0.049
F42A9.8	1.24	2.36	0.044
C50F4.4	1.24	2.36	0.044
F42F12.10	1.24	2.36	0.044
ZK673.5	1.24	2.35	0.047
T12D8.5.1	1.24	2.35	0.047
F54D10.1	1.23	2.35	0.047
C05B5.2	1.23	2.35	0.049
F16G10.11	1.23	2.34	0.048
E02H9.7	1.23	2.34	0.044
F25H9.7	1.21	2.32	0.050
B0393.8	1.21	2.31	0.047
Y48A6C.3	1.20	2.30	0.047
C38D4.1a	1.19	2.29	0.049
F52F12.8	1.19	2.28	0.050
F59D12.5	1.18	2.27	0.049
Y47D7A.1.2	1.18	2.26	0.049
F07F6.6	-1.18	-2.26	0.049
F47A4.1b	-1.19	-2.27	0.049
F49C5.3	-1.19	-2.29	0.049
C29A12.4	-1.20	-2.30	0.047
B0350.2g.2	-1.21	-2.31	0.049
Y23B4A.1	-1.21	-2.31	0.046
ZK783.1	-1.21	-2.32	0.047
T09B9.2	-1.22	-2.32	0.047
F15B9.7	-1.22	-2.32	0.046
F54E2.3c	-1.22	-2.33	0.048
F52H3.7a	-1.22	-2.33	0.046
C05E11.4	-1.22	-2.33	0.045
T19D12.1	-1.22	-2.33	0.045
ZK973.6	-1.23	-2.34	0.049
F22E10.1	-1.24	-2.37	0.042
F22E10.3.1	-1.24	-2.37	0.047
F15A2.6	-1.25	-2.38	0.047
Y4C6B.2a	-1.25	-2.39	0.049
T05A10.1h.1	-1.26	-2.39	0.041
F25C8.1	-1.26	-2.39	0.046
Y95B8A.10	-1.26	-2.39	0.042
F36H2.5	-1.26	-2.40	0.048
F53B7.5	-1.27	-2.42	0.041

Supporting Table 1: Significant changes after 12h in H_2S			
gene	logFC (12hr)	fold-change	adj.P.Val (12hr)
F39B3.3	-1.28	-2.42	0.046
K11D12.10b	-1.28	-2.44	0.042
F45D11.1.2	-1.29	-2.44	0.044
Y102A11A.2b	-1.29	-2.44	0.039
B0350.2g.1	-1.29	-2.45	0.046
H12I19.4	-1.29	-2.45	0.046
F46C3.3	-1.29	-2.45	0.038
T22H6.6	-1.30	-2.46	0.040
B0019.1	-1.30	-2.46	0.046
F25G6.7a	-1.30	-2.47	0.045
Y34B4A.10.2	-1.30	-2.47	0.046
M60.5	-1.31	-2.47	0.041
F14F9.3	-1.32	-2.50	0.037
Y54G2A.25a	-1.33	-2.51	0.035
F46B6.10	-1.33	-2.51	0.038
F52F10.3	-1.33	-2.52	0.041
C08F1.5b	-1.34	-2.53	0.033
ZK524.2a	-1.34	-2.53	0.035
F26F2.1	-1.35	-2.55	0.043
F54E2.3a	-1.35	-2.55	0.049
F09C3.1	-1.35	-2.55	0.040
F54D12.3	-1.35	-2.56	0.036
T02C5.5d.1	-1.36	-2.57	0.042
B0207.1	-1.36	-2.57	0.042
F53B1.9	-1.36	-2.57	0.047
C15H9.1	-1.37	-2.58	0.042
T06D8.1b	-1.37	-2.59	0.031
C44C10.1	-1.38	-2.60	0.048
Y43F8B.14	-1.38	-2.60	0.033
K10G6.4	-1.38	-2.61	0.044
Y57G11C.21	-1.38	-2.61	0.043
C14F5.3c.2	-1.38	-2.61	0.034
F59A2.6	-1.39	-2.62	0.041
C29F9.13	-1.39	-2.62	0.047
T06D8.1a	-1.39	-2.62	0.031
Y53C10A.10	-1.39	-2.62	0.041
F45H11.4	-1.40	-2.63	0.033
T19D12.4b	-1.40	-2.64	0.032
F57C12.1	-1.40	-2.64	0.042
K09F6.5	-1.40	-2.64	0.029
T19D2.1	-1.41	-2.65	0.029
B0280.12a	-1.41	-2.67	0.035
H39E23.3	-1.41	-2.67	0.039
Y70D2A.1	-1.42	-2.67	0.035
K11D12.4	-1.42	-2.68	0.035
T12G3.1.1	-1.43	-2.70	0.029
Y65B4BR.6a	-1.44	-2.72	0.046

Supporting Table 1: Significant changes after 12h in H_2S			
gene	logFC (12hr)	fold-change	adj.P.Val (12hr)
C34G6.6b	-1.45	-2.72	0.046
C38C6.6.1	-1.45	-2.73	0.030
F13D11.2b	-1.45	-2.73	0.033
F25G6.6	-1.46	-2.74	0.029
F55H12.3	-1.46	-2.75	0.029
F20D1.4	-1.46	-2.75	0.027
C24A11.2	-1.46	-2.76	0.037
D2005.6	-1.46	-2.76	0.027
C30D11.3	-1.46	-2.76	0.042
Y50E8A.16	-1.47	-2.76	0.029
F45D11.1.1	-1.47	-2.76	0.030
R31.1	-1.47	-2.77	0.042
Y43D4A.5	-1.47	-2.77	0.049
F33D4.2g	-1.48	-2.79	0.027
R11A5.7.1	-1.48	-2.79	0.028
F07C3.1	-1.49	-2.80	0.028
M195.1	-1.50	-2.84	0.035
C41H7.7	-1.50	-2.84	0.042
F58A3.2b.3	-1.51	-2.84	0.037
F56D3.1.2	-1.51	-2.85	0.035
F58F6.1	-1.51	-2.85	0.027
C01G5.4	-1.52	-2.86	0.032
R10D12.1	-1.52	-2.87	0.029
F10D11.6	-1.52	-2.87	0.049
C15A11.5	-1.53	-2.88	0.046
F15G9.4a	-1.53	-2.88	0.032
C38C3.7	-1.53	-2.89	0.042
C23F12.1a	-1.53	-2.89	0.039
K07E3.1	-1.54	-2.90	0.029
F32G8.5	-1.54	-2.90	0.032
T10E9.3	-1.55	-2.94	0.046
Y51A2D.4.1	-1.56	-2.95	0.036
C31A11.5	-1.57	-2.96	0.028
C17B7.12	-1.57	-2.97	0.027
C16E9.1	-1.57	-2.97	0.020
F45E4.3a	-1.58	-2.99	0.041
C38C6.6.2	-1.58	-2.99	0.027
Y39B6A.24.1	-1.58	-2.99	0.020
C43C3.1	-1.59	-3.00	0.021
C43G2.2	-1.59	-3.02	0.020
F23B2.12.1	-1.60	-3.03	0.020
ZC247.1	-1.61	-3.05	0.035
T01D3.3b	-1.61	-3.05	0.027
C54D2.5b	-1.61	-3.05	0.020
F35B3.4	-1.62	-3.08	0.040
C14F5.3b	-1.63	-3.09	0.020
ZK180.5c	-1.64	-3.11	0.043

Supporting Table 1: Significant changes after 12h in H_2S			
gene	logFC (12hr)	fold-change	adj.P.Val (12hr)
K10D11.2	-1.64	-3.11	0.037
C41D7.2	-1.64	-3.11	0.020
F22B8.5	-1.65	-3.13	0.027
F22E10.2	-1.65	-3.13	0.018
ZK617.1a.1	-1.65	-3.14	0.042
R09B5.5	-1.65	-3.14	0.029
H43E16.1	-1.65	-3.14	0.020
C23H3.7	-1.65	-3.15	0.020
ZK377.1	-1.66	-3.16	0.044
ZK180.5b.1	-1.66	-3.16	0.040
F57B7.3	-1.66	-3.16	0.020
T12G3.1.2	-1.66	-3.17	0.020
Y102A5C.21	-1.67	-3.18	0.018
Y102A11A.2a	-1.67	-3.18	0.020
C18H7.11	-1.67	-3.19	0.029
T19A5.3a	-1.68	-3.20	0.042
T05B4.12	-1.69	-3.22	0.033
Y57A10A.11	-1.69	-3.23	0.041
C18H7.1.2	-1.70	-3.25	0.023
C42D4.3	-1.72	-3.30	0.020
T20D4.4	-1.72	-3.30	0.015
T10E10.6	-1.75	-3.35	0.020
M195.2	-1.75	-3.37	0.046
T10H4.11	-1.76	-3.39	0.030
C49G7.5	-1.78	-3.44	0.020
T01D3.1	-1.79	-3.46	0.020
T10H10.1	-1.79	-3.46	0.016
W05G11.3.1	-1.79	-3.46	0.020
F42C5.10	-1.80	-3.47	0.018
C25F6.4	-1.80	-3.49	0.013
ZK1067.7	-1.81	-3.50	0.049
F45E4.3b.2	-1.82	-3.53	0.036
T24E12.5	-1.82	-3.54	0.013
T19A5.3b	-1.83	-3.55	0.046
C49G7.7	-1.85	-3.59	0.018
F15G9.4b	-1.85	-3.61	0.046
C18H7.1.1	-1.85	-3.61	0.016
E01G4.6	-1.86	-3.63	0.045
ZK678.5.2	-1.87	-3.64	0.041
T21E8.1b	-1.87	-3.65	0.012
F21C10.7	-1.87	-3.66	0.020
F28F8.2.2	-1.88	-3.69	0.011
B0350.2f.1	-1.90	-3.72	0.020
F54D1.3	-1.90	-3.74	0.031
F14F9.4	-1.92	-3.79	0.015
T28A11.20	-1.93	-3.81	0.012
C29F3.2	-2.01	-4.03	0.026

Supporting Table 1: Significant changes after 12h in H_2S			
gene	logFC (12hr)	fold-change	adj.P.Val (12hr)
M02D8.4b.1	-2.01	-4.03	0.010
F36G9.12	-2.06	-4.16	0.011
C05E4.9a.1	-2.06	-4.18	0.010
ZK180.5a	-2.07	-4.20	0.012
T06E4.11	-2.08	-4.24	0.035
F54D1.2	-2.09	-4.27	0.029
C03A7.8	-2.11	-4.31	0.020
M02D8.4a	-2.11	-4.31	0.007
Y47D7A.15	-2.14	-4.41	0.013
B0350.2f.2	-2.15	-4.45	0.013
C03A7.4	-2.16	-4.48	0.020
Y47D7A.13.1	-2.19	-4.57	0.016
C03A7.7	-2.20	-4.58	0.026
F44G3.2	-2.23	-4.69	0.012
M02D8.4c	-2.28	-4.86	0.005
T21E8.1a	-2.33	-5.02	0.006
T20D4.5	-2.35	-5.10	0.007
T01D1.6	-2.49	-5.62	0.020
T18H9.1	-2.52	-5.73	0.011
H23L24.5	-2.73	-6.63	0.003
C05A9.1	-2.83	-7.12	0.005
T20D4.3	-2.87	-7.33	0.003
