# Supplementary material for: HIF-1 and SKN-1 Coordinate the Transcriptional Response to Hydrogen Sulfide in Caenorhabditis elegans
Source: PLoS One. 2011 Sep 29;6(9):e25476. doi: 10.1371/journal.pone.0025476 (PMC3183046; doi:10.1371/journal.pone.0025476)
Supplement: Table S2 — Transcripts that are significantly changed after 48 h exposure to H2S, listed in order of magnitude fold-change. (PDF) [file pone.0025476.s002.pdf]

Supporting Table 2: Significant changes after 48h in H_2S

gene	logFC (48hr)	fold-change	adj.P.Val (48hr)
F37B1.8	5.46	44.04	1.8E-09
R08F11.4	5.27	38.64	9.5E-09
ZK899.6	4.67	25.48	6.8E-09
F26D10.12	4.66	25.25	5.4E-09
C52D10.7	4.65	25.05	6.8E-09
F12E12.7	4.54	23.34	9.5E-09
C08F1.3	4.44	21.77	2.8E-08
Y82E9BR.17b	4.38	20.79	6.8E-09
C08F1.10	4.36	20.53	6.8E-09
C25B8.4a	4.22	18.60	1.3E-08
M01D1.8	4.20	18.38	6.8E-09
F08D12.10	4.09	17.04	3.9E-08
K11G9.6	4.09	17.01	9.8E-09
F44F1.7	4.08	16.96	2.8E-08
C25B8.4c	4.05	16.58	1.1E-08
R52.1	3.93	15.24	3.7E-08
T04B8.3	3.89	14.80	1.2E-07
C27A7.8	3.76	13.57	6.7E-08
Y45G5AM.5	3.76	13.56	3.1E-08
K06A4.1	3.73	13.23	4.4E-08
Y40B1B.3	3.72	13.18	1.2E-07
Y116A8C.19	3.70	13.04	1.2E-07
F22E5.20	3.65	12.57	3.7E-08
F12E12.10	3.64	12.48	9.5E-08
Y51H7BR.2	3.64	12.45	1.8E-07
W09G12.1	3.60	12.14	3.7E-08
C52E2.1	3.59	12.04	8.4E-08
F56G4.2	3.56	11.80	3.8E-08
Y56A3A.10	3.53	11.57	1.2E-07
C38D9.8	3.52	11.48	3.7E-07
Y57A10C.1	3.50	11.31	8.0E-06
F38C2.5	3.39	10.47	6.0E-07
F45C12.13	3.34	10.11	4.0E-07
C41H7.8	3.34	10.11	8.6E-08
Y27F2A.8	3.32	9.98	1.5E-07
Y51H7BR.1	3.32	9.98	2.8E-07
Y46E12A.4	3.31	9.95	1.6E-07
F31E9.4	3.26	9.59	2.6E-07
F15B9.1	3.25	9.54	1.4E-07
ZC204.9	3.17	8.98	3.0E-07
Y46G5A.28	3.15	8.86	2.2E-06
M116.1	3.13	8.78	5.5E-07
F45C12.6	3.12	8.70	3.6E-07
W09G12.4	3.11	8.63	1.8E-07
F15E11.12	3.10	8.56	2.5E-06
T24B8.5	3.07	8.41	5.5E-07
K05F6.5	3.06	8.34	6.1E-06

Supporting Table 2: Significant changes after 48h in H_2S			
gene	logFC (48hr)	fold-change	adj.P.Val (48hr)
M116.4	3.03	8.16	8.9E-07
F40B5.1	2.97	7.82	6.1E-06
M01E5.6	2.96	7.79	1.8E-06
Y46B2A.1	2.95	7.71	1.5E-06
F28D1.2	2.94	7.68	4.7E-07
C45G7.3	2.91	7.53	8.8E-07
R09E12.9	2.89	7.39	2.1E-06
C43D7.5	2.88	7.35	4.8E-06
M01D1.3	2.85	7.20	9.2E-07
Y110A2AL.3	2.83	7.10	6.0E-07
Y60A9.3	2.83	7.09	7.5E-07
Y22D7AR.10	2.82	7.06	6.7E-07
Y56A3A.14	2.82	7.06	6.8E-07
C03E10.6	2.82	7.05	6.7E-07
Y51B9A.4	2.80	6.97	7.6E-07
C08F11.12	2.79	6.93	8.0E-07
F53C3.2	2.79	6.90	6.8E-07
C10G8.4	2.78	6.89	8.3E-06
Y73C8C.8	2.77	6.83	1.2E-05
Y82E9BR.17a	2.77	6.82	1.2E-05
C52E2.7	2.76	6.78	8.8E-07
F58E6.7	2.73	6.65	2.2E-06
C44C10.8	2.73	6.63	9.0E-07
Y44A6C.2	2.72	6.61	1.5E-06
F15E11.15a	2.72	6.61	2.7E-06
F02H6.5	2.72	6.60	1.0E-06
F40G9.9	2.72	6.58	4.8E-06
Y46G5A.27	2.72	6.57	7.7E-06
B0304.5	2.71	6.56	2.2E-06
Y45F10C.2	2.70	6.48	1.2E-06
Y51H4A.4	2.69	6.44	1.9E-06
F55C9.4	2.67	6.36	3.6E-06
ZC204.8	2.63	6.18	7.9E-06
Y41C4A.17	2.61	6.13	1.9E-05
R11A5.3	2.61	6.11	1.9E-06
Y39G8B.7	2.61	6.09	2.1E-06
C24H11.3	2.60	6.07	7.3E-06
C06A5.12	2.59	6.01	1.9E-06
F23F12.9a	2.59	6.01	3.4E-06
F58E1.11	2.57	5.92	3.6E-06
Y105C5B.13	2.55	5.87	2.0E-06
Y57A10C.11	2.55	5.86	3.9E-06
T28A11.22	2.53	5.80	2.7E-06
C40A11.10	2.53	5.78	2.1E-06
Y63D3A.3	2.53	5.78	1.3E-05
C52E2.6	2.53	5.78	3.1E-06
Y43F11A.1	2.53	5.78	2.2E-06

Supporting Table 2: Significant changes after 48h in H_2S			
gene	logFC (48hr)	fold-change	adj.P.Val (48hr)
R08C7.13	2.53	5.77	7.0E-06
F40H6.4	2.50	5.65	3.1E-06
T22B7.5	2.50	5.65	1.2E-05
M01D1.10	2.49	5.63	3.4E-06
Y82E9BL.5	2.49	5.62	2.8E-06
F12E12.4	2.49	5.62	3.9E-06
C08A9.7	2.48	5.59	3.2E-06
R12E2.8	2.48	5.57	3.2E-06
T05D4.2	2.48	5.57	2.6E-06
F15E11.13	2.46	5.50	2.6E-05
F43G6.5	2.46	5.50	5.5E-06
F08D12.9	2.45	5.46	4.5E-06
Y48G1C.9.1	2.45	5.46	4.4E-06
T08B6.5	2.45	5.45	5.2E-06
C37E2.4	2.42	5.37	1.1E-04
C54F6.5	2.41	5.31	3.8E-06
F55C9.13	2.39	5.25	2.0E-05
F54E7.5	2.39	5.24	4.5E-06
C32B5.14	2.39	5.23	1.2E-05
Y71G12B.28	2.38	5.21	5.9E-06
T12D8.5.1	2.37	5.19	5.3E-06
C45G7.2	2.37	5.18	1.9E-05
F02D10.6	2.37	5.15	6.7E-06
R07C3.5	2.36	5.15	1.2E-05
R02C2.6	2.36	5.15	5.2E-06
F38H4.2	2.35	5.11	2.6E-05
F45C12.5	2.35	5.11	1.8E-05
F39F10.4	2.35	5.10	9.3E-06
R09B5.9	2.35	5.09	2.1E-03
K07C6.5	2.35	5.09	1.3E-05
Y48G9A.7	2.35	5.09	5.1E-06
F31A9.2	2.34	5.07	7.7E-06
R07C3.6	2.34	5.06	5.5E-06
F14B6.3	2.34	5.06	5.5E-06
F15A4.2	2.34	5.05	1.2E-05
C04F6.3.1	2.33	5.03	1.3E-05
C34E11.4	2.33	5.03	7.5E-06
F19F10.5	2.33	5.03	1.0E-04
Y25C1A.6.1	2.31	4.97	5.5E-06
F08D12.8	2.31	4.96	7.0E-06
K05F6.7	2.30	4.92	7.0E-05
Y56A3A.15	2.30	4.92	7.3E-06
F56F4.1	2.30	4.91	1.5E-05
C32B5.16	2.30	4.91	1.8E-05
Y49F6C.3	2.29	4.90	7.7E-06
K05F6.3	2.28	4.87	2.2E-05
C31G12.1	2.28	4.86	7.4E-06

Supporting Table 2: Significant changes after 48h in H_2S			
gene	logFC (48hr)	fold-change	adj.P.Val (48hr)
ZK381.3.2	2.27	4.82	2.6E-05
C27B7.9	2.27	4.81	1.1E-05
F28C6.1	2.26	4.78	4.8E-05
F12E12.8	2.25	4.77	4.2E-05
T22G5.6	2.25	4.76	1.7E-05
F55C9.3	2.24	4.74	1.7E-05
R08E5.1	2.23	4.69	1.2E-05
T03F7.3	2.23	4.68	9.3E-06
F35E12.3	2.22	4.65	9.9E-06
W08F4.2	2.22	4.65	1.1E-05
W06A11.2	2.22	4.65	3.7E-05
K02E7.9	2.22	4.64	1.2E-05
T22B2.1	2.21	4.62	1.1E-04
T26E3.8	2.21	4.62	7.2E-05
F19B10.9	2.21	4.61	1.1E-05
D1007.10a	2.20	4.61	1.3E-05
T26E4.15	2.20	4.59	2.7E-05
T07C4.2	2.20	4.58	4.4E-05
Y19D10B.7	2.19	4.58	8.1E-05
Y110A2AL.9	2.19	4.56	1.2E-05
C52D10.9.1	2.18	4.54	1.3E-05
F18E3.2	2.18	4.52	1.2E-05
T10B9.10	2.17	4.50	1.1E-04
T04D3.2	2.17	4.49	4.6E-04
F10E7.1	2.17	4.49	1.2E-05
Y46G5A.8	2.16	4.48	1.2E-05
B0432.1	2.16	4.46	1.8E-05
Y106G6H.16	2.16	4.46	7.7E-05
F53G2.8	2.16	4.46	1.2E-05
Y37H2A.10	2.16	4.46	2.0E-05
F48E3.6	2.16	4.45	1.5E-05
F14B6.1	2.15	4.45	1.6E-05
Y116A8A.1	2.14	4.42	3.7E-05
K07A1.15	2.14	4.40	3.0E-05
R11.4	2.13	4.37	1.5E-05
B0047.1b	2.12	4.34	2.1E-05
Y116A8B.1	2.12	4.33	5.8E-05
ZK1240.4	2.11	4.32	1.9E-05
F09G8.7	2.11	4.32	2.1E-05
W04E12.2	2.11	4.30	8.5E-05
C06B3.5	2.10	4.30	2.7E-05
Y71G12B.20a	2.10	4.30	1.6E-05
R11G1.7	2.10	4.29	2.2E-05
Y47D7A.1.2	2.10	4.28	1.8E-05
F49F1.1	2.09	4.27	5.2E-05
T09B4.5a.2	2.09	4.27	1.8E-05
F09C8.1	2.09	4.27	2.3E-05

Supporting Table 2: Significant changes after 48h in H_2S			
gene	logFC (48hr)	fold-change	adj.P.Val (48hr)
K10D2.4	2.09	4.26	2.0E-05
T12A2.3	2.09	4.25	1.9E-05
ZK673.10	2.09	4.25	4.7E-05
F17E9.2	2.09	4.24	1.7E-05
K02B12.2	2.07	4.21	1.8E-05
F11A5.15	2.07	4.21	2.2E-05
C46C2.3	2.07	4.20	2.5E-05
D2096.5	2.07	4.19	2.2E-05
F59A6.12	2.06	4.18	2.1E-05
F58E1.3	2.06	4.18	2.1E-05
Y38H6C.16	2.06	4.18	2.0E-05
C39B5.2	2.06	4.17	4.0E-05
M03A1.3	2.06	4.16	2.1E-05
K10G6.5	2.06	4.16	2.9E-05
K08H2.1	2.05	4.14	2.0E-05
F01D5.3	2.04	4.12	4.6E-05
F19H8.4	2.04	4.12	1.1E-04
F57A10.4	2.04	4.12	4.3E-05
T25E12.6	2.04	4.11	4.2E-05
F23F1.5	2.04	4.11	2.2E-05
Y49F6C.4	2.04	4.11	2.3E-05
F23A7.8	2.04	4.11	3.3E-05
R02C2.1	2.03	4.10	6.2E-05
C24H12.11	2.03	4.10	6.2E-05
M02F4.9	2.02	4.07	5.1E-05
C16C8.5	2.02	4.07	2.2E-05
K08H2.4	2.02	4.06	2.4E-05
ZK381.3.1	2.02	4.06	2.6E-05
F19B10.2	2.02	4.06	3.0E-05
T09B4.5b	2.02	4.05	2.4E-05
R05G9.3	2.02	4.05	3.4E-05
T26H2.3	2.01	4.04	2.6E-04
K02D7.6	2.01	4.02	2.7E-05
M151.8	2.00	4.01	3.6E-04
DC2.7a	2.00	4.01	2.7E-05
F22D3.5	2.00	4.00	2.6E-05
F54A5.3a	1.99	3.98	5.8E-05
ZK673.5	1.98	3.96	2.8E-05
Y105E8B.4	1.98	3.95	4.9E-05
F15A4.10	1.98	3.94	5.0E-05
K05F6.4	1.98	3.93	5.1E-05
F54D10.1	1.97	3.92	5.8E-05
F46B6.12.1	1.97	3.92	1.9E-04
Y57G11C.51	1.97	3.91	4.8E-05
B0041.3	1.97	3.91	3.6E-05
DC2.7b	1.97	3.91	6.6E-05
C34F11.9c	1.97	3.91	5.1E-05

Supporting Table 2: Significant changes after 48h in H_2S			
gene	logFC (48hr)	fold-change	adj.P.Val (48hr)
F46B6.12.2	1.96	3.90	5.4E-05
F55C9.8	1.96	3.90	6.0E-04
F42A9.8	1.96	3.89	3.6E-05
C16A11.6	1.96	3.89	4.0E-05
Y57G7A.2	1.96	3.88	6.1E-05
Y105C5B.20	1.96	3.88	3.4E-05
C02F12.5	1.95	3.88	5.6E-05
Y92C3B.1	1.95	3.87	3.4E-05
B0281.4	1.95	3.85	4.9E-05
EEED8.13	1.94	3.85	4.8E-05
F53B6.9	1.94	3.83	4.8E-04
C17E7.12	1.93	3.82	1.1E-04
C09H5.5	1.93	3.81	4.9E-05
F29A7.1	1.93	3.81	4.9E-05
T24C2.2	1.93	3.81	9.5E-05
E02H9.9	1.93	3.81	1.0E-04
T09A12.5.2	1.93	3.81	5.1E-05
C40A11.4	1.93	3.80	4.7E-05
F02H6.4	1.92	3.79	3.7E-05
F15B9.2	1.92	3.78	4.1E-05
F54D12.4	1.92	3.78	9.2E-05
F23F12.12	1.92	3.78	6.7E-05
C44B12.3	1.92	3.78	3.8E-05
B0416.4	1.92	3.78	5.1E-05
F08B6.1	1.92	3.77	7.4E-05
F13A7.9	1.92	3.77	4.5E-05
C40A11.6	1.91	3.77	7.2E-05
E02H9.7	1.91	3.76	4.8E-05
C33F10.13	1.90	3.74	8.3E-05
Y46H3C.7	1.90	3.74	4.6E-04
T09A12.5.1	1.90	3.73	5.6E-05
K02E2.4	1.89	3.72	4.7E-05
Y11D7A.8	1.89	3.71	1.7E-04
F44G4.3	1.89	3.70	1.1E-04
F07B10.2	1.89	3.70	7.7E-05
ZC204.7	1.88	3.69	1.2E-04
Y116A8C.22	1.88	3.69	8.2E-05
T05E7.5	1.88	3.68	5.2E-05
F09E10.1	1.88	3.68	1.6E-04
C49G7.8	1.88	3.68	4.4E-04
D1025.6	1.88	3.68	5.1E-05
C49A9.10	1.88	3.67	7.5E-05
F52C6.3	1.88	3.67	6.0E-05
F53B2.6	1.88	3.67	8.6E-05
Y62E10A.4	1.88	3.67	6.9E-05
F49B2.2	1.88	3.67	8.8E-05
F08F1.4a	1.87	3.67	9.6E-05

Supporting Table 2: Significant changes after 48h in H_2S			
gene	logFC (48hr)	fold-change	adj.P.Val (48hr)
K09H11.10	1.87	3.66	5.2E-05
ZK1251.2	1.87	3.66	5.7E-05
F19B10.7	1.87	3.65	5.1E-05
F46C5.1	1.87	3.65	3.8E-03
Y69A2AR.29	1.87	3.65	2.3E-04
C13G3.1	1.87	3.64	5.1E-05
T10F2.5	1.87	3.64	6.3E-05
C54F6.7	1.87	3.64	5.1E-05
F58D5.2a	1.86	3.64	8.6E-05
Y75B8A.33	1.86	3.64	5.1E-05
F14D2.11	1.86	3.63	9.5E-05
T05H4.1	1.86	3.63	5.8E-05
T24H7.4	1.86	3.63	9.5E-05
W04A8.5	1.86	3.63	6.5E-05
C29F9.12	1.86	3.62	7.2E-05
F32H2.6	1.86	3.62	5.4E-05
F37B1.5	1.85	3.61	1.2E-04
C13F10.2	1.85	3.60	6.6E-05
B0281.6	1.85	3.60	1.1E-04
C50E3.13	1.85	3.60	5.7E-05
F26D10.11	1.84	3.59	5.6E-05
T19C3.6	1.84	3.58	9.6E-05
F35D2.3	1.84	3.58	6.5E-05
Y39G10AR.5	1.84	3.58	1.3E-04
K09D9.2	1.84	3.57	3.6E-04
Y13C8A.1	1.84	3.57	6.3E-04
T22B2.2	1.83	3.57	8.6E-05
F10G2.4	1.83	3.56	1.1E-04
Y38E10A.3	1.83	3.55	8.1E-05
C48B6.9	1.83	3.55	9.5E-05
Y47G6A.31	1.83	3.55	8.1E-05
F12E12.5	1.83	3.55	1.4E-04
B0303.8	1.83	3.55	1.8E-04
ZK616.3	1.83	3.55	7.9E-05
F57B1.6b	1.83	3.55	6.7E-05
Y45G12B.3	1.83	3.55	6.4E-05
ZK1248.19	1.82	3.54	1.1E-04
Y71A12B.2	1.82	3.54	5.0E-04
K09E3.5	1.82	3.54	1.1E-04
F59D12.5	1.82	3.53	8.6E-05
C17E7.3	1.82	3.53	8.1E-05
ZK909.4	1.82	3.53	1.1E-04
F59F4.2.2	1.82	3.53	6.5E-05
F23B12.9	1.81	3.52	1.3E-04
M162.5.1	1.81	3.50	7.3E-05
D2096.1	1.80	3.49	8.6E-05
C44B12.6	1.80	3.49	1.9E-04

Supporting Table 2: Significant changes after 48h in H_2S			
gene	logFC (48hr)	fold-change	adj.P.Val (48hr)
T02H6.9	1.80	3.49	1.2E-04
H23N18.2	1.80	3.49	1.1E-04
T12G3.7	1.80	3.48	8.6E-05
F40E10.1	1.80	3.48	1.0E-04
K02B7.1	1.80	3.47	1.1E-04
R03A10.1	1.80	3.47	9.2E-05
Y41D4B.10	1.80	3.47	8.4E-05
F07D3.2	1.79	3.47	1.3E-04
C07E3.9	1.79	3.47	1.2E-04
D2085.2	1.79	3.47	1.3E-04
F13A7.10	1.79	3.46	1.1E-04
K05F6.1	1.79	3.46	4.4E-04
Y38E10A.14	1.79	3.46	1.6E-04
C17D12.1a	1.79	3.45	1.1E-04
T26E3.7	1.79	3.45	1.1E-04
F23D12.4	1.79	3.45	9.7E-05
C45H4.14a	1.79	3.45	9.6E-05
T19C4.8	1.78	3.44	1.1E-04
C34B4.4	1.78	3.43	1.1E-04
K08F4.12	1.77	3.42	9.5E-05
Y39F10B.1a	1.77	3.41	1.4E-04
K08H10.9	1.77	3.41	1.7E-04
Y57G11C.25	1.76	3.40	1.3E-04
Y75B8A.18	1.76	3.39	1.2E-04
C47F8.1	1.76	3.39	1.3E-04
C46C2.5	1.76	3.38	1.2E-04
C52A10.2	1.76	3.38	3.6E-04
C46A5.8	1.75	3.37	1.5E-04
Y47D3A.12	1.75	3.37	1.8E-04
B0281.5	1.75	3.36	1.0E-04
F08F1.4b.2	1.75	3.36	1.3E-04
Y49F6B.7	1.75	3.36	2.2E-04
F13A2.4	1.75	3.35	1.0E-04
C05E11.6	1.74	3.35	1.8E-04
ZK616.2	1.74	3.35	1.8E-04
F57B1.6a	1.74	3.35	2.2E-04
T14G12.4a	1.74	3.34	2.7E-03
Y52B11A.7	1.74	3.34	9.7E-05
Y41D4A.3	1.74	3.33	1.2E-04
F27C8.4	1.74	3.33	1.1E-04
F58E1.13	1.73	3.33	1.3E-04
Y47D7A.1.1	1.73	3.33	1.3E-04
Y94H6A.4	1.73	3.32	1.0E-04
Y116A8C.1	1.73	3.32	9.7E-05
R08C7.9	1.73	3.32	3.4E-04
DC2.7c	1.73	3.32	1.3E-04
C45G9.7	1.73	3.32	1.5E-04

Supporting Table 2: Significant changes after 48h in H_2S			
gene	logFC (48hr)	fold-change	adj.P.Val (48hr)
T28C6.3	1.73	3.32	1.0E-04
B0304.4	1.73	3.31	1.1E-04
F35G12.7	1.73	3.31	2.6E-04
F52D2.8a	1.73	3.31	1.1E-04
D2063.1	1.72	3.30	3.0E-04
F40G9.6	1.72	3.29	2.9E-04
D1025.8	1.72	3.29	1.1E-04
C50F4.4	1.72	3.29	1.2E-04
F15B10.3	1.72	3.29	2.0E-04
F59F4.2.1	1.71	3.28	1.1E-04
Y67A10A.4	1.71	3.28	1.3E-04
C31H5.4.2	1.71	3.27	1.4E-04
F55H12.2	1.71	3.27	1.2E-03
Y18D10A.17.2	1.71	3.27	2.8E-04
D1025.7	1.71	3.27	1.2E-04
C33E10.2	1.71	3.27	2.3E-04
C17C3.8	1.71	3.26	2.5E-04
Y46G5A.7	1.71	3.26	1.5E-04
T24D5.6	1.70	3.26	7.5E-04
T11F8.2	1.70	3.26	1.1E-04
F45D11.9	1.70	3.26	1.2E-04
F52B11.6	1.70	3.25	2.5E-04
F09C3.3	1.70	3.25	2.0E-04
Y87G2A.18	1.70	3.25	2.4E-04
F58D5.2b.1	1.70	3.25	1.8E-04
C47G2.8	1.70	3.24	1.7E-04
W08E12.6	1.70	3.24	1.6E-04
R10A10.2	1.70	3.24	1.2E-04
F08G5.5.2	1.70	3.24	2.6E-04
F08G5.5.1	1.69	3.24	2.7E-04
F28H7.4	1.69	3.23	1.2E-04
C40A11.7	1.69	3.22	1.9E-04
ZK287.6	1.68	3.21	2.8E-04
F19H6.5	1.68	3.21	1.9E-04
T07A5.5	1.68	3.21	2.7E-04
K08D12.4	1.68	3.21	2.2E-04
K09C4.1b	1.68	3.21	3.5E-04
Y71H2AR.3	1.68	3.20	1.3E-04
ZC84.7	1.68	3.20	2.0E-04
F32D1.11	1.68	3.20	1.4E-04
C17E4.4	1.68	3.20	4.7E-04
C31H5.4.1	1.68	3.20	1.5E-04
Y55F3BR.5	1.68	3.20	2.4E-04
Y48G8AL.15	1.67	3.19	1.4E-04
F14D7.7	1.67	3.19	1.7E-04
F14H3.5	1.67	3.18	2.4E-04
F54E7.9.1	1.67	3.18	1.5E-04

Supporting Table 2: Significant changes after 48h in H_2S			
gene	logFC (48hr)	fold-change	adj.P.Val (48hr)
M01G5.6	1.67	3.18	2.3E-04
H25K10.1	1.67	3.17	1.4E-04
Y39G10AR.15	1.66	3.17	2.0E-04
F40B1.1	1.66	3.16	1.4E-04
C31C9.4	1.66	3.16	1.9E-04
K01H12.1	1.66	3.15	3.1E-04
F54A5.3b	1.66	3.15	4.1E-04
R13F6.1	1.66	3.15	1.5E-04
F45B8.1	1.66	3.15	2.6E-04
ZK250.2	1.65	3.15	1.8E-04
F45E10.2b	1.65	3.15	1.5E-04
C29F3.6	1.65	3.15	1.6E-04
K11D2.4b	1.65	3.15	2.8E-04
H24O09.2	1.65	3.14	3.4E-04
F56A4.12	1.65	3.14	2.2E-04
Y48G9A.6	1.65	3.13	1.7E-04
Y52E8A.2	1.65	3.13	1.8E-04
T04G9.7	1.64	3.13	1.9E-04
T13B5.6	1.64	3.13	2.5E-04
R09B5.8	1.64	3.12	3.6E-04
T05G5.4	1.64	3.12	2.0E-04
Y47H9C.1	1.64	3.12	3.4E-04
Y49F6C.5	1.64	3.12	1.6E-04
C14B1.8	1.64	3.12	5.4E-04
T08B1.6	1.64	3.11	2.3E-04
C42D4.6	1.64	3.11	1.8E-04
F26D11.1	1.64	3.11	2.1E-04
F46A8.7	1.64	3.11	3.4E-04
F35H10.5	1.64	3.11	2.3E-04
R04D3.4	1.64	3.11	2.0E-04
K06G5.2	1.63	3.10	2.4E-04
K05F6.9	1.63	3.10	2.0E-04
C17F4.8	1.63	3.10	2.4E-04
ZC204.14	1.63	3.10	2.6E-04
R02E12.6.1	1.63	3.10	4.7E-04
F56D5.5	1.63	3.09	1.9E-04
ZK1251.7	1.62	3.08	4.6E-04
H16D19.4	1.62	3.08	2.1E-04
F59H6.9	1.62	3.08	1.8E-04
Y55B1AL.2	1.62	3.08	3.5E-04
Y32G9A.13	1.62	3.08	1.8E-04
F48C1.9	1.62	3.07	2.2E-04
Y49F6C.8	1.62	3.07	2.2E-04
F26F4.13	1.62	3.07	4.4E-04
F55G1.2	1.62	3.07	2.2E-04
B0495.6	1.62	3.06	2.5E-04
F19B10.8	1.61	3.06	2.8E-04

Supporting Table 2: Significant changes after 48h in H_2S			
gene	logFC (48hr)	fold-change	adj.P.Val (48hr)
C12D8.12	1.61	3.05	2.1E-04
F36D1.9	1.61	3.05	3.0E-04
F12E12.1	1.61	3.05	2.7E-04
F56B3.3	1.60	3.04	2.3E-04
F19C7.3	1.60	3.04	4.2E-04
H16D19.1	1.60	3.04	6.8E-04
Y113G7B.8	1.60	3.03	2.4E-04
K11D12.13	1.60	3.03	3.9E-04
F47H4.1	1.60	3.03	2.0E-04
C04H5.7	1.60	3.03	5.9E-04
K09H9.7	1.60	3.03	2.5E-04
F15D3.6	1.60	3.03	2.3E-04
ZK892.6	1.59	3.02	2.3E-04
W03D8.7	1.59	3.02	2.2E-04
B0047.3	1.59	3.01	2.2E-04
M110.5a.1	1.59	3.01	3.4E-04
F54F7.2	1.59	3.01	3.6E-04
T27A1.2	1.59	3.01	2.8E-04
T02G6.5	1.59	3.01	3.0E-04
C33A12.7	1.59	3.00	3.1E-04
C10C6.7	1.59	3.00	2.1E-04
T21C9.8	1.58	2.99	3.1E-04
W05B5.4	1.58	2.99	2.5E-04
Y82E9BR.22	1.58	2.99	4.0E-04
C24D10.5	1.58	2.99	3.6E-04
C16C8.4	1.58	2.99	8.2E-04
M01B2.7	1.58	2.98	6.1E-04
F35H8.7	1.58	2.98	1.4E-03
Y37E11AR.6	1.58	2.98	2.8E-04
F38G1.2	1.58	2.98	2.7E-04
B0336.13	1.58	2.98	2.6E-04
K04G2.11	1.57	2.98	2.4E-04
C16C8.11	1.57	2.98	3.6E-04
C53H9.3	1.57	2.97	2.3E-04
F02E11.4	1.57	2.97	2.3E-04
Y111B2A.10a	1.57	2.97	3.7E-04
ZK1098.11	1.57	2.97	2.7E-04
C34B2.2	1.57	2.96	3.0E-04
B0252.8	1.57	2.96	3.6E-04
T21C12.7	1.57	2.96	3.7E-04
B0416.2	1.56	2.96	2.7E-04
F58A4.14	1.56	2.96	3.5E-04
K09A11.3	1.56	2.95	2.6E-04
T23B5.4	1.56	2.95	6.5E-04
R06C7.6	1.56	2.95	5.1E-04
T28D6.9	1.56	2.95	5.6E-04
F12E12.3	1.56	2.95	2.8E-04

Supporting Table 2: Significant changes after 48h in H_2S			
gene	logFC (48hr)	fold-change	adj.P.Val (48hr)
R10H10.6	1.56	2.95	2.8E-04
F40C5.1	1.56	2.95	3.0E-04
M01F1.1	1.56	2.95	2.8E-04
T13F2.8.1	1.56	2.95	3.1E-04
M02B7.4	1.56	2.94	2.8E-04
Y79H2A.11	1.56	2.94	3.6E-04
Y37D8A.25	1.56	2.94	2.9E-04
F53A2.2	1.56	2.94	3.5E-04
C50F4.6	1.56	2.94	5.8E-04
C32B5.1a	1.56	2.94	2.8E-04
Y48A6C.3	1.55	2.94	2.8E-04
C10A4.1	1.55	2.93	4.9E-04
W07E6.5	1.55	2.93	4.5E-04
B0213.6	1.55	2.93	4.0E-04
Y19D10A.11	1.55	2.93	4.2E-04
Y37E11B.6	1.55	2.93	5.9E-04
K07E8.10	1.55	2.93	2.8E-04
C49C8.3	1.55	2.93	2.8E-04
C50E3.12	1.55	2.93	2.6E-04
Y39B6A.9	1.55	2.93	3.1E-04
F48F5.4	1.55	2.93	3.6E-04
Y18H1A.6	1.55	2.92	5.9E-04
F54D8.1.2	1.55	2.92	5.3E-04
R07E5.11	1.55	2.92	3.6E-04
C11G6.2	1.54	2.92	3.7E-03
M02D8.6	1.54	2.91	7.3E-04
F46F11.4	1.54	2.91	6.3E-04
F39F10.3	1.54	2.91	2.9E-04
F23D12.2	1.54	2.91	2.8E-04
M04B2.3	1.54	2.91	6.8E-04
C06A5.9	1.54	2.91	3.1E-04
Y47D3A.1	1.54	2.91	3.0E-04
C25H3.6c	1.54	2.90	3.4E-04
B0393.8	1.54	2.90	3.4E-04
D1025.4	1.54	2.90	3.0E-04
T13C2.7	1.54	2.90	3.4E-04
F45C12.12	1.54	2.90	3.1E-04
T16G12.4	1.53	2.90	3.1E-04
ZK546.15	1.53	2.89	4.4E-04
R04A9.7	1.53	2.89	3.5E-04
F12A10.1	1.53	2.89	4.4E-04
Y75B12B.4	1.53	2.88	4.1E-04
F32H2.2	1.53	2.88	3.7E-04
ZK1058.6	1.53	2.88	6.1E-04
C08A9.10	1.53	2.88	2.1E-03
B0035.18	1.53	2.88	3.0E-04
K08F9.1	1.53	2.88	1.0E-03

Supporting Table 2: Significant changes after 48h in H_2S			
gene	logFC (48hr)	fold-change	adj.P.Val (48hr)
C08F8.9	1.52	2.88	4.1E-04
F55G1.7	1.52	2.88	4.0E-04
C38D4.1b	1.52	2.87	5.0E-04
Y39F10B.1b.1	1.52	2.87	4.5E-04
W02D7.5	1.52	2.87	3.4E-04
ZK353.9	1.52	2.87	4.4E-04
F25H9.7	1.52	2.87	3.5E-04
C06G3.8	1.52	2.86	4.4E-04
T24E12.11	1.52	2.86	5.0E-04
Y39B6A.42	1.52	2.86	8.0E-04
F35G12.11.2	1.51	2.86	4.4E-04
EEED8.4	1.51	2.86	3.6E-04
C52E2.3	1.51	2.86	3.4E-04
F52C6.4	1.51	2.85	5.5E-04
T05F1.10	1.51	2.85	6.8E-04
C46E10.8	1.51	2.85	3.5E-04
F40G9.4	1.51	2.85	3.7E-04
Y41D4B.14	1.51	2.85	3.4E-04
Y51H7C.12	1.51	2.84	3.4E-04
T27A8.2	1.51	2.84	4.6E-04
T20G5.10	1.51	2.84	1.0E-03
F46F11.7	1.51	2.84	4.1E-04
F17E9.1	1.50	2.84	8.4E-04
C02A12.4	1.50	2.83	4.0E-04
Y82E9BR.1	1.50	2.83	4.7E-04
K02A2.2	1.50	2.83	8.1E-04
F55G1.11	1.50	2.83	3.8E-04
H14E04.1	1.50	2.82	7.2E-04
D1022.3	1.50	2.82	9.3E-04
C32B5.6	1.49	2.82	6.7E-04
W05B10.3	1.49	2.82	7.0E-04
Y73F8A.22	1.49	2.81	3.6E-04
F17A2.5	1.49	2.81	5.0E-04
C42C1.12	1.49	2.81	9.6E-04
C10B5.1	1.49	2.81	1.0E-03
Y40B10A.6	1.49	2.80	8.0E-03
Y54G2A.23.1	1.49	2.80	3.8E-04
Y52E8A.1	1.49	2.80	5.3E-04
Y50E8A.3	1.49	2.80	5.5E-04
D1007.4	1.48	2.80	1.0E-03
C36F7.2	1.48	2.80	4.5E-04
C38D4.9.1	1.48	2.79	4.5E-04
C08B6.8	1.48	2.79	9.6E-04
F21A3.6	1.48	2.79	3.8E-04
K01G12.3	1.48	2.79	5.9E-04
T22C8.4	1.48	2.79	1.2E-03
M05D6.6.1	1.48	2.79	4.1E-04

Supporting Table 2: Significant changes after 48h in H_2S

gene	logFC (48hr)	fold-change	adj.P.Val (48hr)
Y34F4.2	1.48	2.78	6.5E-04
T10C6.10	1.48	2.78	4.5E-04
Y48G1BM.8	1.48	2.78	1.0E-03
F52H2.2	1.47	2.78	4.7E-04
Y39G10AL.1	1.47	2.78	7.4E-04
Y71G12B.20b	1.47	2.77	5.0E-04
C40A11.2.1	1.47	2.77	4.4E-04
C08F1.11	1.47	2.77	8.5E-04
Y51H7C.3	1.47	2.77	5.7E-04
K02E2.7	1.47	2.77	6.8E-04
M199.4	1.47	2.77	7.9E-04
C50F2.10	1.47	2.76	6.2E-04
Y69H2.2	1.47	2.76	6.7E-04
Y43F4B.10	1.46	2.76	7.5E-04
T06A10.3	1.46	2.76	5.3E-04
F57C2.4	1.46	2.75	4.6E-04
F58B3.9	1.46	2.75	6.5E-04
F09C3.4	1.46	2.75	9.8E-04
Y66D12A.5	1.46	2.75	6.2E-04
F27B3.6	1.46	2.75	5.5E-04
F35G12.11.1	1.46	2.75	4.8E-04
F40F8.9.1	1.46	2.74	7.0E-04
W02D7.4	1.46	2.74	5.5E-04
F35G2.3	1.45	2.74	5.1E-04
R52.8	1.45	2.74	8.5E-04
F31E9.6	1.45	2.74	7.3E-04
EEED8.12	1.45	2.73	5.3E-04
F58D5.2b.2	1.45	2.73	7.1E-04
T16H12.2	1.45	2.73	7.9E-04
Y23H5B.7a	1.45	2.73	6.2E-04
Y51H7BM.1	1.45	2.73	8.4E-04
C25H3.3	1.45	2.73	7.4E-04
Y71F9B.4	1.45	2.73	1.5E-03
F57C2.2	1.45	2.72	4.7E-04
Y110A2AL.4b	1.45	2.72	5.2E-04
F45E10.2a	1.44	2.72	7.5E-04
Y71H2B.4	1.44	2.72	1.3E-03
Y97E10AR.3	1.44	2.72	1.3E-03
Y39B6A.34	1.44	2.72	1.3E-03
C36A4.11	1.44	2.72	7.6E-04
Y68A4A.13	1.44	2.71	8.1E-04
C06H2.7	1.44	2.71	5.2E-04
T22H2.5a.2	1.44	2.71	6.1E-04
C35E7.5a	1.44	2.71	7.8E-04
F01D5.1	1.44	2.71	5.7E-04
C05C8.1	1.44	2.71	6.7E-04
F55A12.10	1.43	2.70	2.6E-03

Supporting Table 2: Significant changes after 48h in H_2S			
gene	logFC (48hr)	fold-change	adj.P.Val (48hr)
Y47D3A.20	1.43	2.70	1.9E-03
R07H5.4	1.43	2.70	5.7E-04
C49C3.6	1.43	2.70	1.1E-03
F55A3.5	1.43	2.70	6.7E-04
F45G2.7	1.43	2.70	6.0E-04
Y55D5A.6	1.43	2.69	5.3E-04
K09F6.8	1.43	2.69	8.1E-04
W09B6.4a	1.43	2.69	6.1E-04
T02G5.1	1.43	2.69	5.3E-04
Y49E10.22	1.42	2.68	1.4E-03
EEED8.2	1.42	2.68	5.7E-04
F33E2.5.1	1.42	2.68	1.2E-03
T03F1.12	1.42	2.68	8.4E-04
K01A12.4	1.42	2.68	1.1E-03
ZK836.3	1.42	2.68	8.9E-04
F09F7.6	1.42	2.68	7.3E-04
F53G2.2	1.42	2.68	6.3E-04
T07C4.4.2	1.42	2.68	8.2E-04
Y82E9BL.17	1.42	2.67	2.5E-03
T14G12.4b.1	1.42	2.67	3.1E-03
C46C11.4	1.41	2.67	7.6E-04
K06B9.6	1.41	2.67	7.4E-04
F40A3.1	1.41	2.66	8.0E-04
JC8.4	1.41	2.66	6.3E-04
B0047.2	1.41	2.66	7.1E-04
Y39H10A.2	1.41	2.66	8.7E-04
F16G10.11	1.41	2.66	6.2E-04
Y51H7C.8	1.41	2.66	6.2E-04
T05E12.3	1.41	2.65	6.7E-04
K07F5.9	1.41	2.65	2.1E-03
Y57G11C.45	1.41	2.65	9.2E-04
W03C9.3.2	1.40	2.65	6.2E-04
F18E2.5	1.40	2.65	2.0E-03
K08E4.2	1.40	2.64	8.2E-04
T24H10.1	1.40	2.64	6.2E-04
F36H1.5	1.40	2.64	1.6E-03
Y75B8A.11	1.40	2.64	9.6E-04
T27E9.6	1.40	2.64	6.6E-04
T07A5.6b	1.40	2.64	7.6E-04
T21C9.4	1.40	2.64	9.4E-04
F23A7.4	1.40	2.64	6.3E-04
C02B10.2	1.40	2.64	7.2E-04
F01F1.13	1.40	2.64	8.6E-04
T02C1.1	1.40	2.63	7.1E-04
C50B6.9	1.40	2.63	8.0E-04
Y38H6C.21	1.40	2.63	7.3E-04
C34B2.5	1.40	2.63	9.5E-04

Supporting Table 2: Significant changes after 48h in H_2S			
gene	logFC (48hr)	fold-change	adj.P.Val (48hr)
F36G9.8	1.39	2.63	9.5E-04
F02H6.1	1.39	2.63	8.5E-04
T27E9.8	1.39	2.63	2.6E-03
Y48G1C.6	1.39	2.63	1.1E-03
H20J04.9	1.39	2.63	2.0E-03
C48E7.10	1.39	2.62	1.1E-03
C17B7.7	1.39	2.62	7.6E-04
H14A12.5	1.39	2.62	8.9E-04
F36A2.8	1.39	2.62	8.3E-04
F23F1.10	1.39	2.62	9.3E-04
T26F2.2	1.39	2.62	8.4E-04
ZK688.7	1.39	2.61	8.6E-04
ZC53.7	1.39	2.61	9.0E-04
B0035.10	1.39	2.61	1.0E-03
C16C10.9	1.39	2.61	6.7E-04
F45F2.9.1	1.39	2.61	8.9E-04
F10E9.5	1.39	2.61	1.1E-03
C26B2.7	1.39	2.61	8.0E-04
F52D2.8b	1.39	2.61	8.6E-04
Y6G8.3	1.38	2.61	1.4E-03
F45C12.8	1.38	2.61	7.4E-04
F43C1.3	1.38	2.61	1.1E-03
Y24F12A.3	1.38	2.61	1.3E-03
T09A5.6	1.38	2.60	7.5E-04
C02B8.1.2	1.38	2.60	7.2E-04
C05D9.7	1.38	2.60	1.2E-03
ZK892.7	1.38	2.60	1.4E-03
W02B9.2	1.38	2.60	1.4E-03
K10D2.7	1.38	2.60	2.4E-03
K12H6.7	1.38	2.60	1.3E-03
F45B8.2	1.38	2.60	1.2E-03
T20G5.11.1	1.38	2.60	8.9E-04
W03H9.3	1.38	2.60	1.0E-03
F17A2.13	1.38	2.59	8.4E-04
ZC412.6	1.38	2.59	1.0E-03
F23B2.13	1.38	2.59	9.4E-04
R160.4	1.38	2.59	9.3E-04
C25H3.14.1	1.38	2.59	1.0E-03
Y111B2A.10b.1	1.37	2.59	9.1E-04
Y17G9A.4	1.37	2.59	1.1E-03
F44E2.9	1.37	2.59	9.5E-04
Y110A2AL.1	1.37	2.59	1.2E-03
B0336.8	1.37	2.59	1.7E-03
F28F8.3.1	1.37	2.59	1.1E-03
Y54G9A.9	1.37	2.59	9.7E-04
W09H1.4	1.37	2.59	1.0E-03
T08A9.12	1.37	2.59	9.3E-04

Supporting Table 2: Significant changes after 48h in H_2S			
gene	logFC (48hr)	fold-change	adj.P.Val (48hr)
F58B3.3	1.37	2.59	1.1E-03
ZK637.11	1.37	2.59	1.0E-03
R09B5.3.1	1.37	2.58	1.5E-03
F44B9.6	1.37	2.58	1.5E-03
F42F12.9	1.37	2.58	7.5E-04
Y39B6A.10	1.37	2.58	8.0E-04
F29B9.1	1.37	2.58	9.0E-04
ZK228.5	1.37	2.58	8.8E-04
K07C5.9	1.36	2.57	1.6E-03
F43G6.7	1.36	2.57	8.9E-04
Y116A8C.12	1.36	2.57	8.1E-04
M117.5	1.36	2.57	8.1E-04
T23G5.3	1.36	2.57	8.9E-04
F01F1.14	1.36	2.57	7.8E-04
F22B5.6	1.36	2.57	1.6E-03
B0222.4	1.36	2.57	1.8E-03
Y71F9AL.10	1.36	2.57	1.0E-03
Y41D4B.12a	1.36	2.57	1.0E-03
C28A5.4	1.36	2.56	9.5E-04
D1005.4	1.36	2.56	1.0E-03
D1005.5	1.36	2.56	1.6E-03
W06A11.4	1.36	2.56	8.3E-04
W03G9.8	1.36	2.56	1.2E-03
T09B4.5a.1	1.36	2.56	8.8E-04
F59E11.7a	1.35	2.56	8.2E-04
C15F1.8	1.35	2.55	1.1E-03
F53G12.1	1.35	2.55	8.9E-04
F45C12.7	1.35	2.55	8.6E-04
F54C9.3	1.35	2.55	1.1E-03
F40F11.3	1.35	2.55	1.3E-03
Y18D10A.16	1.35	2.55	1.0E-03
C38D4.1a	1.35	2.55	1.4E-03
F35C11.1	1.35	2.55	1.3E-03
F31C3.5	1.35	2.55	9.8E-04
F27C8.2	1.35	2.55	1.4E-03
F23D12.5	1.35	2.54	1.1E-03
F53F10.8	1.35	2.54	1.1E-03
Y39G10AR.20.2	1.35	2.54	1.4E-03
F26H11.1	1.35	2.54	9.3E-04
C25E10.8	1.34	2.54	1.1E-03
T04D3.8	1.34	2.54	2.0E-03
F57G4.9	1.34	2.54	9.1E-04
K12D12.5	1.34	2.54	1.1E-03
F08G12.10	1.34	2.54	8.7E-04
F53F4.16	1.34	2.53	1.1E-03
Y92C3B.3a	1.34	2.53	1.0E-03
C14A4.10	1.34	2.53	3.5E-03

Supporting Table 2: Significant changes after 48h in H_2S			
gene	logFC (48hr)	fold-change	adj.P.Val (48hr)
R03H10.6	1.34	2.52	1.1E-03
Y62E10A.17	1.33	2.52	1.1E-03
Y57A10A.9	1.33	2.52	1.0E-03
C18D4.6b	1.33	2.52	1.5E-03
C14C11.7	1.33	2.52	1.7E-03
C08E3.13	1.33	2.52	1.4E-03
F42H10.2	1.33	2.52	1.6E-03
B0213.15c	1.33	2.52	1.2E-03
M01D7.6.1	1.33	2.52	1.1E-03
K10D2.2.2	1.33	2.51	1.9E-03
F55G1.3	1.33	2.51	2.3E-03
C31C9.2.1	1.33	2.51	9.6E-04
F15E11.1	1.33	2.51	2.8E-03
F23H12.7	1.33	2.51	1.5E-03
C49F5.3	1.33	2.51	1.3E-03
C44C10.5	1.33	2.51	1.0E-03
F08F3.9b	1.32	2.50	1.3E-03
Y66D12A.7	1.32	2.50	1.8E-03
Y39B6A.40	1.32	2.50	1.6E-03
ZK666.4	1.32	2.50	9.6E-04
C34E10.7	1.32	2.50	1.1E-03
ZC196.2	1.32	2.50	1.9E-03
K11D2.1	1.32	2.50	1.0E-03
B0464.7.2	1.32	2.50	1.4E-03
T23B5.3c.2	1.32	2.50	1.2E-03
R07H5.1	1.32	2.50	1.5E-03
T02B5.1	1.32	2.50	4.9E-03
B0213.15a	1.32	2.49	1.3E-03
Y49E10.1	1.32	2.49	1.3E-03
Y106G6D.2	1.32	2.49	1.2E-03
F18A11.3	1.32	2.49	1.3E-03
C01H6.5d	1.31	2.49	1.6E-03
T01B7.3	1.31	2.49	1.4E-03
M02B7.1	1.31	2.48	1.9E-03
F53A2.9	1.31	2.48	1.3E-03
C24H11.9	1.31	2.48	1.2E-03
C48E7.11	1.31	2.48	1.4E-03
C36A4.10	1.31	2.48	1.3E-03
Y48G9A.8.2	1.31	2.48	1.1E-03
K08E4.3	1.31	2.48	1.4E-03
Y51A2D.14	1.31	2.48	2.1E-03
F21E9.3	1.31	2.47	1.2E-03
C17B7.13	1.31	2.47	1.3E-03
Y52B11A.9	1.30	2.47	1.1E-03
ZK652.3	1.30	2.47	1.6E-03
F21G4.4	1.30	2.47	1.2E-03
T10E9.7a	1.30	2.47	2.0E-03

Supporting Table 2: Significant changes after 48h in H_2S			
gene	logFC (48hr)	fold-change	adj.P.Val (48hr)
M176.3.2	1.30	2.46	1.4E-03
F45D3.1	1.30	2.46	2.5E-03
C53C11.2	1.30	2.46	1.3E-03
T05A10.2	1.30	2.46	1.8E-03
F56F10.3	1.30	2.46	1.9E-03
C08A9.9	1.30	2.46	1.3E-03
F31D4.9.1	1.30	2.46	2.7E-03
W04B5.2	1.30	2.46	2.0E-03
C41H7.9	1.30	2.46	1.7E-03
R03H10.1	1.30	2.45	4.8E-03
T07D3.1	1.29	2.45	1.3E-03
Y66D12A.6	1.29	2.45	2.1E-03
K02E7.11	1.29	2.45	1.3E-03
C26F1.9.1	1.29	2.45	1.4E-03
M04D8.3	1.29	2.45	2.2E-03
Y60A3A.16	1.29	2.45	1.6E-03
C32F10.6	1.29	2.45	1.3E-03
F37A4.2	1.29	2.45	1.9E-03
R12C12.6b	1.29	2.45	1.5E-03
F26F2.8	1.29	2.45	1.5E-03
C29F7.4	1.29	2.45	1.3E-03
K07B1.7b	1.29	2.44	1.5E-03
F07B7.2	1.29	2.44	3.5E-03
F46F2.5	1.29	2.44	1.3E-03
K10D2.2.1	1.29	2.44	1.4E-03
Y39G10AR.20.1	1.29	2.44	1.9E-03
T23F11.4	1.29	2.44	1.4E-03
F17E9.12	1.29	2.44	2.2E-03
T08D10.4	1.29	2.44	1.7E-03
F15D4.2.1	1.29	2.44	2.2E-03
F08H9.5	1.29	2.44	1.7E-03
D1086.1	1.29	2.44	3.9E-03
F14D2.14	1.28	2.44	2.6E-03
ZK512.4	1.28	2.43	4.2E-03
F25H9.4	1.28	2.43	1.4E-03
C15H11.8	1.28	2.43	1.8E-03
ZC395.7	1.28	2.43	1.6E-03
ZC376.4	1.28	2.43	1.4E-03
Y43F4B.9.1	1.28	2.43	1.3E-03
K02F2.4	1.28	2.43	1.6E-03
Y57A10A.30b.1	1.28	2.43	2.5E-03
F23D12.7	1.28	2.43	3.6E-03
ZK697.14	1.28	2.43	1.6E-03
T28H11.2	1.28	2.43	1.7E-03
F26A3.7	1.28	2.43	1.7E-03
F08H9.8	1.28	2.43	1.7E-03
Y108G3AL.6.2	1.28	2.42	1.6E-03

Supporting Table 2: Significant changes after 48h in H_2S			
gene	logFC (48hr)	fold-change	adj.P.Val (48hr)
C27C7.7	1.28	2.42	4.5E-03
T26C12.3	1.28	2.42	2.1E-03
F36D3.3	1.28	2.42	1.6E-03
T10G3.6	1.28	2.42	1.9E-03
ZC302.3	1.28	2.42	2.2E-03
Y92H12A.3	1.28	2.42	3.6E-03
Y7A9D.1	1.28	2.42	2.2E-03
D1014.2	1.28	2.42	1.6E-03
K10B3.1b	1.28	2.42	1.4E-03
E04A4.7.2	1.27	2.42	2.7E-03
C24B9.9	1.27	2.42	1.4E-03
T03D8.7	1.27	2.42	1.9E-03
M176.3.1	1.27	2.42	1.7E-03
F25E5.2	1.27	2.41	1.7E-03
Y111B2A.10b.3	1.27	2.41	1.5E-03
C43E11.13	1.27	2.41	4.2E-03
T23B5.3a	1.27	2.41	1.9E-03
F14D2.5	1.27	2.41	1.4E-03
ZK512.11	1.27	2.41	2.0E-03
F49F1.7	1.27	2.41	2.4E-03
K08D10.12.1	1.27	2.41	1.9E-03
Y66D12A.22.1	1.27	2.41	1.9E-03
C07G3.3	1.27	2.41	2.3E-03
Y46G5A.35	1.27	2.41	1.6E-03
Y48G9A.8.1	1.27	2.41	1.6E-03
F45G2.9	1.27	2.41	1.4E-03
F37B1.2	1.27	2.41	2.1E-03
Y59A8A.3.2	1.27	2.40	6.3E-03
F44A2.7	1.26	2.40	1.8E-03
Y18D10A.18	1.26	2.40	1.4E-03
C07H6.2	1.26	2.40	3.4E-03
F36F12.4	1.26	2.40	1.5E-03
F55C9.5	1.26	2.40	1.7E-03
Y66D12A.22.2	1.26	2.40	1.9E-03
Y46G5A.18	1.26	2.40	2.8E-03
F22E5.21	1.26	2.40	1.5E-03
F48D6.4a	1.26	2.40	4.5E-03
Y55F3AM.8	1.26	2.40	1.6E-03
F12E12.11	1.26	2.40	5.3E-03
K04C1.3.1	1.26	2.40	2.2E-03
Y55F3AM.11	1.26	2.40	1.5E-03
C13G5.1	1.26	2.40	1.6E-03
C05B10.1	1.26	2.40	1.9E-03
Y48A6B.4	1.26	2.39	1.6E-03
C33C12.9	1.26	2.39	1.8E-03
Y102A11A.8	1.26	2.39	3.0E-03
Y51H7C.7	1.26	2.39	1.6E-03

Supporting Table 2: Significant changes after 48h in H_2S

gene	logFC (48hr)	fold-change	adj.P.Val (48hr)
T03E6.5	1.26	2.39	2.3E-03
D2063.3a	1.26	2.39	1.4E-03
R02D3.8	1.26	2.39	1.6E-03
Y56A3A.28	1.26	2.39	2.3E-03
F54F7.1	1.26	2.39	1.7E-03
W09G10.5	1.25	2.38	1.5E-03
ZC204.3	1.25	2.38	1.7E-03
T22H2.4	1.25	2.38	5.7E-03
F47H4.11	1.25	2.38	1.8E-03
F14B8.2	1.25	2.38	5.3E-03
C34B7.1	1.25	2.38	1.7E-03
Y23H5B.8	1.25	2.38	1.5E-03
Y37E3.3	1.25	2.38	4.9E-03
F33H2.3.2	1.25	2.38	1.9E-03
F59E11.7b	1.25	2.38	2.0E-03
Y48G8AL.14	1.25	2.37	1.9E-03
T06D4.1	1.25	2.37	1.6E-03
ZK970.3	1.24	2.37	2.8E-03
F33E11.1	1.24	2.37	1.8E-03
F22B3.1	1.24	2.37	1.8E-03
C50E3.11	1.24	2.37	2.3E-03
F57B10.14.1	1.24	2.37	2.1E-03
Y51F10.11	1.24	2.37	1.7E-03
ZC373.2	1.24	2.36	1.9E-03
K11H3.5	1.24	2.36	2.1E-03
Y40A1A.3	1.24	2.36	1.6E-03
F40F4.3	1.24	2.36	1.9E-03
R07E5.7.1	1.24	2.36	1.6E-03
C01H6.5a	1.24	2.36	2.5E-03
C54E10.6	1.24	2.36	2.0E-03
H06H21.11	1.24	2.36	2.3E-03
Y48E1B.8	1.24	2.36	1.6E-03
F47H4.2	1.24	2.36	2.3E-03
R05H11.2	1.24	2.36	1.7E-03
R11E3.2	1.24	2.35	1.7E-03
F53H2.1	1.24	2.35	2.6E-03
Y54F10BM.12	1.23	2.35	2.1E-03
F54D8.1.1	1.23	2.35	1.6E-03
F28C6.10	1.23	2.35	1.7E-03
W06D11.3	1.23	2.35	2.4E-03
F40C5.3	1.23	2.35	1.7E-03
T09A12.2b	1.23	2.35	2.7E-03
C24F3.2	1.23	2.35	2.6E-03
Y71F9AL.12	1.23	2.35	3.2E-03
Y57G11C.8	1.23	2.35	2.5E-03
K07B1.8	1.23	2.35	1.7E-03
Y102A5C.1	1.23	2.35	3.0E-03

Supporting Table 2: Significant changes after 48h in H_2S			
gene	logFC (48hr)	fold-change	adj.P.Val (48hr)
C30F12.3	1.23	2.35	1.7E-03
F22E5.17	1.23	2.34	1.7E-03
F28B4.1	1.23	2.34	1.8E-03
F32H2.10	1.23	2.34	1.9E-03
F25H9.2	1.23	2.34	9.6E-03
R07C3.11	1.23	2.34	2.4E-03
F58A4.6	1.23	2.34	2.3E-03
F29G9.1	1.23	2.34	2.5E-03
Y22D7AR.11	1.23	2.34	5.2E-03
F29C12.5	1.23	2.34	2.2E-03
Y111B2A.28	1.22	2.34	2.6E-03
F22A3.7	1.22	2.34	7.4E-03
B0024.12	1.22	2.34	2.3E-03
C36B1.6	1.22	2.33	1.8E-03
Y38E10A.24.1	1.22	2.33	2.2E-03
W09G12.9	1.22	2.33	1.8E-03
T05B4.1	1.22	2.33	7.2E-03
Y79H2A.2	1.22	2.33	4.7E-03
C05B5.8	1.22	2.33	2.2E-03
F59C6.12	1.22	2.33	2.5E-03
F18E3.11	1.22	2.33	2.5E-03
Y92C3B.3b	1.22	2.33	2.4E-03
K03B4.7b	1.22	2.33	2.5E-03
C06C3.7	1.22	2.33	3.7E-03
C53B7.3	1.22	2.33	2.2E-03
F43G9.13.1	1.22	2.33	3.3E-03
F55G7.2	1.22	2.32	4.9E-03
T23G11.2.2	1.22	2.32	2.2E-03
K11B4.2	1.22	2.32	5.5E-03
F48C1.2	1.22	2.32	2.2E-03
D1054.1	1.22	2.32	2.4E-03
F44E7.9	1.22	2.32	2.3E-03
Y44A6B.3	1.22	2.32	2.0E-03
ZK1127.12	1.22	2.32	2.1E-03
C01H6.5b.2	1.21	2.32	2.3E-03
Y75D11A.3	1.21	2.32	2.1E-03
Y54G2A.26a.2	1.21	2.32	4.5E-03
Y52B11A.2b	1.21	2.32	2.8E-03
Y37E3.13	1.21	2.32	2.0E-03
Y66A7A.2	1.21	2.32	1.9E-03
K09C4.1a	1.21	2.32	5.2E-03
F26H9.4	1.21	2.32	3.4E-03
C41C4.1	1.21	2.32	4.5E-03
C08F11.7	1.21	2.32	2.1E-03
T11F8.1	1.21	2.31	2.5E-03
M05B5.5a.1	1.21	2.31	2.7E-03
T12D8.10.1	1.21	2.31	3.9E-03

Supporting Table 2: Significant changes after 48h in H_2S			
gene	logFC (48hr)	fold-change	adj.P.Val (48hr)
C34D4.10	1.21	2.31	2.7E-03
Y102A5C.36	1.21	2.31	2.2E-03
T19B4.5	1.21	2.31	2.0E-03
F21D9.1	1.21	2.31	3.8E-03
T24D8.5	1.21	2.31	2.8E-03
W09B6.4b	1.21	2.31	2.0E-03
Y58A7A.1	1.21	2.31	2.2E-03
Y59A8B.7	1.21	2.31	2.8E-03
C27A2.2b	1.21	2.31	3.1E-03
F08G12.5	1.20	2.31	5.7E-03
K08F11.1	1.20	2.30	2.9E-03
F17A9.6	1.20	2.30	2.1E-03
T02G5.2	1.20	2.30	7.3E-03
C52D10.8	1.20	2.30	2.2E-03
C24H10.4	1.20	2.30	2.1E-03
C42C1.13	1.20	2.30	3.3E-03
F33H2.8	1.20	2.30	3.5E-03
C48B4.6	1.20	2.30	2.4E-03
T20B3.15	1.20	2.30	2.5E-03
C44E12.1	1.20	2.29	3.2E-03
Y57A10A.3	1.20	2.29	2.1E-03
Y59A8B.22.1	1.20	2.29	4.5E-03
Y37D8A.19	1.20	2.29	2.2E-03
T23B12.7	1.20	2.29	3.5E-03
T27F2.3.1	1.20	2.29	2.5E-03
F02H6.3a	1.20	2.29	3.0E-03
B0454.9	1.19	2.29	2.5E-03
ZK593.7.1	1.19	2.28	3.0E-03
F08F1.5	1.19	2.28	2.5E-03
F31F6.2	1.19	2.28	4.1E-03
K03H1.6	1.19	2.28	3.9E-03
Y39G10AL.3.2	1.19	2.28	3.3E-03
Y116A8A.9.1	1.19	2.28	2.6E-03
3R5.1	1.19	2.28	2.2E-03
Y24F12A.4	1.19	2.28	2.6E-03
T24E12.9	1.19	2.28	3.3E-03
Y77E11A.7a	1.19	2.28	2.3E-03
Y41D4B.12b	1.19	2.28	3.6E-03
Y39A3CR.7	1.19	2.27	2.8E-03
F42H10.6.2	1.19	2.27	3.3E-03
F26E4.2	1.19	2.27	3.3E-03
F44E2.7b.3	1.18	2.27	2.2E-03
F39H2.1.1	1.18	2.27	4.9E-03
F54D11.3	1.18	2.27	2.3E-03
T20G5.9	1.18	2.27	2.7E-03
C08E3.3	1.18	2.27	9.0E-03
F52C12.5.1	1.18	2.27	3.5E-03

Supporting Table 2: Significant changes after 48h in H_2S			
gene	logFC (48hr)	fold-change	adj.P.Val (48hr)
ZC53.6	1.18	2.27	3.0E-03
Y113G7B.4	1.18	2.27	3.6E-03
ZC132.4	1.18	2.27	4.5E-03
F22D6.8	1.18	2.27	2.3E-03
C44C1.3	1.18	2.27	5.4E-03
T16G1.10a	1.18	2.27	3.3E-03
T24C4.3	1.18	2.27	2.5E-03
C54G4.6	1.18	2.27	3.1E-03
W01C9.1	1.18	2.27	4.3E-03
C16C10.4	1.18	2.27	3.6E-03
JC8.8.1	1.18	2.26	3.5E-03
Y73C8C.2	1.18	2.26	6.6E-03
ZK131.1	1.18	2.26	4.1E-03
F14H12.6	1.18	2.26	2.5E-03
T20H9.2	1.18	2.26	4.8E-03
F45H11.5	1.18	2.26	2.3E-03
T04H1.2.1	1.18	2.26	4.2E-03
T26A5.8	1.18	2.26	3.4E-03
Y42A5A.5	1.18	2.26	4.7E-03
C34G6.1	1.18	2.26	9.2E-03
C29F9.2	1.18	2.26	2.4E-03
H14E04.3	1.17	2.26	3.0E-03
Y48G1A.2	1.17	2.26	3.0E-03
C06G3.11b.2	1.17	2.26	3.7E-03
K10B3.1a	1.17	2.26	4.3E-03
B0513.3.1	1.17	2.25	3.1E-03
F22B3.2	1.17	2.25	2.7E-03
E02H1.8	1.17	2.25	8.0E-03
E01B7.2	1.17	2.25	2.9E-03
C09G9.7	1.17	2.25	4.3E-03
Y32H12A.6	1.17	2.25	2.5E-03
T10E9.8	1.17	2.25	5.1E-03
Y67D2.5	1.17	2.25	3.4E-03
Y62E10A.20.2	1.17	2.25	3.9E-03
M02A10.1	1.17	2.25	2.5E-03
Y57A10A.32	1.17	2.25	4.8E-03
Y73F4A.2	1.17	2.25	4.9E-03
F28D1.5	1.17	2.25	3.3E-03
Y106G6H.14	1.17	2.25	3.2E-03
Y57G11C.3a	1.17	2.25	2.6E-03
C32H11.7a	1.17	2.25	4.7E-03
Y56A3A.19	1.17	2.24	2.6E-03
F21D5.8	1.17	2.24	2.9E-03
W10G11.20	1.16	2.24	3.7E-03
C16A3.2	1.16	2.24	2.9E-03
H39E20.1	1.16	2.24	3.2E-03
C14C10.6	1.16	2.24	3.1E-03

Supporting Table 2: Significant changes after 48h in H_2S			
gene	logFC (48hr)	fold-change	adj.P.Val (48hr)
Y116A8C.17	1.16	2.24	8.0E-03
C33H5.19.2	1.16	2.24	3.3E-03
F19B10.1	1.16	2.24	4.0E-03
C09G9.8	1.16	2.24	3.3E-03
C06A5.11	1.16	2.24	3.7E-03
C06B3.3	1.16	2.24	5.3E-03
Y41C4A.16	1.16	2.24	6.4E-03
Y39B6A.2	1.16	2.24	4.9E-03
Y41E3.8	1.16	2.24	7.2E-03
K06C4.12	1.16	2.24	5.1E-03
Y71G12B.21	1.16	2.24	2.6E-03
T07C12.9	1.16	2.24	4.1E-03
Y47H9C.11	1.16	2.24	3.3E-03
Y116F11A.1	1.16	2.24	1.4E-02
K08A2.4	1.16	2.23	2.9E-03
F29G9.3	1.16	2.23	4.7E-03
F54B11.6	1.16	2.23	2.6E-03
F38H4.10	1.16	2.23	3.4E-03
Y39C12A.9	1.16	2.23	1.0E-02
ZK637.9a	1.16	2.23	2.6E-03
Y102A5C.2	1.16	2.23	3.0E-03
B0513.4	1.16	2.23	2.8E-03
ZK546.11	1.16	2.23	2.5E-02
Y38H8A.5	1.15	2.23	4.2E-03
Y119D3B.19	1.15	2.23	3.4E-03
K03H1.7	1.15	2.22	4.0E-03
Y57A10A.29	1.15	2.22	4.3E-03
F57C7.3b	1.15	2.22	3.5E-03
T09A5.15	1.15	2.22	3.6E-03
D2030.7.1	1.15	2.22	3.3E-03
M05D6.6.2	1.15	2.22	3.7E-03
Y59A8B.12	1.15	2.22	3.0E-03
Y102A11A.6	1.15	2.22	3.8E-03
T09A5.4	1.15	2.22	3.8E-03
T10F2.2	1.15	2.21	3.3E-03
C02B8.2	1.15	2.21	3.0E-03
K02A2.1	1.15	2.21	5.3E-03
Y54G11A.10	1.15	2.21	2.8E-03
C40A11.8	1.15	2.21	4.1E-03
T28H11.4	1.15	2.21	3.1E-03
K08F4.7	1.15	2.21	6.3E-03
C40H1.5	1.15	2.21	5.8E-03
Y54F10AM.11	1.14	2.21	3.2E-03
Y46H3C.5	1.14	2.21	3.3E-03
T04A11.2	1.14	2.21	1.0E-02
ZK643.2	1.14	2.21	4.7E-03
R11A8.1	1.14	2.21	3.5E-03

Supporting Table 2: Significant changes after 48h in H_2S			
gene	logFC (48hr)	fold-change	adj.P.Val (48hr)
F32H5.3b	1.14	2.21	7.7E-03
F36H5.10	1.14	2.21	6.6E-03
Y54G2A.26a.1	1.14	2.21	3.0E-03
F54F2.7	1.14	2.20	3.6E-03
C17G1.2	1.14	2.20	4.2E-03
C10F3.5	1.14	2.20	3.2E-03
T05A7.1	1.14	2.20	8.4E-03
C46F4.1	1.14	2.20	4.7E-03
M7.12	1.14	2.20	1.0E-02
B0410.2b	1.14	2.20	4.7E-03
Y11D7A.10.1	1.14	2.20	3.6E-03
F52D2.2	1.14	2.20	4.6E-03
R144.9	1.14	2.20	3.2E-03
Y27F2A.3a	1.14	2.20	3.6E-03
Y37D8A.18	1.14	2.20	4.1E-03
F29G9.7	1.14	2.20	3.4E-03
H30A04.1a	1.14	2.20	4.1E-03
F42F12.10	1.14	2.20	3.1E-03
T20B3.13	1.14	2.20	3.9E-03
D1054.3.1	1.13	2.19	3.5E-03
W03C9.5	1.13	2.19	3.5E-03
F56C3.9	1.13	2.19	7.2E-03
Y48A5A.3	1.13	2.19	3.9E-03
F08F1.9	1.13	2.19	4.1E-03
Y49E10.15	1.13	2.19	4.7E-03
R52.9	1.13	2.19	5.4E-03
ZK662.5	1.13	2.19	4.6E-03
F02E8.3	1.13	2.19	3.6E-03
F58D5.9	1.13	2.19	3.8E-03
C47B2.1	1.13	2.19	6.2E-03
T03F1.2	1.13	2.19	4.5E-03
C09E9.1	1.13	2.19	3.6E-03
R05D3.6	1.13	2.19	4.9E-03
H17B01.3	1.13	2.19	1.1E-02
C27H6.4b	1.13	2.19	3.3E-03
W02D9.7	1.13	2.19	4.1E-03
T20F5.2	1.13	2.18	6.6E-03
F08B6.2.2	1.13	2.18	1.1E-02
B0304.2	1.13	2.18	4.2E-03
B0365.5b	1.13	2.18	5.5E-03
C16C10.10	1.13	2.18	4.0E-03
T10C6.12	1.13	2.18	5.1E-03
C49H3.4	1.13	2.18	4.8E-03
Y69A2AR.21	1.13	2.18	4.6E-03
B0212.3	1.13	2.18	3.6E-03
F44E2.6	1.13	2.18	6.9E-03
K08H10.1.1	1.13	2.18	6.1E-03

Supporting Table 2: Significant changes after 48h in H_2S			
gene	logFC (48hr)	fold-change	adj.P.Val (48hr)
C16C4.14	1.13	2.18	8.3E-03
R01E6.7	1.12	2.18	3.9E-03
Y37E11AM.2.1	1.12	2.18	3.3E-03
K10H10.3a	1.12	2.18	3.4E-03
F28D1.11	1.12	2.18	3.9E-03
F54D10.7	1.12	2.18	5.8E-03
R119.2	1.12	2.18	4.3E-03
Y54G9A.5	1.12	2.18	3.3E-03
F42H10.6.1	1.12	2.17	5.4E-03
C07G3.6	1.12	2.17	4.9E-03
T25G3.1	1.12	2.17	5.4E-03
F08G2.3	1.12	2.17	1.0E-02
Y41D4A.5	1.12	2.17	4.6E-03
C23G10.11	1.12	2.17	7.6E-03
F54D10.5	1.12	2.17	5.0E-03
Y110A2AL.2	1.12	2.17	3.8E-03
ZK829.6	1.12	2.17	4.1E-03
F35G12.9	1.12	2.17	5.5E-03
C06A5.8a	1.12	2.17	3.8E-03
F10E7.6	1.12	2.17	4.3E-03
F13H6.4	1.12	2.17	4.1E-03
M116.2	1.12	2.17	5.1E-03
R09B3.3.1	1.12	2.17	7.7E-03
F13G3.9	1.12	2.17	5.4E-03
R09H3.3	1.12	2.17	6.3E-03
W09H1.6a	1.12	2.17	3.5E-03
Y52B11A.8	1.11	2.17	1.1E-02
Y48G8AR.2	1.11	2.16	4.7E-03
R07B1.2.1	1.11	2.16	5.1E-03
C54G6.1a	1.11	2.16	3.7E-03
F31E3.6	1.11	2.16	5.4E-03
C16C8.12	1.11	2.16	5.7E-03
T08E11.1	1.11	2.16	8.3E-03
Y37E11B.3	1.11	2.16	5.6E-03
C25G6.5	1.11	2.16	3.9E-03
EGAP2.1	1.11	2.16	6.2E-03
C29G2.1	1.11	2.16	5.0E-03
F21H7.12	1.11	2.16	7.0E-03
C33H5.19.1	1.11	2.16	4.9E-03
F49B2.1	1.11	2.16	5.5E-03
C36B1.14	1.11	2.15	5.9E-03
ZK742.5	1.11	2.15	3.8E-03
C55A6.5	1.11	2.15	5.8E-03
C18B12.5	1.11	2.15	4.1E-03
Y82E9BL.11	1.11	2.15	4.1E-03
M03E7.5.1	1.10	2.15	4.1E-03
C48D1.2	1.10	2.15	4.6E-03

Supporting Table 2: Significant changes after 48h in H_2S			
gene	logFC (48hr)	fold-change	adj.P.Val (48hr)
F40F8.3	1.10	2.15	4.1E-03
F37B4.10	1.10	2.15	3.9E-03
Y54E10BR.2	1.10	2.15	3.7E-03
Y73E7A.6	1.10	2.15	4.6E-03
T27E4.3	1.10	2.15	1.8E-02
F26A3.2	1.10	2.14	4.6E-03
F59G1.8	1.10	2.14	1.2E-02
Y38F2AL.2	1.10	2.14	4.9E-03
F01D4.1	1.10	2.14	7.4E-03
F37C12.14	1.10	2.14	4.8E-03
F46G11.4	1.10	2.14	5.2E-03
Y71F9AL.14.1	1.10	2.14	3.8E-03
F25H5.6.2	1.10	2.14	1.1E-02
ZK6.3	1.10	2.14	4.8E-03
R03G8.4	1.10	2.14	8.6E-03
C35E7.5b	1.10	2.14	1.9E-02
K07A1.6	1.10	2.14	4.8E-03
Y69A2AR.5	1.10	2.14	7.2E-03
F21D5.4	1.10	2.14	6.7E-03
ZK131.2	1.10	2.14	5.1E-03
W02A11.8	1.10	2.14	4.9E-03
C46C2.6b	1.09	2.14	6.1E-03
H31G24.3	1.09	2.14	4.0E-03
CD4.7	1.09	2.13	5.0E-03
F53A2.3	1.09	2.13	3.9E-03
F58H1.3a	1.09	2.13	4.2E-03
T01C3.2	1.09	2.13	8.7E-03
C44B7.1.1	1.09	2.13	3.9E-03
C18D4.6a	1.09	2.13	4.8E-03
T09F3.4	1.09	2.13	5.2E-03
F56C11.3	1.09	2.13	5.5E-03
Y71G12B.14	1.09	2.13	7.9E-03
Y55F3BR.11	1.09	2.13	6.8E-03
F09C8.2.1	1.09	2.13	9.3E-03
M01F1.8b	1.09	2.13	4.4E-03
C41D11.9	1.09	2.13	4.5E-03
M01A12.3	1.09	2.13	4.5E-03
T23B12.1	1.09	2.13	6.5E-03
F57C2.3	1.09	2.13	4.3E-03
C06G3.11b.1	1.09	2.13	1.1E-02
K11H12.6	1.09	2.13	4.4E-03
ZK632.8	1.09	2.13	5.0E-03
D2013.3	1.09	2.13	8.8E-03
C45G7.4	1.09	2.13	7.0E-03
E04F6.8.1	1.09	2.13	9.9E-03
Y75D11A.1	1.09	2.13	5.1E-03
C24H11.6	1.09	2.12	5.5E-03

Supporting Table 2: Significant changes after 48h in H_2S			
gene	logFC (48hr)	fold-change	adj.P.Val (48hr)
F22B5.1	1.09	2.12	6.5E-03
C45G3.3	1.09	2.12	5.1E-03
C05C10.7	1.09	2.12	7.2E-03
C14C6.5	1.09	2.12	4.3E-03
B0035.9	1.09	2.12	6.0E-03
C07G1.8	1.09	2.12	5.6E-03
Y116A8C.15	1.09	2.12	6.2E-03
C25E10.9a	1.09	2.12	5.6E-03
Y71H2AM.24	1.08	2.12	4.8E-03
Y65B4A.4	1.08	2.12	4.5E-03
F10E9.7	1.08	2.12	6.0E-03
F28D9.4	1.08	2.12	5.1E-03
Y63D3A.2	1.08	2.12	6.4E-03
Y59A8A.3.1	1.08	2.12	1.2E-02
ZK1128.8b	1.08	2.12	5.8E-03
ZC376.1	1.08	2.12	7.6E-03
F57B10.12	1.08	2.12	4.8E-03
F13G3.10	1.08	2.12	4.4E-03
K09C4.6	1.08	2.12	7.1E-03
W02D7.11	1.08	2.12	4.5E-03
R10H10.1.1	1.08	2.12	8.4E-03
K02E2.6.1	1.08	2.12	6.2E-03
Y73B3A.1	1.08	2.12	4.9E-03
W03G9.3	1.08	2.11	5.8E-03
E02C12.4	1.08	2.11	6.8E-03
F55H2.5	1.08	2.11	8.6E-03
C36B1.3	1.08	2.11	4.7E-03
Y116A8C.42	1.08	2.11	8.1E-03
F54E12.1	1.08	2.11	6.3E-03
F07F6.7	1.08	2.11	4.3E-03
ZK849.6	1.08	2.11	7.2E-03
R07C3.2	1.08	2.11	6.7E-03
T27E4.4	1.08	2.11	4.7E-03
Y67D8A.3	1.08	2.11	5.3E-03
F53A3.1	1.08	2.11	4.4E-03
F28F8.5	1.08	2.11	6.8E-03
ZK678.3	1.08	2.11	5.4E-03
F46F3.1	1.08	2.11	4.4E-03
F15B9.3	1.08	2.11	5.4E-03
W02D9.5	1.08	2.11	5.7E-03
Y11D7A.7	1.07	2.11	4.4E-03
M03F8.2b	1.07	2.11	9.0E-03
T27F7.1.2	1.07	2.11	6.2E-03
T27F6.2	1.07	2.11	4.8E-03
F36F2.4	1.07	2.10	5.9E-03
T07A9.13a	1.07	2.10	1.2E-02
C30G12.1	1.07	2.10	6.9E-03

Supporting Table 2: Significant changes after 48h in H_2S			
gene	logFC (48hr)	fold-change	adj.P.Val (48hr)
Y50E8A.17	1.07	2.10	4.8E-03
C18A11.5b	1.07	2.10	8.7E-03
K05C4.9	1.07	2.10	1.2E-02
ZC416.2	1.07	2.10	4.5E-03
F48G7.2	1.07	2.10	5.5E-03
F34D10.3	1.07	2.10	6.8E-03
K11H12.11	1.07	2.10	7.0E-03
F59A7.1	1.07	2.10	6.6E-03
Y11D7A.10.2	1.07	2.10	4.9E-03
W02H3.1	1.07	2.10	6.5E-03
F22F1.2	1.07	2.09	4.9E-03
F53F8.6	1.07	2.09	7.6E-03
C06G3.11a	1.07	2.09	1.5E-02
C26C6.4b	1.07	2.09	6.6E-03
Y49A3A.1	1.07	2.09	4.9E-03
C29E4.12	1.06	2.09	6.7E-03
C06E2.5.2	1.06	2.09	6.3E-03
B0047.1a	1.06	2.09	4.7E-03
Y39G10AR.10.2	1.06	2.09	5.9E-03
M01D1.9	1.06	2.09	5.7E-03
C47C12.3a.1	1.06	2.09	1.6E-02
F49C5.4	1.06	2.09	4.8E-03
F16B4.8	1.06	2.09	6.3E-03
R10D12.13b	1.06	2.09	4.9E-03
B0416.7a	1.06	2.09	5.1E-03
R10D12.14a	1.06	2.09	5.0E-03
C36A4.1	1.06	2.09	1.2E-02
B0564.2	1.06	2.09	8.9E-03
F31E8.4	1.06	2.09	5.4E-03
ZK1055.2	1.06	2.09	7.2E-03
F54C4.4	1.06	2.09	5.1E-03
Y62E10A.12.1	1.06	2.09	5.1E-03
Y73B6BL.14	1.06	2.08	5.0E-03
T25E12.13	1.06	2.08	9.9E-03
T22C1.5	1.06	2.08	7.6E-03
Y6B3B.5a	1.06	2.08	5.1E-03
F56D12.5a.2	1.06	2.08	5.1E-03
K05C4.4	1.06	2.08	7.2E-03
R12B2.2	1.06	2.08	6.4E-03
Y110A2AR.2	1.06	2.08	5.2E-03
T05E7.1	1.06	2.08	1.1E-02
EEED8.14	1.06	2.08	8.5E-03
R04B5.4	1.06	2.08	5.1E-03
F33D4.7	1.06	2.08	1.2E-02
F25H5.8	1.06	2.08	5.5E-03
C03B1.5	1.06	2.08	5.8E-03
C06E1.1	1.05	2.08	7.1E-03

Supporting Table 2: Significant changes after 48h in H_2S			
gene	logFC (48hr)	fold-change	adj.P.Val (48hr)
C34B7.3	1.05	2.08	6.2E-03
H06I04.7	1.05	2.08	6.5E-03
Y39B6A.11	1.05	2.08	5.4E-03
ZC239.13	1.05	2.07	6.6E-03
K04A8.4	1.05	2.07	8.7E-03
C35B1.4	1.05	2.07	8.2E-03
B0041.6a	1.05	2.07	5.1E-03
ZK652.1.1	1.05	2.07	8.4E-03
F13H8.8	1.05	2.07	5.6E-03
W08A12.1b	1.05	2.07	9.7E-03
ZC239.16	1.05	2.07	8.4E-03
EEED8.3	1.05	2.07	6.7E-03
B0205.12	1.05	2.07	6.4E-03
B0025.4	1.05	2.07	5.8E-03
Y111B2A.12	1.05	2.07	5.2E-03
C04G6.5	1.05	2.07	7.1E-03
Y17D7B.2	1.05	2.07	5.2E-03
C52B9.3b	1.05	2.07	5.6E-03
F38E9.6	1.05	2.07	5.7E-03
F23C8.9	1.05	2.07	7.4E-03
Y43B11AR.1	1.05	2.06	5.8E-03
Y54H5A.3	1.05	2.06	7.0E-03
F59A2.5	1.04	2.06	1.2E-02
F57A8.8	1.04	2.06	8.7E-03
Y37E11AM.2.2	1.04	2.06	5.3E-03
F40G9.2	1.04	2.06	7.2E-03
C16A11.3	1.04	2.06	6.5E-03
F56D2.3	1.04	2.06	6.9E-03
F52F12.6	1.04	2.06	7.5E-03
F17E9.10	1.04	2.06	7.9E-03
F08B4.7.1	1.04	2.06	5.6E-03
F52A8.5.1	1.04	2.06	8.1E-03
T01E8.2	1.04	2.06	5.4E-03
F25B4.4	1.04	2.06	8.3E-03
C10A4.8	1.04	2.06	5.5E-03
F54E12.4	1.04	2.06	1.6E-02
F40F8.9.2	1.04	2.06	6.2E-03
F32D1.2.2	1.04	2.05	7.4E-03
F30F8.7	1.04	2.05	6.2E-03
T25G12.8	1.04	2.05	6.6E-03
Y47D3A.32	1.04	2.05	8.6E-03
C24A1.2a	1.04	2.05	7.1E-03
M163.8	1.04	2.05	5.6E-03
F07B7.5	1.04	2.05	5.8E-03
F25E5.13	1.04	2.05	6.0E-03
C01F1.6	1.04	2.05	5.6E-03
F53A3.7	1.04	2.05	8.1E-03

Supporting Table 2: Significant changes after 48h in H_2S			
gene	logFC (48hr)	fold-change	adj.P.Val (48hr)
C45G9.11	1.04	2.05	6.2E-03
W02D3.12	1.04	2.05	9.2E-03
C34B2.9	1.04	2.05	5.5E-03
C43H8.1.1	1.03	2.05	5.9E-03
C27B7.2	1.03	2.05	6.6E-03
F11E6.11.1	1.03	2.05	6.8E-03
Y54F10BM.13	1.03	2.05	8.4E-03
Y45F10A.3	1.03	2.05	8.5E-03
K09D9.12	1.03	2.05	5.9E-03
C36B1.7	1.03	2.05	8.4E-03
T22F7.5	1.03	2.05	1.1E-02
K09F6.2	1.03	2.05	6.3E-03
R12C12.5	1.03	2.05	6.5E-03
F25H9.6	1.03	2.05	6.7E-03
F14D2.1	1.03	2.05	6.2E-03
ZK652.2	1.03	2.04	7.7E-03
F15D4.2.2	1.03	2.04	6.8E-03
M02B7.7	1.03	2.04	8.2E-03
K07A1.12.1	1.03	2.04	5.8E-03
C44B9.6	1.03	2.04	9.5E-03
F32D1.2.1	1.03	2.04	7.1E-03
F55G1.10	1.03	2.04	1.0E-02
C25G4.3	1.03	2.04	6.7E-03
B0391.5	1.03	2.04	6.6E-03
Y37E3.4.1	1.03	2.04	1.2E-02
K02B7.2	1.03	2.04	6.2E-03
R07C3.9	1.03	2.04	6.0E-03
R03D7.6	1.03	2.04	1.1E-02
Y73B3B.2	1.03	2.04	5.9E-03
C44B7.1.2	1.03	2.04	6.4E-03
T12G3.6	1.03	2.04	9.8E-03
JC8.11a	1.03	2.04	5.9E-03
F09E5.8	1.03	2.04	8.1E-03
R05G6.5	1.03	2.04	5.8E-03
C18D11.4.2	1.03	2.04	5.9E-03
T04D3.5	1.03	2.04	6.9E-03
K06C4.5	1.03	2.04	9.7E-03
Y62H9A.6	1.03	2.04	7.5E-03
Y73C8A.1	1.03	2.04	6.1E-03
T28F2.2	1.03	2.04	6.6E-03
F13E6.3	1.03	2.04	6.7E-03
F55H2.1	1.02	2.03	1.3E-02
Y48G8AL.7	1.02	2.03	7.7E-03
C01G10.12	1.02	2.03	7.6E-03
T19B10.11	1.02	2.03	9.5E-03
R09H10.3a	1.02	2.03	7.8E-03
Y37D8A.12a.1	1.02	2.03	6.2E-03

Supporting Table 2: Significant changes after 48h in H_2S			
gene	logFC (48hr)	fold-change	adj.P.Val (48hr)
K09C6.4	1.02	2.03	1.4E-02
Y41D4B.26	1.02	2.03	7.1E-03
C01A2.5	1.02	2.03	7.0E-03
R06B9.5	1.02	2.03	7.6E-03
Y45G12B.2b	1.02	2.03	1.1E-02
R02E12.2a	1.02	2.03	7.6E-03
W02D9.4	1.02	2.03	7.5E-03
C09B8.5	1.02	2.03	1.0E-02
Y50D4A.5	1.02	2.03	6.9E-03
F39E9.14	1.02	2.03	2.5E-02
T24H7.3.2	1.02	2.03	9.2E-03
F10G8.9a	1.02	2.03	1.7E-02
M03F8.2c	1.02	2.03	1.3E-02
F45C12.2	1.02	2.03	7.7E-03
F26F4.9b	1.02	2.03	8.1E-03
F36H9.4	1.02	2.03	2.0E-02
C26E6.2	1.02	2.03	6.2E-03
C50B8.2	1.02	2.03	6.5E-03
C01G6.7	1.02	2.03	8.8E-03
T14G10.3	1.02	2.03	1.4E-02
Y39A1A.16	1.02	2.02	9.0E-03
F59B1.10	1.02	2.02	7.8E-03
F31B9.3	1.02	2.02	7.6E-03
C34H4.5	1.02	2.02	8.6E-03
F53B7.3	1.02	2.02	6.9E-03
F37H8.3	1.02	2.02	1.3E-02
B0546.4b	1.02	2.02	7.0E-03
F39H2.4	1.02	2.02	8.2E-03
C10A4.6	1.01	2.02	7.2E-03
C32C4.2	1.01	2.02	6.5E-03
F46F2.4	1.01	2.02	6.4E-03
K07A1.10	1.01	2.02	1.3E-02
C04A11.2	1.01	2.02	8.8E-03
C15C7.1.2	1.01	2.02	1.6E-02
F52B11.5	1.01	2.02	8.5E-03
W08F4.11	1.01	2.02	6.6E-03
K12H4.5.1	1.01	2.02	1.1E-02
C16C8.13	1.01	2.02	7.2E-03
F12F6.8	1.01	2.01	1.3E-02
Y119C1B.10	1.01	2.01	6.6E-03
Y53H1A.4	1.01	2.01	9.0E-03
E02H1.5	1.01	2.01	8.0E-03
T08B2.8	1.01	2.01	8.8E-03
T09A5.7.1	1.01	2.01	8.3E-03
F49C12.14	1.01	2.01	9.1E-03
M110.5a.2	1.01	2.01	1.0E-02
F29G9.6a	1.01	2.01	8.7E-03

Supporting Table 2: Significant changes after 48h in H_2S			
gene	logFC (48hr)	fold-change	adj.P.Val (48hr)
W06D11.1	1.01	2.01	7.0E-03
C35D10.17	1.01	2.01	1.0E-02
M110.5c	1.01	2.01	8.8E-03
F37H8.1	1.01	2.01	8.2E-03
F17E9.4	1.01	2.01	7.4E-03
F39B2.5.1	1.01	2.01	9.2E-03
Y71F9AL.5	1.01	2.01	8.2E-03
ZK673.4	1.00	2.01	8.4E-03
F15A4.12	1.00	2.01	9.6E-03
F17E9.13	1.00	2.01	1.1E-02
T14B1.2	1.00	2.01	8.9E-03
T15B7.15	1.00	2.00	8.3E-03
F40H3.6	1.00	2.00	7.0E-03
E01A2.5	1.00	2.00	8.9E-03
K11H3.6	1.00	2.00	8.0E-03
Y41D4B.1	1.00	2.00	8.0E-03
F02E9.1	1.00	2.00	8.0E-03
ZK1053.4	1.00	2.00	6.9E-03
C44B11.6	1.00	2.00	7.7E-03
C44B7.1.4	1.00	-0.50	7.3E-03
Y111B2A.16	1.00	-0.50	9.3E-03
F56A4.3	1.00	-0.50	9.1E-03
T17A3.2	1.00	-0.50	7.3E-03
F13G3.12	1.00	-0.50	4.5E-02
Y106G6E.6.2	1.00	-0.50	2.2E-02
ZK637.13.1	1.00	-0.50	8.0E-03
C04C11.1a	1.00	-0.50	8.8E-03
K01G5.8a.1	0.99	-0.50	1.4E-02
F27E11.3a	0.99	-0.50	1.2E-02
Y39B6A.5	0.99	-0.50	1.3E-02
C27C7.1	0.99	-0.50	9.9E-03
C54D10.3.1	0.99	-0.50	8.6E-03
C34C6.7	0.99	-0.50	7.2E-03
H12D21.13	0.99	-0.50	1.0E-02
F54A5.3c	0.99	-0.50	8.0E-03
F23D12.1	0.99	-0.50	1.2E-02
C34B2.10.1	0.99	-0.50	2.5E-02
H05L14.2	0.99	-0.50	4.1E-02
F52D10.6	0.99	-0.50	8.2E-03
T18D3.9	0.99	-0.50	2.1E-02
JC8.14	0.99	-0.50	1.3E-02
C46F11.3	0.99	-0.50	2.2E-02
W05H7.1	0.99	-0.50	7.4E-03
Y37E3.5a	0.99	-0.50	7.8E-03
Y71F9AL.8	0.99	-0.50	8.4E-03
T03F6.5	0.99	-0.50	1.0E-02
T04A8.9.1	0.99	-0.50	8.9E-03

Supporting Table 2: Significant changes after 48h in H_2S			
gene	logFC (48hr)	fold-change	adj.P.Val (48hr)
ZC395.2	0.99	-0.50	8.6E-03
T05E11.4	0.99	-0.50	1.1E-02
F31F6.3	0.99	-0.50	1.8E-02
C04F12.5	0.99	-0.50	8.3E-03
Y53F4B.3	0.99	-0.50	1.0E-02
F01D5.5	0.99	-0.50	8.1E-03
F54F2.9	0.99	-0.50	1.2E-02
F52D2.6	0.99	-0.50	8.3E-03
T04A8.9.2	0.99	-0.50	9.2E-03
Y71A12B.12a.1	0.99	-0.50	9.1E-03
R07E4.3	0.99	-0.50	8.3E-03
C49G7.11	0.99	-0.50	9.0E-03
F11A10.2	0.99	-0.50	8.2E-03
Y76A2B.1	0.99	-0.50	8.0E-03
F32E10.6.2	0.99	-0.50	8.0E-03
Y57G11C.9c.2	0.99	-0.50	2.7E-02
R10D12.13c	0.99	-0.50	9.0E-03
Y105E8B.7	0.99	-0.50	8.8E-03
Y73B6BL.32.1	0.99	-0.50	7.7E-03
T07A9.1	0.99	-0.50	7.7E-03
Y65B4BR.8	0.99	-0.50	8.4E-03
Y37E11B.1b	0.99	-0.51	1.2E-02
AH10.4	0.99	-0.51	1.7E-02
T28C6.8	0.99	-0.51	1.1E-02
F55A8.1.1	0.99	-0.51	8.2E-03
Y55F3BR.10	0.98	-0.51	7.9E-03
Y97E10AR.5	0.98	-0.51	9.9E-03
C36B7.7	0.98	-0.51	8.3E-03
B0035.7	0.98	-0.51	1.2E-02
ZK688.11	0.98	-0.51	9.0E-03
W01G7.3	0.98	-0.51	1.1E-02
Y110A7A.11	0.98	-0.51	8.3E-03
F19H6.4	0.98	-0.51	9.9E-03
H18N23.2b	0.98	-0.51	1.6E-02
R10E9.3	0.98	-0.51	1.3E-02
F35G8.2	0.98	-0.51	8.2E-03
K04C2.7	0.98	-0.51	1.0E-02
F47B10.5	0.98	-0.51	1.1E-02
C26E6.11	0.98	-0.51	8.6E-03
T15H9.2	0.98	-0.51	8.4E-03
F42F12.1	0.98	-0.51	8.2E-03
C23H3.5.1	0.98	-0.51	1.5E-02
Y41G9A.9	0.98	-0.51	9.8E-03
ZK287.5.1	0.98	-0.51	1.1E-02
F59A7.4	0.98	-0.51	1.4E-02
Y54G2A.44	0.98	-0.51	7.9E-03
T23G11.7a	0.98	-0.51	1.4E-02

Supporting Table 2: Significant changes after 48h in H_2S			
gene	logFC (48hr)	fold-change	adj.P.Val (48hr)
Y50C1A.2	0.98	-0.51	1.1E-02
R08H2.1	0.98	-0.51	2.3E-02
Y47D7A.12	0.98	-0.51	8.0E-03
Y105C5A.1	0.98	-0.51	1.0E-02
C23F12.4	0.98	-0.51	1.4E-02
T03G11.6	0.98	-0.51	1.3E-02
B0348.5	0.98	-0.51	8.8E-03
T10B11.1	0.98	-0.51	9.7E-03
B0464.7.1	0.98	-0.51	1.0E-02
Y54F10BM.6	0.98	-0.51	1.0E-02
D2023.4.1	0.97	-0.51	1.1E-02
K03H1.11	0.97	-0.51	8.2E-03
R144.3	0.97	-0.51	1.1E-02
Y66A7A.8	0.97	-0.51	8.3E-03
F53F8.1	0.97	-0.51	1.5E-02
Y56A3A.3.1	0.97	-0.51	2.4E-02
C53D5.1b	0.97	-0.51	1.0E-02
ZK1127.1	0.97	-0.51	8.3E-03
F33G12.7	0.97	-0.51	1.3E-02
Y39A1A.13	0.97	-0.51	8.6E-03
F41G3.21	0.97	-0.51	1.4E-02
R08F11.7	0.97	-0.51	1.2E-02
T22B11.4a	0.97	-0.51	1.2E-02
E03H4.10	0.97	-0.51	1.0E-02
Y48A6C.1	0.97	-0.51	1.1E-02
C01A2.2	0.97	-0.51	9.8E-03
C54D10.6	0.97	-0.51	1.3E-02
Y51H4A.5	0.97	-0.51	8.6E-03
R05F9.5	0.97	-0.51	1.3E-02
C03B1.10	0.97	-0.51	1.3E-02
F40E3.2	0.97	-0.51	1.0E-02
R05H5.3	0.97	-0.51	1.0E-02
F10E9.11	0.97	-0.51	1.4E-02
F08G2.2	0.97	-0.51	1.3E-02
F45F2.2	0.97	-0.51	1.4E-02
T19E7.1	0.97	-0.51	9.7E-03
F18A1.3a.1	0.97	-0.51	1.2E-02
C17E4.11	0.97	-0.51	1.1E-02
C02B8.1.1	0.97	-0.51	9.7E-03
Y87G2A.10.2	0.97	-0.51	9.5E-03
F32H5.3a	0.97	-0.51	4.1E-02
C37E2.2a	0.96	-0.51	9.6E-03
F41E7.7	0.96	-0.51	8.7E-03
F45E12.5a	0.96	-0.51	1.0E-02
F23F1.3	0.96	-0.51	1.1E-02
Y23H5A.2	0.96	-0.51	1.5E-02
C32E8.6a	0.96	-0.51	2.1E-02

Supporting Table 2: Significant changes after 48h in H_2S			
gene	logFC (48hr)	fold-change	adj.P.Val (48hr)
Y46G5A.12.1	0.96	-0.51	9.6E-03
F41G3.18	0.96	-0.51	1.1E-02
F47G3.2	0.96	-0.51	1.4E-02
C07A12.5a	0.96	-0.51	9.7E-03
T08B2.4	0.96	-0.51	1.2E-02
Y54F10BM.5	0.96	-0.51	1.3E-02
F15A4.9	0.96	-0.51	9.0E-03
F09C12.6	0.96	-0.51	1.3E-02
Y73F8A.37	0.96	-0.51	9.5E-03
B0412.4.1	0.96	-0.51	1.4E-02
C28G1.4	0.96	-0.51	1.8E-02
F22E5.9	0.96	-0.51	1.6E-02
T01C3.9	0.96	-0.51	9.2E-03
T10D4.10	0.96	-0.51	1.5E-02
Y113G7B.3	0.96	-0.51	1.4E-02
C34D4.13	0.96	-0.51	9.2E-03
K12H6.2	0.96	-0.51	1.1E-02
T10G3.3	0.96	-0.51	2.0E-02
F26A1.1	0.96	-0.51	1.1E-02
F46F2.3	0.96	-0.51	9.8E-03
F26F4.12	0.96	-0.51	1.1E-02
Y37H2A.4	0.96	-0.51	1.1E-02
T22G5.3	0.96	-0.51	1.6E-02
Y56A3A.1	0.96	-0.51	1.3E-02
ZK75.3	0.96	-0.51	1.1E-02
W05F2.5	0.96	-0.51	1.4E-02
Y54G2A.19	0.96	-0.51	1.1E-02
T23G11.4	0.96	-0.51	1.2E-02
F07H5.13	0.96	-0.51	2.6E-02
Y39B6A.6	0.96	-0.51	9.7E-03
C29E4.11	0.96	-0.51	1.4E-02
W10D9.3	0.96	-0.51	9.8E-03
F34D10.8	0.96	-0.51	9.6E-03
Y10G11A.2	0.96	-0.52	1.0E-02
Y71A12B.8	0.96	-0.52	1.0E-02
F35C12.3b.1	0.96	-0.52	1.1E-02
ZK813.1.1	0.96	-0.52	1.0E-02
W03H9.2	0.95	-0.52	1.9E-02
Y116A8C.34.1	0.95	-0.52	1.1E-02
K11D12.2.4	0.95	-0.52	1.2E-02
W05G11.2	0.95	-0.52	9.2E-03
Y48G1BL.1	0.95	-0.52	1.4E-02
F01D5.2	0.95	-0.52	1.4E-02
Y71G12B.15.2	0.95	-0.52	1.0E-02
C25A11.1	0.95	-0.52	1.3E-02
T19B4.4.1	0.95	-0.52	1.6E-02
Y54G2A.17a.1	0.95	-0.52	1.3E-02

Supporting Table 2: Significant changes after 48h in H_2S			
gene	logFC (48hr)	fold-change	adj.P.Val (48hr)
C28H8.1	0.95	-0.52	1.0E-02
K06A4.7	0.95	-0.52	1.3E-02
Y41D4A.4.1	0.95	-0.52	1.1E-02
Y51F10.3	0.95	-0.52	9.5E-03
Y111B2A.15	0.95	-0.52	9.2E-03
F55B11.6	0.95	-0.52	2.0E-02
K10H10.2.1	0.95	-0.52	1.1E-02
T23B12.10	0.95	-0.52	1.6E-02
M151.3	0.95	-0.52	1.3E-02
Y53G8B.2	0.95	-0.52	1.5E-02
C49D10.11	0.95	-0.52	1.2E-02
F44F1.6a	0.95	-0.52	1.4E-02
Y17G9A.5	0.95	-0.52	1.1E-02
C26F1.3	0.95	-0.52	9.6E-03
F23F1.7	0.95	-0.52	1.2E-02
Y71H2AL.2	0.95	-0.52	1.3E-02
Y54E5A.5.1	0.95	-0.52	1.0E-02
F59A1.8	0.95	-0.52	1.2E-02
F39E9.7	0.95	-0.52	1.4E-02
K12H6.11	0.95	-0.52	1.2E-02
F42C5.6	0.94	-0.52	1.1E-02
Y48C3A.8	0.94	-0.52	1.3E-02
F01F1.2	0.94	-0.52	1.8E-02
K07E3.8b	0.94	-0.52	1.4E-02
B0035.4.1	0.94	-0.52	1.3E-02
C54C6.6	0.94	-0.52	2.4E-02
Y48C3A.16	0.94	-0.52	1.1E-02
F43D2.2	0.94	-0.52	1.0E-02
ZK287.3	0.94	-0.52	1.2E-02
R05D11.5	0.94	-0.52	1.2E-02
F26F4.8	0.94	-0.52	1.2E-02
T13H5.3	0.94	-0.52	3.9E-02
R186.8	0.94	-0.52	1.3E-02
T24D3.2	0.94	-0.52	1.6E-02
F18H3.1	0.94	-0.52	1.6E-02
F45H10.2.1	0.94	-0.52	1.7E-02
C41G7.1a	0.94	-0.52	1.0E-02
Y67D8C.6	0.94	-0.52	1.0E-02
K07A1.13	0.94	-0.52	1.1E-02
C56C10.9	0.94	-0.52	1.1E-02
T08A9.5	0.94	-0.52	1.3E-02
Y105C5B.5	0.94	-0.52	1.4E-02
T25B9.8	0.94	-0.52	1.5E-02
Y38F2AL.5	0.94	-0.52	1.2E-02
F35C12.3b.2	0.94	-0.52	1.6E-02
F29B9.10	0.94	-0.52	1.2E-02
Y17G7B.21	0.94	-0.52	1.3E-02

Supporting Table 2: Significant changes after 48h in H_2S			
gene	logFC (48hr)	fold-change	adj.P.Val (48hr)
Y71H2B.6	0.94	-0.52	1.4E-02
Y48C3A.9	0.94	-0.52	1.6E-02
W07E11.4	0.94	-0.52	1.6E-02
Y87G2A.14	0.94	-0.52	1.1E-02
C08F8.7	0.94	-0.52	1.5E-02
W02C12.3h.6	0.94	-0.52	2.9E-02
T26E3.5	0.94	-0.52	1.8E-02
D1086.5	0.94	-0.52	1.3E-02
VH15N14R.1	0.94	-0.52	1.7E-02
Y106G6D.8.2	0.94	-0.52	1.2E-02
Y57A10B.6	0.94	-0.52	1.1E-02
Y80D3A.9	0.93	-0.52	1.4E-02
C17H1.14	0.93	-0.52	1.1E-02
E_BE45912.2	0.93	-0.52	1.2E-02
Y71H2B.3	0.93	-0.52	1.5E-02
Y54G2A.5a.1	0.93	-0.52	1.2E-02
F56B3.9	0.93	-0.52	1.4E-02
C35D10.13	0.93	-0.52	1.2E-02
Y105C5B.8	0.93	-0.52	1.3E-02
F08F8.7.1	0.93	-0.52	1.8E-02
W02H5.3	0.93	-0.52	1.2E-02
W04B5.1	0.93	-0.52	1.5E-02
Y65A5A.1	0.93	-0.52	1.4E-02
C29F9.1	0.93	-0.52	1.4E-02
M01E11.1	0.93	-0.52	2.7E-02
R144.10	0.93	-0.52	1.2E-02
C17E4.2	0.93	-0.52	2.0E-02
ZK1098.5	0.93	-0.52	1.3E-02
ZC262.2b	0.93	-0.52	1.3E-02
C30G12.2	0.93	-0.52	2.8E-02
R10E4.2a.2	0.93	-0.52	2.0E-02
Y38A10A.7	0.93	-0.52	1.6E-02
Y39A3CL.6	0.93	-0.52	1.8E-02
F49F1.5	0.93	-0.53	1.5E-02
F40G12.7	0.93	-0.53	1.1E-02
Y46G5A.34	0.93	-0.53	1.8E-02
C01G10.11b.2	0.93	-0.53	1.1E-02
B0035.2	0.93	-0.53	2.1E-02
C15C7.1.1	0.93	-0.53	1.2E-02
Y113G7B.7	0.93	-0.53	2.0E-02
F56D12.5a.1	0.93	-0.53	1.1E-02
C09H10.2.1	0.93	-0.53	1.3E-02
Y54E10A.7	0.93	-0.53	1.1E-02
R10D12.13a	0.93	-0.53	1.5E-02
W04C9.3	0.92	-0.53	1.2E-02
W05B5.1	0.92	-0.53	1.3E-02
C27F2.9	0.92	-0.53	1.7E-02

Supporting Table 2: Significant changes after 48h in H_2S			
gene	logFC (48hr)	fold-change	adj.P.Val (48hr)
T07F10.5	0.92	-0.53	1.1E-02
Y54G11A.2	0.92	-0.53	1.6E-02
C39B10.3	0.92	-0.53	1.5E-02
C08E3.1	0.92	-0.53	1.4E-02
Y69A2AR.28	0.92	-0.53	1.4E-02
Y14H12B.1a	0.92	-0.53	1.8E-02
B0495.9	0.92	-0.53	1.4E-02
C50F2.7	0.92	-0.53	1.3E-02
F08G12.11	0.92	-0.53	1.2E-02
T20D3.6.1	0.92	-0.53	1.2E-02
AH9.2	0.92	-0.53	1.3E-02
Y43B11AL.1	0.92	-0.53	1.9E-02
B0546.4a	0.92	-0.53	1.4E-02
R10E12.2	0.92	-0.53	1.1E-02
Y66D12A.10	0.92	-0.53	1.9E-02
Y87G2A.10.1	0.92	-0.53	1.4E-02
T01B7.4	0.92	-0.53	1.5E-02
R08E5.3.1	0.92	-0.53	2.7E-02
C09B7.1b	0.92	-0.53	1.2E-02
Y27F2A.6	0.92	-0.53	1.2E-02
D1053.4	0.92	-0.53	1.4E-02
F02H6.3b	0.92	-0.53	1.5E-02
Y55D5A.2	0.92	-0.53	2.0E-02
Y43E12A.3	0.92	-0.53	1.2E-02
Y16E11A.1	0.92	-0.53	1.5E-02
Y51H7C.15	0.92	-0.53	1.3E-02
Y44A6C.1	0.92	-0.53	1.2E-02
C33G8.5	0.92	-0.53	1.5E-02
F25H5.6.1	0.92	-0.53	1.4E-02
H39E23.1a	0.92	-0.53	1.4E-02
Y54E10A.5.2	0.92	-0.53	1.4E-02
K09E9.3	0.92	-0.53	1.3E-02
Y44E3A.3	0.92	-0.53	1.2E-02
H11L12.1	0.92	-0.53	1.4E-02
Y34B4A.3	0.92	-0.53	1.5E-02
Y77E11A.6	0.92	-0.53	1.4E-02
W06A7.3a	0.92	-0.53	1.2E-02
T07G12.12	0.92	-0.53	2.4E-02
F42A6.6	0.92	-0.53	1.4E-02
T08G5.7	0.92	-0.53	1.3E-02
F28F9.4	0.92	-0.53	1.2E-02
Y40C7B.4	0.92	-0.53	3.4E-02
C03H5.7	0.91	-0.53	2.0E-02
C25D7.15	0.91	-0.53	1.2E-02
ZK563.5	0.91	-0.53	1.4E-02
Y59A8B.11	0.91	-0.53	1.2E-02
C50F4.7	0.91	-0.53	1.4E-02

Supporting Table 2: Significant changes after 48h in H_2S			
gene	logFC (48hr)	fold-change	adj.P.Val (48hr)
T05G11.1	0.91	-0.53	1.8E-02
F33G12.2	0.91	-0.53	1.6E-02
M05D6.5.2	0.91	-0.53	1.8E-02
F53E10.2b	0.91	-0.53	1.6E-02
W04C9.2	0.91	-0.53	1.2E-02
Y110A7A.13	0.91	-0.53	3.1E-02
Y55F3AM.3a	0.91	-0.53	1.2E-02
Y116A8C.29	0.91	-0.53	1.6E-02
K04C2.3b	0.91	-0.53	1.6E-02
T11B7.1	0.91	-0.53	1.5E-02
Y106G6H.4	0.91	-0.53	1.2E-02
R10E4.6	0.91	-0.53	1.6E-02
R06C1.3	0.91	-0.53	2.0E-02
C46G7.5	0.91	-0.53	1.2E-02
C08F8.1	0.91	-0.53	1.4E-02
R151.10.2	0.91	-0.53	1.4E-02
F36G3.3	0.91	-0.53	1.4E-02
M142.8	0.91	-0.53	2.2E-02
Y59A8B.20	0.91	-0.53	1.3E-02
C32B5.1b	0.91	-0.53	1.2E-02
T05H10.3	0.91	-0.53	2.0E-02
Y54G2A.50	0.91	-0.53	1.9E-02
F48D6.1.1	0.91	-0.53	1.9E-02
T10H9.8	0.91	-0.53	1.4E-02
D2024.5a.2	0.91	-0.53	2.0E-02
Y9C12A.1	0.91	-0.53	1.4E-02
K08B4.6	0.90	-0.53	1.9E-02
2L52.1	0.90	-0.53	1.7E-02
F52D2.5	0.90	-0.53	1.6E-02
Y6B3B.5b	0.90	-0.53	1.7E-02
W03C9.7.1	0.90	-0.53	2.7E-02
C26E6.12	0.90	-0.53	1.5E-02
F53G12.4	0.90	-0.53	1.4E-02
F49A5.5b	0.90	-0.53	2.1E-02
T23G11.2.1	0.90	-0.54	1.4E-02
T19C4.7	0.90	-0.54	1.4E-02
F08B6.2.1	0.90	-0.54	2.9E-02
H12D21.1	0.90	-0.54	1.5E-02
Y51F10.5	0.90	-0.54	1.7E-02
F53F1.4	0.90	-0.54	2.0E-02
C44B11.4	0.90	-0.54	1.9E-02
K08H2.3	0.90	-0.54	1.4E-02
Y59E9AR.4	0.90	-0.54	1.5E-02
F08B4.7.2	0.90	-0.54	2.0E-02
F37B1.1	0.90	-0.54	2.5E-02
DY3.1	0.90	-0.54	2.1E-02
C04F12.9	0.90	-0.54	1.7E-02

Supporting Table 2: Significant changes after 48h in H_2S			
gene	logFC (48hr)	fold-change	adj.P.Val (48hr)
T13F2.2.1	0.90	-0.54	1.4E-02
F33D4.8	0.90	-0.54	1.6E-02
C15C8.7	0.90	-0.54	2.4E-02
Y66C5A.1	0.90	-0.54	1.4E-02
F56A12.1	0.89	-0.54	2.8E-02
Y59A8B.23	0.89	-0.54	2.0E-02
F52A8.6b	0.89	-0.54	2.0E-02
Y111B2A.11	0.89	-0.54	1.5E-02
C30C11.1	0.89	-0.54	1.6E-02
C09H10.2.2	0.89	-0.54	2.6E-02
H38K22.3	0.89	-0.54	1.7E-02
Y71H2B.11	0.89	-0.54	1.4E-02
R10E4.2a.1	0.89	-0.54	3.3E-02
Y23H5A.8	0.89	-0.54	1.6E-02
W03C9.7.2	0.89	-0.54	2.7E-02
C33C12.10	0.89	-0.54	1.8E-02
Y74C9A.3.1	0.89	-0.54	1.6E-02
Y17G7B.1	0.89	-0.54	2.0E-02
B0228.6	0.89	-0.54	1.7E-02
R04D3.2	0.89	-0.54	1.9E-02
Y37H9A.6.1	0.89	-0.54	1.7E-02
F42A6.1	0.89	-0.54	1.4E-02
C17B7.11	0.89	-0.54	1.7E-02
ZC455.3	0.89	-0.54	1.5E-02
C04F5.7	0.89	-0.54	2.1E-02
Y39B6A.12a	0.89	-0.54	2.1E-02
F37B1.4	0.89	-0.54	1.7E-02
ZK686.1	0.89	-0.54	2.6E-02
C35D10.5	0.89	-0.54	2.2E-02
ZK1073.1.1	0.89	-0.54	2.0E-02
Y37A1B.5	0.89	-0.54	1.6E-02
Y54G11A.13	0.89	-0.54	2.8E-02
R02C2.5	0.89	-0.54	1.4E-02
Y53G8AR.1	0.88	-0.54	1.5E-02
Y105E8B.6	0.88	-0.54	2.6E-02
T16G12.9	0.88	-0.54	1.6E-02
R151.9	0.88	-0.54	1.6E-02
F49F1.6	0.88	-0.54	2.2E-02
C51E3.1	0.88	-0.54	1.7E-02
C14C10.2a	0.88	-0.54	3.4E-02
C06G3.2	0.88	-0.54	1.5E-02
T26C11.5	0.88	-0.54	1.7E-02
Y57G11C.34	0.88	-0.54	1.6E-02
F34D6.1	0.88	-0.54	4.4E-02
C32E8.1	0.88	-0.54	1.6E-02
Y50D7A.11	0.88	-0.54	1.5E-02
ZK1073.1.2	0.88	-0.54	1.6E-02

Supporting Table 2: Significant changes after 48h in H_2S			
gene	logFC (48hr)	fold-change	adj.P.Val (48hr)
ZK632.7.2	0.88	-0.54	1.6E-02
C33A12.1	0.88	-0.54	1.9E-02
R05D11.7	0.88	-0.54	2.5E-02
T01G1.4	0.88	-0.54	1.7E-02
F44G4.6	0.88	-0.54	2.6E-02
M151.7	0.88	-0.54	2.0E-02
F41C6.4	0.88	-0.54	2.1E-02
T28H10.1	0.88	-0.54	2.2E-02
Y57A10A.31	0.88	-0.54	2.1E-02
B0285.3	0.88	-0.54	2.4E-02
C14B1.10.2	0.88	-0.54	2.7E-02
C24H11.5	0.88	-0.55	2.2E-02
K10B2.4	0.88	-0.55	2.5E-02
K11H3.4	0.88	-0.55	1.7E-02
Y54E10A.5.1	0.88	-0.55	2.4E-02
R06F6.4	0.88	-0.55	1.9E-02
Y37H2A.7	0.88	-0.55	1.6E-02
F15E6.1	0.87	-0.55	2.4E-02
Y62H9A.4	0.87	-0.55	1.9E-02
ZK1151.1b	0.87	-0.55	2.6E-02
W07A12.8	0.87	-0.55	2.2E-02
F49D11.3b	0.87	-0.55	1.5E-02
ZK39.6	0.87	-0.55	1.6E-02
F52C9.1a	0.87	-0.55	1.6E-02
F15H10.3	0.87	-0.55	1.6E-02
Y106G6D.8.1	0.87	-0.55	2.0E-02
K08H2.2	0.87	-0.55	4.1E-02
Y34B4A.2	0.87	-0.55	1.8E-02
Y73F8A.31	0.87	-0.55	2.5E-02
ZK1236.9	0.87	-0.55	1.6E-02
B0213.12	0.87	-0.55	1.7E-02
C28D4.3.2	0.87	-0.55	1.5E-02
T04A6.1a	0.87	-0.55	2.0E-02
F57B10.5	0.87	-0.55	2.6E-02
T07D10.6.1	0.87	-0.55	3.7E-02
R148.2	0.87	-0.55	2.2E-02
Y73B6BL.27	0.87	-0.55	1.6E-02
F08B4.3	0.87	-0.55	1.6E-02
Y38E10A.10	0.87	-0.55	1.8E-02
F32D8.9	0.87	-0.55	1.8E-02
R02D3.5.1	0.87	-0.55	1.6E-02
Y51A2D.10	0.87	-0.55	1.6E-02
B0238.12	0.87	-0.55	3.6E-02
F20D6.2	0.87	-0.55	2.1E-02
ZK673.3	0.87	-0.55	2.0E-02
K01D12.14	0.87	-0.55	2.1E-02
F27B3.7	0.87	-0.55	3.2E-02

Supporting Table 2: Significant changes after 48h in H_2S			
gene	logFC (48hr)	fold-change	adj.P.Val (48hr)
C06B3.4	0.87	-0.55	2.0E-02
Y49E10.2	0.87	-0.55	2.9E-02
R08D7.5	0.87	-0.55	1.9E-02
C18D11.3	0.87	-0.55	2.4E-02
F18E2.4	0.87	-0.55	2.2E-02
Y44A6B.4	0.87	-0.55	1.9E-02
F59A3.4	0.87	-0.55	1.8E-02
Y39C12A.8	0.86	-0.55	2.9E-02
F56A8.3b.1	0.86	-0.55	1.9E-02
F46G10.3	0.86	-0.55	2.4E-02
R06F6.7	0.86	-0.55	1.7E-02
T23D8.6	0.86	-0.55	2.3E-02
W02C12.3a.1	0.86	-0.55	2.2E-02
F35F10.1	0.86	-0.55	1.6E-02
W09G3.1a	0.86	-0.55	1.9E-02
F14H12.7	0.86	-0.55	1.6E-02
K11D12.12	0.86	-0.55	1.7E-02
C01G10.11a.2	0.86	-0.55	1.9E-02
ZK353.2	0.86	-0.55	2.1E-02
F56A6.4	0.86	-0.55	1.7E-02
F30A10.11	0.86	-0.55	2.1E-02
F55G11.4	0.86	-0.55	2.5E-02
H12I13.6	0.86	-0.55	1.9E-02
F45F2.9.2	0.86	-0.55	1.7E-02
K12G11.3.1	0.86	-0.55	2.4E-02
R04E5.9	0.86	-0.55	2.4E-02
Y48E1B.12	0.86	-0.55	3.1E-02
C02A12.1	0.86	-0.55	2.8E-02
F57C7.3a	0.86	-0.55	1.8E-02
K07C11.3	0.86	-0.55	2.4E-02
Y18D10A.25	0.86	-0.55	1.8E-02
T23E1.2	0.86	-0.55	1.7E-02
CD4.3.2	0.86	-0.55	2.8E-02
T05G5.7	0.86	-0.55	2.8E-02
F53B1.6	0.86	-0.55	2.0E-02
F57B9.4c	0.86	-0.55	1.9E-02
F01F1.15.1	0.86	-0.55	1.7E-02
F12A10.8	0.86	-0.55	1.7E-02
Y17G7B.4	0.86	-0.55	2.1E-02
F35D11.4	0.85	-0.55	1.7E-02
Y39E4B.7	0.85	-0.55	2.1E-02
T23B5.3c.1	0.85	-0.55	2.0E-02
R10D12.12	0.85	-0.55	2.1E-02
C03D6.6	0.85	-0.55	2.7E-02
F49E8.2	0.85	-0.55	1.8E-02
T07C12.14	0.85	-0.55	2.7E-02
F58E10.7	0.85	-0.55	2.5E-02

Supporting Table 2: Significant changes after 48h in H_2S			
gene	logFC (48hr)	fold-change	adj.P.Val (48hr)
B0511.14b	0.85	-0.55	1.7E-02
T05H10.4	0.85	-0.55	1.8E-02
T24A6.19	0.85	-0.55	2.0E-02
Y45F10D.10	0.85	-0.55	1.9E-02
C14F5.1b	0.85	-0.55	1.7E-02
R10D12.8	0.85	-0.55	2.8E-02
F42A9.6	0.85	-0.55	1.8E-02
F42G8.6	0.85	-0.55	2.9E-02
R05D3.8	0.85	-0.55	1.8E-02
Y56A3A.11	0.85	-0.55	2.0E-02
B0416.7b	0.85	-0.55	1.9E-02
F54F7.3	0.85	-0.55	1.8E-02
W02D7.9	0.85	-0.55	2.1E-02
T12B5.2	0.85	-0.55	4.0E-02
C06G4.5	0.85	-0.55	2.2E-02
F40F4.2	0.85	-0.55	3.1E-02
R151.10.1	0.85	-0.55	1.7E-02
K06C4.9	0.85	-0.56	2.1E-02
Y57E12AR.1	0.85	-0.56	2.6E-02
Y104H12D.1	0.85	-0.56	3.2E-02
C24A1.2b	0.85	-0.56	2.3E-02
Y111B2A.13	0.85	-0.56	2.6E-02
K08H2.7	0.85	-0.56	2.4E-02
ZK75.2	0.85	-0.56	2.0E-02
R01H10.5	0.85	-0.56	1.9E-02
Y49E10.6.1	0.85	-0.56	2.4E-02
K12H4.2	0.85	-0.56	1.9E-02
C26D10.3	0.84	-0.56	2.0E-02
F20D1.10.1	0.84	-0.56	1.9E-02
C16C8.14	0.84	-0.56	2.0E-02
C48B6.2.1	0.84	-0.56	1.9E-02
K10G9.3	0.84	-0.56	1.9E-02
ZK856.14	0.84	-0.56	1.9E-02
T04B2.6	0.84	-0.56	2.0E-02
F45C12.15	0.84	-0.56	2.8E-02
K05B2.2a	0.84	-0.56	1.9E-02
Y39A1A.11	0.84	-0.56	2.4E-02
T01C3.3	0.84	-0.56	2.1E-02
T25C8.1	0.84	-0.56	2.0E-02
C01F6.5	0.84	-0.56	2.2E-02
F26D11.6	0.84	-0.56	3.0E-02
C01F1.2	0.84	-0.56	2.1E-02
CD4.3.1	0.84	-0.56	2.0E-02
F36D3.1.1	0.84	-0.56	2.1E-02
F59A3.9	0.84	-0.56	2.3E-02
F56A8.3a.1	0.84	-0.56	2.0E-02
Y106G6D.1	0.84	-0.56	2.3E-02

Supporting Table 2: Significant changes after 48h in H_2S			
gene	logFC (48hr)	fold-change	adj.P.Val (48hr)
F40D4.13	0.84	-0.56	3.2E-02
C33H5.14.1	0.84	-0.56	4.0E-02
F39E9.11	0.84	-0.56	3.2E-02
F47E1.4.1	0.84	-0.56	2.1E-02
Y50D7A.10.1	0.84	-0.56	2.5E-02
H37A05.4	0.84	-0.56	2.5E-02
F23G4.1	0.84	-0.56	2.2E-02
M05B5.4	0.84	-0.56	2.6E-02
ZC196.5	0.84	-0.56	3.5E-02
Y61A9LA.9	0.84	-0.56	3.0E-02
F07B7.11	0.84	-0.56	3.8E-02
R10H10.1.2	0.83	-0.56	3.9E-02
ZK1320.11	0.83	-0.56	2.9E-02
Y48G1BM.1	0.83	-0.56	2.6E-02
Y43C5A.5	0.83	-0.56	3.1E-02
C01G8.4	0.83	-0.56	2.2E-02
C05C8.9b	0.83	-0.56	2.1E-02
D2007.1	0.83	-0.56	3.3E-02
Y73B6BL.26	0.83	-0.56	2.3E-02
Y65B4BL.4	0.83	-0.56	2.2E-02
Y59E9AL.7	0.83	-0.56	3.1E-02
C48B6.10	0.83	-0.56	3.3E-02
C53D6.3	0.83	-0.56	2.0E-02
Y92H12BL.5	0.83	-0.56	2.2E-02
K11H12.1	0.83	-0.56	2.6E-02
F36A4.14	0.83	-0.56	2.5E-02
F45F2.13	0.83	-0.56	3.0E-02
B0303.15	0.83	-0.56	4.1E-02
C27A12.10	0.83	-0.56	2.6E-02
C08F8.8	0.83	-0.56	4.7E-02
W09H1.3	0.83	-0.56	3.4E-02
T14F9.5	0.83	-0.56	3.2E-02
H12D21.3	0.83	-0.56	3.1E-02
F01F1.10a	0.83	-0.56	3.2E-02
C41G11.1b	0.83	-0.56	2.5E-02
K06A9.3	0.83	-0.56	2.4E-02
C01F6.9	0.83	-0.56	2.8E-02
Y92H12BL.1	0.83	-0.56	2.1E-02
B0035.8	0.83	-0.56	3.9E-02
C06H2.6.1	0.83	-0.56	2.5E-02
C30H6.8	0.82	-0.56	2.4E-02
C40H5.1	0.82	-0.56	2.1E-02
T19D2.3	0.82	-0.56	2.3E-02
Y71H2AM.6	0.82	-0.56	2.9E-02
F48G7.4	0.82	-0.57	2.4E-02
C49C3.15	0.82	-0.57	2.9E-02
ZK121.1a	0.82	-0.57	2.3E-02

Supporting Table 2: Significant changes after 48h in H_2S			
gene	logFC (48hr)	fold-change	adj.P.Val (48hr)
F14D2.8	0.82	-0.57	3.1E-02
W06D4.5	0.82	-0.57	2.2E-02
Y48E1B.9	0.82	-0.57	2.5E-02
C53D6.11	0.82	-0.57	2.8E-02
F10D7.5e	0.82	-0.57	3.2E-02
T11A5.5	0.82	-0.57	3.5E-02
R06C7.2	0.82	-0.57	3.2E-02
F47G3.3	0.82	-0.57	3.1E-02
K02E11.6	0.82	-0.57	2.6E-02
T23D8.5	0.82	-0.57	3.4E-02
Y49F6A.1	0.82	-0.57	2.5E-02
F43G6.10	0.82	-0.57	2.6E-02
Y71H2AM.4.2	0.82	-0.57	2.4E-02
ZK563.7	0.82	-0.57	2.2E-02
F31F4.4	0.82	-0.57	2.6E-02
Y71H2AM.4.1	0.82	-0.57	3.2E-02
T06D8.7.1	0.82	-0.57	2.5E-02
ZK250.7	0.82	-0.57	2.4E-02
C07D10.1	0.82	-0.57	2.6E-02
F14D2.12	0.82	-0.57	2.5E-02
M01D7.7b	0.81	-0.57	2.7E-02
T08D2.7	0.81	-0.57	2.4E-02
M88.7	0.81	-0.57	2.4E-02
C33F10.7a	0.81	-0.57	3.2E-02
F32E10.9	0.81	-0.57	2.9E-02
C41D11.1	0.81	-0.57	2.5E-02
K07H8.9	0.81	-0.57	3.3E-02
Y71F9AR.2	0.81	-0.57	2.5E-02
R07E5.4	0.81	-0.57	2.7E-02
Y65A5A.3	0.81	-0.57	2.4E-02
T08G5.10	0.81	-0.57	4.2E-02
Y62E10A.5b.1	0.81	-0.57	2.5E-02
F45F2.3	0.81	-0.57	3.2E-02
Y111B2A.24	0.81	-0.57	3.7E-02
ZC13.1a	0.81	-0.57	2.4E-02
F35C12.3a	0.81	-0.57	2.5E-02
Y80D3A.3	0.81	-0.57	3.6E-02
D2096.10	0.81	-0.57	2.3E-02
F49A5.5a	0.81	-0.57	4.5E-02
ZK632.9.1	0.81	-0.57	3.2E-02
ZC262.4	0.81	-0.57	2.6E-02
Y39G10AR.10.1	0.81	-0.57	4.3E-02
F20D12.6a	0.81	-0.57	3.0E-02
ZK643.6	0.81	-0.57	2.7E-02
Y39G10AR.12a	0.81	-0.57	3.2E-02
D2030.12	0.81	-0.57	2.4E-02
M03B6.5	0.81	-0.57	4.0E-02

Supporting Table 2: Significant changes after 48h in H_2S			
gene	logFC (48hr)	fold-change	adj.P.Val (48hr)
T28D6.3	0.81	-0.57	2.8E-02
Y41C4A.4c	0.81	-0.57	2.8E-02
C03G5.2	0.81	-0.57	2.5E-02
F13C5.1	0.81	-0.57	4.4E-02
ZK662.3a	0.81	-0.57	3.0E-02
Y39B6A.13	0.81	-0.57	2.5E-02
EEED8.15	0.81	-0.57	3.2E-02
T22C1.4	0.81	-0.57	2.8E-02
T12C9.1	0.81	-0.57	4.1E-02
H02I12.5	0.81	-0.57	2.5E-02
K10D2.5	0.81	-0.57	2.7E-02
Y43C5A.3	0.80	-0.57	2.3E-02
F56E10.2	0.80	-0.57	3.8E-02
C25G4.2	0.80	-0.57	2.7E-02
C18A3.10	0.80	-0.57	2.8E-02
ZK1127.3	0.80	-0.57	2.5E-02
ZK593.2	0.80	-0.57	2.5E-02
F55G1.6	0.80	-0.57	3.2E-02
F07C3.9	0.80	-0.57	2.4E-02
T13B5.7	0.80	-0.57	2.6E-02
T04A8.10	0.80	-0.57	2.5E-02
R13A1.10	0.80	-0.57	3.1E-02
C44B7.2b	0.80	-0.57	2.5E-02
F55C12.7.1	0.80	-0.57	2.5E-02
Y10G11A.1	0.80	-0.57	2.4E-02
B0273.3	0.80	-0.57	3.4E-02
F26E4.12	0.80	-0.57	3.5E-02
C03G5.12	0.80	-0.57	2.8E-02
K03E6.4	0.80	-0.57	2.4E-02
K09C4.2	0.80	-0.57	2.9E-02
F53H8.4	0.80	-0.57	4.0E-02
B0252.4a	0.80	-0.57	2.6E-02
Y80D3A.7	0.80	-0.57	4.1E-02
C01B4.5	0.80	-0.57	4.5E-02
R09B5.3.2	0.80	-0.57	4.8E-02
F16H11.3	0.80	-0.57	2.8E-02
F22A3.2	0.80	-0.58	4.4E-02
F21C10.10.2	0.80	-0.58	2.5E-02
F20C5.6	0.80	-0.58	3.2E-02
C14F5.1a	0.80	-0.58	2.6E-02
E04D5.5	0.80	-0.58	2.4E-02
C03G5.8	0.80	-0.58	2.8E-02
F41E6.4a	0.80	-0.58	4.3E-02
T07A9.12b	0.80	-0.58	3.2E-02
F20B6.7	0.80	-0.58	3.6E-02
ZK973.9	0.80	-0.58	2.9E-02
Y22D7AL.9	0.80	-0.58	2.6E-02

Supporting Table 2: Significant changes after 48h in H_2S			
gene	logFC (48hr)	fold-change	adj.P.Val (48hr)
ZK858.5	0.79	-0.58	2.6E-02
T24H10.6	0.79	-0.58	3.5E-02
ZK813.3	0.79	-0.58	3.5E-02
C11D2.7.1	0.79	-0.58	3.1E-02
Y47G6A.24	0.79	-0.58	3.1E-02
F52C6.11	0.79	-0.58	2.9E-02
ZK1098.7.1	0.79	-0.58	3.4E-02
C09B7.2	0.79	-0.58	4.0E-02
C53D5.1a	0.79	-0.58	2.8E-02
E01A2.1	0.79	-0.58	3.2E-02
Y75B12B.1	0.79	-0.58	2.8E-02
Y55F3C.10	0.79	-0.58	2.7E-02
F32D1.6	0.79	-0.58	3.1E-02
Y113G7B.5b	0.79	-0.58	4.0E-02
C14A4.7a	0.79	-0.58	3.0E-02
F58B6.1	0.79	-0.58	3.5E-02
B0252.4b	0.79	-0.58	3.0E-02
Y71D11A.2b	0.79	-0.58	3.3E-02
Y39A1A.14	0.79	-0.58	3.2E-02
W05B10.2	0.79	-0.58	3.0E-02
F49E12.10	0.79	-0.58	3.5E-02
C02F5.4	0.79	-0.58	2.8E-02
Y105E8A.11	0.79	-0.58	2.6E-02
Y56A3A.33	0.79	-0.58	4.7E-02
F54H12.2	0.79	-0.58	3.1E-02
Y39B6A.3	0.79	-0.58	3.7E-02
F58E10.2	0.79	-0.58	3.6E-02
F35H10.3	0.79	-0.58	2.8E-02
F18E9.2	0.79	-0.58	3.3E-02
F10E7.2	0.79	-0.58	2.7E-02
F56A8.8	0.79	-0.58	2.8E-02
H12D21.12	0.79	-0.58	2.9E-02
C35A5.6b	0.79	-0.58	3.7E-02
F57B1.5	0.79	-0.58	2.8E-02
T01A4.2	0.79	-0.58	2.8E-02
T08D2.4	0.79	-0.58	2.8E-02
F08B6.3	0.79	-0.58	4.0E-02
Y49E10.14	0.78	-0.58	2.7E-02
C01G10.11a.1	0.78	-0.58	2.7E-02
Y54E10BR.3	0.78	-0.58	3.1E-02
Y26D4A.10	0.78	-0.58	3.9E-02
T23F2.3	0.78	-0.58	3.2E-02
Y62E10A.5a.1	0.78	-0.58	2.8E-02
F28B4.4	0.78	-0.58	3.6E-02
Y49E10.3b	0.78	-0.58	2.9E-02
F57C9.6	0.78	-0.58	3.0E-02
M01F1.8a	0.78	-0.58	3.5E-02

Supporting Table 2: Significant changes after 48h in H_2S			
gene	logFC (48hr)	fold-change	adj.P.Val (48hr)
F54F11.1	0.78	-0.58	2.9E-02
D1054.2	0.78	-0.58	3.3E-02
F56B6.5a	0.78	-0.58	3.3E-02
C03G6.17	0.78	-0.58	4.9E-02
Y73F4A.3	0.78	-0.58	3.4E-02
F08G12.4	0.78	-0.58	3.6E-02
W04A8.4	0.78	-0.58	3.1E-02
R08C7.3.3	0.78	-0.58	4.1E-02
K09F6.11	0.78	-0.58	3.0E-02
C34D4.14	0.78	-0.58	4.9E-02
ZK938.7	0.78	-0.58	2.8E-02
C24B5.4	0.78	-0.58	2.9E-02
ZK185.2	0.78	-0.58	3.0E-02
F32E10.8	0.78	-0.58	3.2E-02
W06A7.5	0.78	-0.58	3.1E-02
R09B3.4	0.78	-0.58	4.6E-02
F02E9.5	0.78	-0.58	3.2E-02
B0280.10	0.78	-0.58	3.0E-02
W08E12.1	0.78	-0.58	3.1E-02
Y18D10A.10	0.78	-0.58	3.8E-02
T24A6.1	0.78	-0.58	3.0E-02
K07A1.3	0.78	-0.58	3.7E-02
M163.3	0.78	-0.58	2.9E-02
F09F7.7a	0.78	-0.58	4.3E-02
ZK512.1	0.78	-0.58	3.2E-02
F35C5.2	0.77	-0.58	3.0E-02
C08F11.3	0.77	-0.58	4.5E-02
Y113G7B.5a	0.77	-0.58	4.0E-02
R53.5	0.77	-0.59	3.5E-02
Y71F9B.3.1	0.77	-0.59	3.6E-02
F53C11.8.1	0.77	-0.59	3.2E-02
E02H1.1	0.77	-0.59	3.0E-02
Y43F4B.4	0.77	-0.59	3.2E-02
R05H5.5	0.77	-0.59	3.2E-02
Y57G11C.33	0.77	-0.59	4.6E-02
Y38H6C.7	0.77	-0.59	3.9E-02
Y50D4A.2.1	0.77	-0.59	3.5E-02
Y45G5AM.9c	0.77	-0.59	3.1E-02
F40H6.6	0.77	-0.59	3.0E-02
D2013.10	0.77	-0.59	3.2E-02
R02D3.5.2	0.77	-0.59	2.9E-02
C18E9.4	0.77	-0.59	3.1E-02
F13G3.11.1	0.77	-0.59	3.1E-02
K08D10.4	0.77	-0.59	4.1E-02
Y54E10BL.4	0.77	-0.59	3.8E-02
Y71F9B.9	0.77	-0.59	4.4E-02
Y67D2.4	0.77	-0.59	3.9E-02

Supporting Table 2: Significant changes after 48h in H_2S			
gene	logFC (48hr)	fold-change	adj.P.Val (48hr)
C35E7.8	0.77	-0.59	3.2E-02
ZK1248.11.2	0.77	-0.59	3.9E-02
B0035.16	0.77	-0.59	3.5E-02
F07B7.9	0.77	-0.59	4.2E-02
W09G3.3	0.77	-0.59	3.8E-02
Y57A10A.14	0.76	-0.59	3.6E-02
T04F3.4	0.76	-0.59	4.2E-02
T01C3.7.3	0.76	-0.59	4.4E-02
W02A11.1	0.76	-0.59	3.3E-02
C46E10.9	0.76	-0.59	4.2E-02
F57G4.8	0.76	-0.59	3.1E-02
F25H8.1	0.76	-0.59	3.2E-02
W07A12.7	0.76	-0.59	3.6E-02
W02C12.3g.2	0.76	-0.59	3.6E-02
F54E7.1a	0.76	-0.59	4.7E-02
F13B9.8b	0.76	-0.59	4.5E-02
C28G1.2	0.76	-0.59	3.7E-02
C08H9.11	0.76	-0.59	3.8E-02
R08E5.3.2	0.76	-0.59	4.1E-02
F25B4.8b	0.76	-0.59	4.4E-02
F42G9.7	0.76	-0.59	3.1E-02
Y95D11A.1.1	0.76	-0.59	4.5E-02
T22F3.12	0.76	-0.59	3.2E-02
F25G6.8.1	0.76	-0.59	3.7E-02
K08E4.5	0.76	-0.59	3.3E-02
C15C6.1	0.76	-0.59	3.2E-02
F41G4.8	0.76	-0.59	3.4E-02
Y59A8B.25	0.76	-0.59	4.4E-02
K02E7.5	0.76	-0.59	3.2E-02
F13H6.5	0.76	-0.59	3.2E-02
Y57G11C.4	0.76	-0.59	4.0E-02
T05E11.6	0.76	-0.59	3.9E-02
F14B4.1	0.76	-0.59	4.2E-02
H37A05.2	0.76	-0.59	3.2E-02
Y38F2AR.2.1	0.76	-0.59	4.0E-02
T27A3.6	0.75	-0.59	4.3E-02
H12D21.14	0.75	-0.59	3.6E-02
T07A9.12a	0.75	-0.59	3.5E-02
F44F1.5	0.75	-0.59	4.5E-02
R05G6.4	0.75	-0.59	3.9E-02
F44F4.1	0.75	-0.59	4.2E-02
D2007.5.1	0.75	-0.59	3.5E-02
Y55F3AM.3c.2	0.75	-0.59	3.6E-02
M18.6	0.75	-0.59	3.5E-02
ZK652.11.1	0.75	-0.59	4.5E-02
Y39E4B.8	0.75	-0.59	3.6E-02
F42G2.6	0.75	-0.59	4.1E-02

Supporting Table 2: Significant changes after 48h in H_2S			
gene	logFC (48hr)	fold-change	adj.P.Val (48hr)
T26C5.4	0.75	-0.59	4.7E-02
T05C12.5	0.75	-0.59	4.1E-02
Y116A8C.3	0.75	-0.60	4.1E-02
C34E10.11	0.75	-0.60	3.6E-02
Y73B6BL.9b	0.75	-0.60	4.9E-02
Y66H1A.3.1	0.75	-0.60	3.8E-02
W02C12.3g.1	0.75	-0.60	4.5E-02
H01G02.1	0.75	-0.60	3.5E-02
F14H3.3	0.75	-0.60	4.5E-02
F37C12.10	0.75	-0.60	3.6E-02
F46H6.1.1	0.75	-0.60	3.8E-02
C27F2.7	0.75	-0.60	3.6E-02
F43G6.8	0.75	-0.60	3.9E-02
Y65A5A.2	0.74	-0.60	4.8E-02
Y48C3A.11	0.74	-0.60	3.5E-02
T20D4.19	0.74	-0.60	4.0E-02
F09E8.1	0.74	-0.60	3.6E-02
R11D1.9	0.74	-0.60	3.8E-02
Y38C1AA.13	0.74	-0.60	4.6E-02
Y17G7B.12	0.74	-0.60	3.9E-02
F32H5.6b	0.74	-0.60	4.9E-02
F41G3.6	0.74	-0.60	4.0E-02
Y41C4A.8	0.74	-0.60	3.6E-02
K09H11.11	0.74	-0.60	4.4E-02
W03A5.3	0.74	-0.60	3.7E-02
T01C1.4	0.74	-0.60	3.5E-02
C17H1.9	0.74	-0.60	3.7E-02
Y38C1AA.4	0.74	-0.60	3.7E-02
K03H1.3	0.74	-0.60	3.6E-02
Y48G9A.11	0.74	-0.60	3.5E-02
Y37H2A.9	0.74	-0.60	3.6E-02
C03G5.11	0.74	-0.60	4.5E-02
F58B3.2	0.74	-0.60	3.6E-02
F45F2.6	0.74	-0.60	3.6E-02
Y39G10AR.3	0.74	-0.60	3.6E-02
W05F2.3	0.74	-0.60	3.6E-02
T26A5.7a	0.74	-0.60	4.5E-02
C08B11.6.2	0.74	-0.60	3.7E-02
C25E10.7	0.74	-0.60	3.6E-02
T04D3.1	0.74	-0.60	4.1E-02
Y59C2A.2	0.74	-0.60	4.7E-02
T26C12.2	0.74	-0.60	4.6E-02
Y53F4B.14	0.74	-0.60	4.0E-02
Y57A10A.18	0.74	-0.60	4.6E-02
B0410.2a	0.73	-0.60	4.2E-02
F25E2.3	0.73	-0.60	4.7E-02
Y51B9A.9	0.73	-0.60	4.9E-02

Supporting Table 2: Significant changes after 48h in H_2S			
gene	logFC (48hr)	fold-change	adj.P.Val (48hr)
C06E7.1c	0.73	-0.60	4.8E-02
C55B6.1a	0.73	-0.60	3.7E-02
K11D2.4a	0.73	-0.60	4.5E-02
D1007.8	0.73	-0.60	4.1E-02
ZC262.8.1	0.73	-0.60	4.9E-02
C50C3.7	0.73	-0.60	3.7E-02
C12C8.3a	0.73	-0.60	4.0E-02
T03F1.10	0.73	-0.60	4.4E-02
ZK507.4	0.73	-0.60	4.3E-02
Y79H2A.6	0.73	-0.60	3.7E-02
K04F10.2	0.73	-0.60	4.3E-02
Y119D3B.7	0.73	-0.60	4.5E-02
F56A8.4	0.73	-0.60	4.3E-02
B0272.2	0.73	-0.60	4.3E-02
ZK1098.4	0.73	-0.60	4.5E-02
Y92H12A.4	0.73	-0.60	5.0E-02
T13C5.6	0.73	-0.60	3.8E-02
Y55F3AM.3c.1	0.73	-0.60	3.9E-02
T09A12.2a	0.73	-0.60	4.6E-02
ZK177.1	0.73	-0.60	4.0E-02
ZK1248.11.1	0.73	-0.60	3.9E-02
R119.1	0.73	-0.60	4.5E-02
C17H12.11	0.73	-0.60	4.4E-02
Y80D3A.4	0.73	-0.60	4.9E-02
T22A3.2	0.73	-0.60	4.3E-02
C34D4.12	0.73	-0.60	4.7E-02
F10G7.2.1	0.73	-0.60	4.2E-02
W08E12.8	0.73	-0.60	4.0E-02
M151.4	0.73	-0.60	4.3E-02
K11D2.2	0.72	-0.61	5.0E-02
T19H12.2.2	0.72	-0.61	4.5E-02
C05C12.6	0.72	-0.61	4.3E-02
Y59A8B.13	0.72	-0.61	3.9E-02
R53.2	0.72	-0.61	4.2E-02
F15D4.6	0.72	-0.61	4.0E-02
T24B8.2	0.72	-0.61	4.4E-02
K12B6.5	0.72	-0.61	4.2E-02
Y50D4C.3	0.72	-0.61	4.5E-02
C04E6.7	0.72	-0.61	4.6E-02
F38E11.9	0.72	-0.61	4.5E-02
C17F4.5	0.72	-0.61	3.9E-02
T21C9.5.1	0.72	-0.61	4.5E-02
C17H11.5	0.72	-0.61	4.0E-02
T10C6.14	0.72	-0.61	4.5E-02
Y105C5A.12	0.72	-0.61	5.0E-02
W05E10.3.1	0.72	-0.61	4.3E-02
C05E11.8	0.72	-0.61	4.2E-02

Supporting Table 2: Significant changes after 48h in H_2S			
gene	logFC (48hr)	fold-change	adj.P.Val (48hr)
T08G5.12	0.72	-0.61	4.3E-02
H03E18.2	0.72	-0.61	4.8E-02
Y38F1A.5.1	0.72	-0.61	4.7E-02
Y80D3A.11	0.72	-0.61	4.0E-02
H35N09.2	0.72	-0.61	4.4E-02
B0024.13a	0.72	-0.61	4.5E-02
ZC334.3	0.72	-0.61	4.5E-02
R10E4.2c	0.72	-0.61	4.7E-02
Y69H2.9	0.72	-0.61	4.8E-02
E01G4.5	0.72	-0.61	4.5E-02
F31D4.8	0.72	-0.61	4.5E-02
Y47G6A.2.1	0.72	-0.61	4.2E-02
F42F12.6	0.72	-0.61	4.3E-02
Y57G7A.1	0.72	-0.61	4.7E-02
Y73F8A.26	0.72	-0.61	4.1E-02
F59E11.16	0.72	-0.61	4.5E-02
C35B1.8	0.71	-0.61	4.2E-02
W09G10.3	0.71	-0.61	4.9E-02
F02C12.3	0.71	-0.61	4.9E-02
F27E5.2	0.71	-0.61	4.3E-02
T27D1.3	0.71	-0.61	4.4E-02
F49B2.5	0.71	-0.61	4.4E-02
B0034.5	0.71	-0.61	4.1E-02
F07F6.8	0.71	-0.61	4.7E-02
F21C3.3	0.71	-0.61	4.8E-02
R05D7.5	0.71	-0.61	4.9E-02
F13G3.11.2	0.71	-0.61	4.8E-02
F01F1.15.2	0.71	-0.61	4.4E-02
Y73B6BL.35	0.71	-0.61	4.3E-02
C05C10.1	0.71	-0.61	4.5E-02
Y73F4A.1	0.71	-0.61	4.5E-02
C03G5.10	0.70	-0.61	4.9E-02
K10C2.7	0.70	-0.61	4.8E-02
C36C9.3	0.70	-0.61	4.5E-02
Y49F6B.2	0.70	-0.62	4.7E-02
C32H11.1	0.70	-0.62	4.6E-02
F26D10.10	0.70	-0.62	4.7E-02
B0286.6	0.70	-0.62	4.5E-02
Y113G7B.18	0.70	-0.62	4.7E-02
T12A2.8.1	0.70	-0.62	4.7E-02
T16G1.7	0.70	-0.62	5.0E-02
ZK470.2b.1	0.70	-0.62	4.6E-02
T08A9.4	0.70	-0.62	5.0E-02
F07G6.7	0.69	-0.62	4.8E-02
F35A5.2	0.69	-0.62	4.7E-02
K01C8.7	0.69	-0.62	4.7E-02
T14F9.4a.1	0.69	-0.62	4.8E-02

Supporting Table 2: Significant changes after 48h in H_2S			
gene	logFC (48hr)	fold-change	adj.P.Val (48hr)
F28D1.3	0.69	-0.62	4.7E-02
F08G12.2	0.69	-0.62	4.8E-02
Y73E7A.7	0.69	-0.62	4.9E-02
K07C5.5	0.69	-0.62	4.8E-02
C24B5.2b.2	0.68	-0.62	4.9E-02
T07H6.3a	-0.69	-1.61	5.0E-02
AC7.2a.2	-0.69	-1.61	4.9E-02
K07A3.2b	-0.69	-1.61	4.9E-02
C44C10.9	-0.69	-1.61	4.9E-02
Y48G9A.1	-0.69	-1.61	4.9E-02
F36D3.8	-0.69	-1.61	4.9E-02
F41D9.3b.1	-0.69	-1.61	5.0E-02
T28F12.2c	-0.69	-1.61	5.0E-02
D1022.6	-0.69	-1.61	5.0E-02
C47E12.11	-0.69	-1.62	5.0E-02
ZK813.6	-0.69	-1.62	4.7E-02
K08A2.2	-0.70	-1.62	4.8E-02
ZC328.2	-0.70	-1.62	4.8E-02
C24H12.2	-0.70	-1.62	4.9E-02
T20F5.5	-0.70	-1.62	4.9E-02
T23F4.3	-0.70	-1.62	4.8E-02
K05B2.5c	-0.70	-1.62	4.9E-02
F55G11.2	-0.70	-1.63	4.8E-02
F55A11.11	-0.70	-1.63	4.5E-02
Y18D10A.21	-0.70	-1.63	4.4E-02
B0432.5b	-0.70	-1.63	4.9E-02
C18A3.8	-0.70	-1.63	5.0E-02
R11F4.1.1	-0.70	-1.63	4.6E-02
C09H5.7	-0.70	-1.63	4.6E-02
Y32B12C.1	-0.70	-1.63	4.7E-02
F13C5.6b	-0.70	-1.63	4.5E-02
M176.1	-0.70	-1.63	4.5E-02
M110.4b	-0.71	-1.63	4.7E-02
ZK1248.2	-0.71	-1.63	4.8E-02
F35G8.1	-0.71	-1.63	4.7E-02
C10G11.10	-0.71	-1.63	4.6E-02
F11C1.5b	-0.71	-1.63	4.4E-02
T01G6.7	-0.71	-1.63	4.7E-02
Y71H2B.7.1	-0.71	-1.63	4.8E-02
T04D1.4	-0.71	-1.63	4.8E-02
ZK418.2b	-0.71	-1.63	4.5E-02
F28H6.1a	-0.71	-1.64	4.9E-02
T09A12.1	-0.71	-1.64	4.3E-02
C48D1.3.1	-0.71	-1.64	4.3E-02
C08F11.13.2	-0.71	-1.64	4.9E-02
Y22D7AR.9	-0.71	-1.64	4.5E-02
F09B9.2a	-0.71	-1.64	4.4E-02

Supporting Table 2: Significant changes after 48h in H_2S			
gene	logFC (48hr)	fold-change	adj.P.Val (48hr)
Y54G2A.47	-0.71	-1.64	4.3E-02
W03G11.3	-0.71	-1.64	4.2E-02
Y34B4A.8	-0.71	-1.64	5.0E-02
C30H6.11	-0.71	-1.64	4.6E-02
W07G1.5a	-0.71	-1.64	4.6E-02
H06H21.9	-0.71	-1.64	4.2E-02
ZC204.13	-0.71	-1.64	4.3E-02
F41E7.3	-0.71	-1.64	4.3E-02
C35A11.2	-0.71	-1.64	4.5E-02
Y59E9AR.1	-0.71	-1.64	4.2E-02
T04F8.6	-0.72	-1.64	4.3E-02
ZK1290.7	-0.72	-1.64	4.7E-02
F39G3.2	-0.72	-1.64	4.1E-02
C17B7.5	-0.72	-1.64	4.4E-02
F35B3.5a	-0.72	-1.64	4.2E-02
H03A11.1	-0.72	-1.64	4.5E-02
F32B6.4	-0.72	-1.64	4.5E-02
T01B4.2	-0.72	-1.64	4.1E-02
Y54G11B.1	-0.72	-1.64	4.5E-02
T22D1.12	-0.72	-1.64	4.4E-02
C05D9.9a	-0.72	-1.64	4.8E-02
Y92H12A.2	-0.72	-1.64	4.5E-02
C25E10.5	-0.72	-1.64	4.1E-02
R03E9.4	-0.72	-1.65	4.7E-02
F15D3.4	-0.72	-1.65	4.7E-02
C39D10.11	-0.72	-1.65	4.3E-02
F23H11.8a	-0.72	-1.65	4.9E-02
F46G11.2	-0.72	-1.65	4.3E-02
B0207.11	-0.72	-1.65	4.0E-02
C46F4.2.1	-0.72	-1.65	4.2E-02
C25D7.12	-0.72	-1.65	4.8E-02
W01A11.4	-0.72	-1.65	4.9E-02
C14H10.2	-0.72	-1.65	4.0E-02
R03E9.3b	-0.72	-1.65	4.1E-02
F20A1.6a	-0.72	-1.65	4.9E-02
F22E5.12	-0.72	-1.65	4.3E-02
F27C1.1	-0.72	-1.65	4.5E-02
T05A10.1i	-0.72	-1.65	4.3E-02
F55D10.5	-0.72	-1.65	4.7E-02
T01B11.2b	-0.72	-1.65	4.2E-02
Y38H8A.4	-0.72	-1.65	4.2E-02
B0034.3c.1	-0.72	-1.65	4.7E-02
F55H2.6.2	-0.72	-1.65	4.1E-02
C09B9.2	-0.72	-1.65	4.2E-02
C34D10.2	-0.72	-1.65	4.8E-02
F57H12.7	-0.72	-1.65	4.4E-02
Y54G2A.33	-0.72	-1.65	4.1E-02

Supporting Table 2: Significant changes after 48h in H_2S			
gene	logFC (48hr)	fold-change	adj.P.Val (48hr)
C54A12.4	-0.72	-1.65	4.7E-02
R31.1	-0.72	-1.65	3.9E-02
C49A9.5	-0.73	-1.65	3.9E-02
F49E2.2a.1	-0.73	-1.65	4.1E-02
F46B6.2	-0.73	-1.65	4.1E-02
F45E10.1a	-0.73	-1.65	3.9E-02
C47A10.2	-0.73	-1.66	4.9E-02
ZK930.7	-0.73	-1.66	3.9E-02
F13E6.6	-0.73	-1.66	4.0E-02
R08A2.2	-0.73	-1.66	3.9E-02
F10D11.5	-0.73	-1.66	3.9E-02
C55C3.1	-0.73	-1.66	4.2E-02
ZK669.1a	-0.73	-1.66	3.9E-02
Y45G5AM.1a.2	-0.73	-1.66	3.7E-02
C15C8.6	-0.73	-1.66	4.6E-02
C11G10.2	-0.73	-1.66	4.2E-02
C53D6.10	-0.73	-1.66	3.8E-02
F41G3.5	-0.73	-1.66	3.9E-02
ZK673.6	-0.73	-1.66	4.3E-02
R05H5.2	-0.73	-1.66	4.1E-02
R11G10.1a	-0.73	-1.66	4.0E-02
ZC482.5	-0.73	-1.66	4.9E-02
C34B4.1b	-0.73	-1.66	4.2E-02
C18H9.8	-0.73	-1.66	4.5E-02
F26F12.2	-0.73	-1.66	4.7E-02
C23G10.1a	-0.73	-1.66	3.8E-02
Y71G12B.33	-0.73	-1.66	3.9E-02
ZK856.5	-0.73	-1.66	4.5E-02
AC7.2b.2	-0.73	-1.66	3.8E-02
T01B10.2	-0.73	-1.66	4.1E-02
F47B3.7	-0.74	-1.66	4.1E-02
Y45F10B.10	-0.74	-1.67	4.3E-02
C49C8.5.1	-0.74	-1.67	4.3E-02
W03F11.3	-0.74	-1.67	5.0E-02
Y38F2AL.1	-0.74	-1.67	4.1E-02
Y54G2A.5a.2	-0.74	-1.67	3.7E-02
W02B12.12a	-0.74	-1.67	3.8E-02
C01G12.9	-0.74	-1.67	3.7E-02
C43G2.3	-0.74	-1.67	4.8E-02
H23L24.4	-0.74	-1.67	4.1E-02
C14A6.6	-0.74	-1.67	4.5E-02
T01B10.4b	-0.74	-1.67	4.0E-02
C46E10.1	-0.74	-1.67	3.9E-02
C17F3.3	-0.74	-1.67	4.0E-02
T19B10.5	-0.74	-1.67	4.4E-02
F32D8.8	-0.74	-1.67	4.2E-02
F35F10.13	-0.74	-1.67	4.8E-02

Supporting Table 2: Significant changes after 48h in H_2S			
gene	logFC (48hr)	fold-change	adj.P.Val (48hr)
T06F4.2b	-0.74	-1.67	4.3E-02
C30F12.5	-0.74	-1.67	4.1E-02
T22B7.7	-0.74	-1.67	4.2E-02
W05F2.7	-0.74	-1.67	4.4E-02
K06A9.2	-0.74	-1.67	3.7E-02
F08C6.1b	-0.74	-1.67	4.8E-02
T15B12.2	-0.74	-1.67	3.6E-02
ZK6.6	-0.74	-1.67	4.0E-02
C53C9.3c	-0.74	-1.67	3.6E-02
F18C5.5	-0.74	-1.67	3.5E-02
F14H12.2	-0.74	-1.67	3.6E-02
T06F4.1b	-0.74	-1.67	4.2E-02
Y66H1B.3.1	-0.74	-1.67	4.5E-02
F37C4.6.2	-0.74	-1.67	3.6E-02
Y54F10AM.8.1	-0.74	-1.67	3.9E-02
F57C7.2a	-0.74	-1.67	3.9E-02
B0024.8	-0.74	-1.67	4.0E-02
F57C12.5d.1	-0.74	-1.68	4.0E-02
Y105C5B.18	-0.74	-1.68	3.4E-02
ZK813.5	-0.74	-1.68	3.5E-02
C06H2.5	-0.75	-1.68	3.9E-02
T05A10.1a	-0.75	-1.68	3.5E-02
C15B12.9	-0.75	-1.68	4.0E-02
M02B1.1	-0.75	-1.68	3.6E-02
C35E7.9	-0.75	-1.68	3.9E-02
W02B12.1	-0.75	-1.68	4.0E-02
T28F2.4a	-0.75	-1.68	3.5E-02
F28E10.1a	-0.75	-1.68	3.8E-02
C01G12.2	-0.75	-1.68	3.7E-02
T10B10.8	-0.75	-1.68	4.3E-02
F07C3.4	-0.75	-1.68	4.4E-02
B0336.11b	-0.75	-1.68	3.6E-02
Y73B3A.5	-0.75	-1.68	3.5E-02
F07A11.6d	-0.75	-1.68	4.3E-02
F35C5.5a	-0.75	-1.68	3.4E-02
ZK418.6	-0.75	-1.68	3.5E-02
Y39A3CL.2	-0.75	-1.68	4.4E-02
C17H11.6c.1	-0.75	-1.68	3.7E-02
T27E4.7.1	-0.75	-1.68	3.9E-02
C15C8.1	-0.75	-1.68	3.9E-02
C08H9.13	-0.75	-1.68	4.7E-02
K09E3.4	-0.75	-1.68	4.1E-02
F58A4.7d	-0.75	-1.68	4.8E-02
F09G2.1	-0.75	-1.69	4.9E-02
C15C6.2b	-0.75	-1.69	4.1E-02
T28H11.8.1	-0.75	-1.69	3.7E-02
F22F4.3	-0.75	-1.69	3.6E-02

Supporting Table 2: Significant changes after 48h in H_2S			
gene	logFC (48hr)	fold-change	adj.P.Val (48hr)
Y48G9A.9a	-0.75	-1.69	3.6E-02
T23F6.3	-0.75	-1.69	4.0E-02
Y45G5AM.1b	-0.76	-1.69	4.3E-02
C18C4.2	-0.76	-1.69	4.5E-02
Y59E9AL.6	-0.76	-1.69	4.1E-02
F29G9.4b	-0.76	-1.69	4.9E-02
ZC21.6b	-0.76	-1.69	3.3E-02
C54D10.4	-0.76	-1.69	3.5E-02
C54D10.8	-0.76	-1.69	3.9E-02
F23F12.3	-0.76	-1.69	3.4E-02
C26B2.3b.2	-0.76	-1.69	4.2E-02
F53B6.7	-0.76	-1.69	3.2E-02
T03F6.6	-0.76	-1.69	3.1E-02
C33A11.1.2	-0.76	-1.69	4.5E-02
Y32G9A.6	-0.76	-1.69	4.4E-02
C39D10.8b	-0.76	-1.69	3.4E-02
Y49F6B.1	-0.76	-1.69	4.0E-02
C31B8.7	-0.76	-1.69	3.2E-02
F48F7.8	-0.76	-1.70	4.1E-02
C49F8.3	-0.76	-1.70	3.0E-02
T28F3.4b.1	-0.76	-1.70	3.2E-02
F57H12.5	-0.76	-1.70	4.5E-02
F12D9.1a	-0.76	-1.70	3.4E-02
Y51A2D.8	-0.77	-1.70	4.6E-02
T08E11.4	-0.77	-1.70	3.8E-02
D2062.1	-0.77	-1.70	3.3E-02
Y69A2AR.4	-0.77	-1.70	3.8E-02
T20D4.9	-0.77	-1.70	3.2E-02
C26B2.3a.2	-0.77	-1.70	3.6E-02
ZC21.3	-0.77	-1.70	4.3E-02
K08E7.6	-0.77	-1.70	3.4E-02
C54D1.3	-0.77	-1.70	4.1E-02
Y57G11C.17	-0.77	-1.70	3.1E-02
K08F8.5a	-0.77	-1.70	3.4E-02
Y97E10C.1.1	-0.77	-1.70	3.8E-02
T04B2.7	-0.77	-1.70	3.1E-02
T19B4.1	-0.77	-1.70	3.2E-02
F07D3.3	-0.77	-1.71	4.2E-02
R13F6.6a	-0.77	-1.71	4.4E-02
T03F1.5	-0.77	-1.71	3.2E-02
T06A4.3a	-0.77	-1.71	3.2E-02
C52B9.1a	-0.77	-1.71	3.5E-02
F44F4.4	-0.77	-1.71	3.2E-02
C44H4.6	-0.77	-1.71	3.5E-02
Y38H8A.2a	-0.77	-1.71	3.4E-02
F32B5.7.1	-0.77	-1.71	2.9E-02
F32A5.8	-0.77	-1.71	3.0E-02

Supporting Table 2: Significant changes after 48h in H_2S			
gene	logFC (48hr)	fold-change	adj.P.Val (48hr)
Y55F3AM.10	-0.77	-1.71	3.2E-02
F11C1.5a	-0.77	-1.71	3.3E-02
T11F9.9	-0.77	-1.71	4.5E-02
W09B12.1.2	-0.77	-1.71	4.0E-02
Y113G7A.10	-0.77	-1.71	4.0E-02
F27D9.1a.1	-0.77	-1.71	3.4E-02
T21G5.5b	-0.77	-1.71	3.2E-02
F20C5.2a	-0.77	-1.71	4.4E-02
F54H5.3	-0.77	-1.71	3.6E-02
F14F7.3	-0.77	-1.71	2.9E-02
C51F7.1	-0.77	-1.71	2.9E-02
K09C8.7	-0.77	-1.71	2.8E-02
ZC8.3	-0.78	-1.71	3.5E-02
F32H2.5	-0.78	-1.71	3.4E-02
K09E4.4.1	-0.78	-1.71	4.3E-02
F46F2.2d.1	-0.78	-1.71	3.0E-02
F52F12.1a	-0.78	-1.71	3.3E-02
F23H12.5	-0.78	-1.71	3.5E-02
F53F4.8	-0.78	-1.71	2.8E-02
C08D8.2b	-0.78	-1.71	2.9E-02
F13B12.3	-0.78	-1.71	3.2E-02
R05H10.5	-0.78	-1.72	3.5E-02
F10D11.4	-0.78	-1.72	3.7E-02
ZK970.8	-0.78	-1.72	4.2E-02
T28F3.4a.1	-0.78	-1.72	3.0E-02
C39H7.1	-0.78	-1.72	3.8E-02
F21C3.6	-0.78	-1.72	2.9E-02
T12G3.2a.1	-0.78	-1.72	3.0E-02
T04D3.3	-0.78	-1.72	3.1E-02
Y53G8AR.7a	-0.78	-1.72	2.8E-02
Y7A9A.1	-0.78	-1.72	3.4E-02
C35B8.2a.1	-0.78	-1.72	3.0E-02
C49A9.8.1	-0.78	-1.72	3.8E-02
F55G7.1	-0.78	-1.72	3.1E-02
T21E8.3	-0.78	-1.72	2.7E-02
F10D2.11	-0.78	-1.72	4.2E-02
F59B10.6	-0.78	-1.72	3.5E-02
F26G1.5	-0.78	-1.72	3.5E-02
Y51H4A.28	-0.78	-1.72	2.8E-02
H10E21.5	-0.78	-1.72	3.2E-02
C08G5.5	-0.78	-1.72	3.6E-02
F43G9.6	-0.78	-1.72	3.4E-02
F42E11.1.1	-0.78	-1.72	3.6E-02
Y72A10A.1	-0.78	-1.72	4.6E-02
C07D10.4	-0.79	-1.72	3.2E-02
T06C12.10.1	-0.79	-1.72	3.0E-02
F11E6.1b	-0.79	-1.73	3.2E-02

Supporting Table 2: Significant changes after 48h in H_2S			
gene	logFC (48hr)	fold-change	adj.P.Val (48hr)
Y73B6BL.7	-0.79	-1.73	2.6E-02
ZK287.8b	-0.79	-1.73	4.6E-02
F39B3.2	-0.79	-1.73	3.4E-02
F55D12.6	-0.79	-1.73	4.3E-02
K08E7.7	-0.79	-1.73	4.0E-02
T25B6.7	-0.79	-1.73	2.7E-02
ZC477.7	-0.79	-1.73	3.2E-02
F14F7.4	-0.79	-1.73	2.8E-02
C07E3.3	-0.79	-1.73	2.7E-02
F14D12.2	-0.79	-1.73	2.6E-02
H23N18.5	-0.79	-1.73	2.5E-02
C49A1.2	-0.79	-1.73	2.6E-02
VY10G11R.1	-0.79	-1.73	2.7E-02
F11C1.6a.2	-0.79	-1.73	3.3E-02
F28B12.2e.2	-0.79	-1.73	3.2E-02
F32D8.12a	-0.79	-1.73	3.1E-02
F20B6.6	-0.79	-1.73	3.2E-02
C18E3.1	-0.79	-1.73	3.2E-02
F41B4.1	-0.79	-1.73	3.1E-02
C51E3.7a.2	-0.79	-1.73	2.8E-02
H10D18.5	-0.80	-1.74	2.6E-02
T04C4.1c	-0.80	-1.74	3.1E-02
F11E6.1a	-0.80	-1.74	3.4E-02
ZK218.1	-0.80	-1.74	2.5E-02
F36H12.8	-0.80	-1.74	3.3E-02
B0218.7	-0.80	-1.74	2.9E-02
Y71F9B.5a	-0.80	-1.74	3.1E-02
K05F1.7	-0.80	-1.74	3.5E-02
W05H12.1	-0.80	-1.74	2.6E-02
F52H3.7b.1	-0.80	-1.74	3.8E-02
ZK616.8	-0.80	-1.74	3.0E-02
T23F11.3.1	-0.80	-1.74	2.9E-02
Y116F11B.7	-0.80	-1.74	2.8E-02
ZK328.7a	-0.80	-1.74	2.9E-02
C30H6.2	-0.80	-1.74	2.6E-02
R05A10.6	-0.80	-1.74	2.5E-02
Y46D2A.1	-0.80	-1.74	2.6E-02
Y8G1A.1b	-0.80	-1.74	3.3E-02
Y38F1A.1	-0.80	-1.74	2.8E-02
F36A4.7	-0.80	-1.74	3.6E-02
F40G12.10	-0.80	-1.74	2.5E-02
F38A6.3a	-0.80	-1.74	3.1E-02
F54H12.1b	-0.80	-1.74	2.4E-02
Y51A2D.5	-0.80	-1.74	3.9E-02
R04E5.2	-0.80	-1.74	2.8E-02
T19C9.8	-0.80	-1.74	2.5E-02
F53B3.6	-0.80	-1.75	3.7E-02

Supporting Table 2: Significant changes after 48h in H_2S			
gene	logFC (48hr)	fold-change	adj.P.Val (48hr)
B0034.4	-0.80	-1.75	2.4E-02
F47B7.7	-0.80	-1.75	3.6E-02
C53C9.3a	-0.80	-1.75	2.4E-02
C33B4.3a	-0.80	-1.75	3.0E-02
C24G6.2a	-0.80	-1.75	3.0E-02
R08E3.4a	-0.80	-1.75	2.6E-02
T10A3.1b	-0.81	-1.75	2.7E-02
F21G4.1	-0.81	-1.75	3.9E-02
F02G3.1b	-0.81	-1.75	4.0E-02
R09A1.2	-0.81	-1.75	2.5E-02
F54D7.3	-0.81	-1.75	2.9E-02
T27A1.4	-0.81	-1.75	3.7E-02
Y53C10A.10	-0.81	-1.75	2.3E-02
F09B12.3	-0.81	-1.75	2.6E-02
C01C4.3b	-0.81	-1.75	3.1E-02
F42G2.3	-0.81	-1.75	2.6E-02
F53B6.4	-0.81	-1.75	2.4E-02
F46A8.6	-0.81	-1.75	3.4E-02
C28D4.5	-0.81	-1.75	3.6E-02
C11D2.6b	-0.81	-1.75	5.0E-02
Y48E1B.13a	-0.81	-1.75	2.8E-02
F18E9.5a	-0.81	-1.75	2.6E-02
F36A2.10	-0.81	-1.75	4.4E-02
ZK673.11a	-0.81	-1.75	2.2E-02
D2096.3.1	-0.81	-1.75	2.3E-02
Y71G12B.32	-0.81	-1.75	2.8E-02
C50E10.7	-0.81	-1.75	2.8E-02
F22E5.3	-0.81	-1.76	2.3E-02
C43F9.6	-0.81	-1.76	2.5E-02
DH11.5a	-0.81	-1.76	2.6E-02
T05A10.1h.1	-0.81	-1.76	2.4E-02
F10A3.4	-0.81	-1.76	2.7E-02
ZK858.2	-0.81	-1.76	2.2E-02
ZC247.2	-0.81	-1.76	3.0E-02
F40E10.4	-0.81	-1.76	2.5E-02
C01G6.1b.1	-0.81	-1.76	4.7E-02
F49B2.4	-0.81	-1.76	2.3E-02
C25F6.2a.2	-0.81	-1.76	3.1E-02
B0393.5	-0.81	-1.76	2.7E-02
Y45F10C.1	-0.81	-1.76	2.6E-02
Y105C5A.23	-0.81	-1.76	2.4E-02
T14G8.3b	-0.81	-1.76	2.6E-02
Y45F10B.8	-0.81	-1.76	2.4E-02
C17H11.6b	-0.81	-1.76	2.9E-02
F58F12.3	-0.82	-1.76	2.8E-02
F44F4.10	-0.82	-1.76	2.3E-02
H20J18.1b	-0.82	-1.76	3.6E-02

Supporting Table 2: Significant changes after 48h in H_2S			
gene	logFC (48hr)	fold-change	adj.P.Val (48hr)
F02G3.1a	-0.82	-1.76	4.4E-02
Y51H1A.1b	-0.82	-1.76	3.5E-02
C54D1.6.1	-0.82	-1.76	2.3E-02
M04G12.4b.1	-0.82	-1.76	2.5E-02
M03F4.6	-0.82	-1.76	2.7E-02
Y40H4A.2	-0.82	-1.76	2.6E-02
C26B2.3a.1	-0.82	-1.76	2.5E-02
F32A6.5	-0.82	-1.76	2.6E-02
R03D7.5	-0.82	-1.76	2.6E-02
C10G11.9	-0.82	-1.76	2.9E-02
C08F11.13.1	-0.82	-1.76	3.8E-02
Y119D3B.9	-0.82	-1.76	3.6E-02
F53F4.1	-0.82	-1.76	2.9E-02
C37A5.9	-0.82	-1.76	2.7E-02
M03F8.4	-0.82	-1.76	3.2E-02
R13A5.11	-0.82	-1.77	2.8E-02
B0034.3b	-0.82	-1.77	2.2E-02
C32H11.3	-0.82	-1.77	3.1E-02
Y54E2A.7	-0.82	-1.77	2.6E-02
Y43F8B.1c	-0.82	-1.77	4.0E-02
C46H11.6	-0.82	-1.77	2.4E-02
C04C11.2.2	-0.82	-1.77	2.6E-02
C07B5.3	-0.82	-1.77	2.8E-02
T27D12.2b	-0.82	-1.77	2.5E-02
F36H12.5	-0.82	-1.77	2.2E-02
F25B5.1b	-0.82	-1.77	2.6E-02
T21C12.1g	-0.82	-1.77	3.3E-02
F09C12.7	-0.82	-1.77	2.7E-02
K01A2.11d	-0.82	-1.77	4.0E-02
F49F1.14	-0.82	-1.77	3.8E-02
F49A5.3	-0.82	-1.77	2.2E-02
F09G8.8	-0.82	-1.77	3.2E-02
C15H7.3	-0.82	-1.77	2.2E-02
F57C12.5e	-0.82	-1.77	2.2E-02
Y47H9A.1	-0.82	-1.77	3.4E-02
M18.1	-0.82	-1.77	2.1E-02
C49A1.1	-0.82	-1.77	3.0E-02
ZK822.4	-0.82	-1.77	2.9E-02
K10B3.10	-0.82	-1.77	2.1E-02
F28F8.2.1	-0.83	-1.77	2.3E-02
ZC8.4b	-0.83	-1.77	3.2E-02
C11D2.6a	-0.83	-1.77	4.7E-02
Y54E2A.5	-0.83	-1.77	2.1E-02
F36D1.5	-0.83	-1.78	2.2E-02
C51E3.7a.1	-0.83	-1.78	2.1E-02
T07C4.9a	-0.83	-1.78	2.1E-02
F40F9.3	-0.83	-1.78	2.4E-02

Supporting Table 2: Significant changes after 48h in H_2S			
gene	logFC (48hr)	fold-change	adj.P.Val (48hr)
F48C1.7	-0.83	-1.78	2.5E-02
F15A8.5b	-0.83	-1.78	2.6E-02
M195.4	-0.83	-1.78	2.1E-02
C50F4.10	-0.83	-1.78	2.5E-02
F55A4.10	-0.83	-1.78	4.4E-02
C15A11.2	-0.83	-1.78	2.0E-02
F48F7.1	-0.83	-1.78	2.0E-02
C46F4.2.2	-0.83	-1.78	3.1E-02
B0348.2	-0.83	-1.78	2.7E-02
F52H3.7b.2	-0.83	-1.78	2.4E-02
C48E7.9	-0.83	-1.78	2.4E-02
Y45G5AM.1a.1	-0.83	-1.78	2.0E-02
T13B5.1.1	-0.83	-1.78	2.7E-02
K07E1.1	-0.83	-1.78	2.7E-02
F08B12.3b	-0.83	-1.78	3.2E-02
F11C3.3.2	-0.83	-1.78	2.5E-02
Y119C1B.3	-0.83	-1.78	2.1E-02
F13G3.1	-0.83	-1.78	2.0E-02
K03E6.5	-0.83	-1.78	2.0E-02
C33D3.1	-0.83	-1.78	2.8E-02
C10G6.1a	-0.83	-1.78	2.0E-02
Y46G5A.26b	-0.83	-1.78	2.4E-02
F11E6.1c	-0.83	-1.78	3.1E-02
C34E7.1.2	-0.83	-1.78	2.1E-02
T24F1.6a	-0.83	-1.78	2.1E-02
C04F12.6	-0.83	-1.78	3.0E-02
H01G02.3b	-0.83	-1.78	2.2E-02
ZC477.9b	-0.84	-1.78	2.6E-02
Y50E8A.2	-0.84	-1.78	2.0E-02
ZK1128.3	-0.84	-1.78	2.0E-02
C09H10.9	-0.84	-1.78	2.1E-02
Y47D9A.3	-0.84	-1.78	2.2E-02
T17H7.4e	-0.84	-1.79	3.1E-02
ZK892.4	-0.84	-1.79	1.9E-02
F59C6.8	-0.84	-1.79	2.9E-02
F56H9.3	-0.84	-1.79	3.5E-02
F36A2.4	-0.84	-1.79	3.4E-02
Y8G1A.1a	-0.84	-1.79	2.7E-02
F09C12.2	-0.84	-1.79	2.0E-02
F56F12.1	-0.84	-1.79	3.7E-02
C50B6.7	-0.84	-1.79	1.9E-02
R02F11.2	-0.84	-1.79	2.2E-02
F44D12.4	-0.84	-1.79	2.0E-02
F56B3.6	-0.84	-1.79	2.1E-02
Y22D7AR.1	-0.84	-1.79	2.9E-02
K09F6.6	-0.84	-1.79	3.0E-02
C44E12.3c	-0.84	-1.79	2.2E-02

Supporting Table 2: Significant changes after 48h in H_2S			
gene	logFC (48hr)	fold-change	adj.P.Val (48hr)
C25A11.4d	-0.84	-1.79	1.9E-02
C01C4.1	-0.84	-1.79	2.8E-02
C30A5.7a	-0.84	-1.79	3.4E-02
F44F4.5a	-0.84	-1.79	2.9E-02
K05F1.1	-0.84	-1.79	2.1E-02
T01H10.8	-0.84	-1.79	3.8E-02
F09F3.4	-0.84	-1.79	3.1E-02
F28B12.2a	-0.84	-1.79	2.3E-02
C49D10.10	-0.84	-1.79	2.3E-02
H34I24.1	-0.84	-1.79	2.6E-02
T28D9.7	-0.84	-1.79	2.0E-02
Y34D9A.8	-0.84	-1.80	1.9E-02
B0280.11	-0.84	-1.80	2.0E-02
C24A3.8	-0.85	-1.80	2.7E-02
T04F8.7	-0.85	-1.80	2.0E-02
C50E10.1	-0.85	-1.80	2.3E-02
R09H10.4	-0.85	-1.80	2.6E-02
R09A8.3.3	-0.85	-1.80	2.9E-02
Y46C8AL.4	-0.85	-1.80	2.1E-02
K08C9.1	-0.85	-1.80	1.9E-02
F10C2.3	-0.85	-1.80	2.9E-02
F58A4.7a.2	-0.85	-1.80	3.2E-02
ZK1005.1b	-0.85	-1.80	2.2E-02
F45H7.2b	-0.85	-1.80	2.1E-02
K02D7.1	-0.85	-1.80	1.8E-02
C30A5.7b	-0.85	-1.80	2.1E-02
R148.7	-0.85	-1.80	3.3E-02
T04B2.3a	-0.85	-1.80	2.0E-02
T06F4.1a	-0.85	-1.80	2.2E-02
ZK994.3	-0.85	-1.80	2.5E-02
K11H12.3	-0.85	-1.80	2.1E-02
T28C6.1	-0.85	-1.80	4.7E-02
ZC373.4	-0.85	-1.80	3.6E-02
K04G11.2	-0.85	-1.81	2.3E-02
ZK662.2	-0.85	-1.81	4.8E-02
C29F3.7	-0.85	-1.81	4.1E-02
F59A7.5b	-0.85	-1.81	2.1E-02
ZC374.2	-0.85	-1.81	1.9E-02
K02E10.7	-0.85	-1.81	1.8E-02
F11G11.14	-0.86	-1.81	4.3E-02
T16G1.5	-0.86	-1.81	3.2E-02
ZC123.4b	-0.86	-1.81	2.1E-02
Y47D3B.5a	-0.86	-1.81	1.7E-02
T21C12.1e	-0.86	-1.81	1.7E-02
C52A11.4a	-0.86	-1.81	3.0E-02
W04G3.4	-0.86	-1.81	1.8E-02
C37H5.8	-0.86	-1.81	2.0E-02

Supporting Table 2: Significant changes after 48h in H_2S			
gene	logFC (48hr)	fold-change	adj.P.Val (48hr)
D2092.1a	-0.86	-1.81	1.9E-02
F35C5.9	-0.86	-1.81	2.2E-02
Y59H11AM.2	-0.86	-1.81	2.0E-02
T23E7.2c	-0.86	-1.81	1.6E-02
F32B5.3	-0.86	-1.82	2.5E-02
T05A10.1e	-0.86	-1.82	1.7E-02
C44H9.4	-0.86	-1.82	3.5E-02
Y105C5A.24	-0.86	-1.82	1.9E-02
ZK938.1	-0.86	-1.82	2.2E-02
K03C7.1	-0.86	-1.82	2.7E-02
K09F6.9	-0.86	-1.82	2.4E-02
C39D10.8c	-0.86	-1.82	1.8E-02
ZC116.1	-0.86	-1.82	1.7E-02
F22F7.6	-0.86	-1.82	1.6E-02
C06H5.6	-0.86	-1.82	2.0E-02
T01C3.5	-0.86	-1.82	1.6E-02
F21F3.2	-0.87	-1.82	1.6E-02
H08M01.2a	-0.87	-1.82	2.4E-02
Y4C6B.3	-0.87	-1.82	1.9E-02
F08C6.1a.2	-0.87	-1.82	1.7E-02
F42G8.9	-0.87	-1.82	2.8E-02
F58H1.6	-0.87	-1.82	1.7E-02
F20D1.4	-0.87	-1.82	1.6E-02
M05D6.9	-0.87	-1.82	1.8E-02
Y67A10A.9	-0.87	-1.83	1.6E-02
Y23H5A.5d.1	-0.87	-1.83	1.9E-02
F37A8.4	-0.87	-1.83	2.8E-02
C47A10.6	-0.87	-1.83	2.1E-02
Y57G7A.5	-0.87	-1.83	2.7E-02
K10C2.5	-0.87	-1.83	1.5E-02
F47G6.2	-0.87	-1.83	1.7E-02
R07C3.1	-0.87	-1.83	2.6E-02
C15H7.4	-0.87	-1.83	2.2E-02
F14D7.11	-0.87	-1.83	1.9E-02
F54D1.5	-0.87	-1.83	3.3E-02
C14B4.2	-0.87	-1.83	1.8E-02
C25F6.2a.1	-0.87	-1.83	2.9E-02
F53B3.2.1	-0.87	-1.83	1.8E-02
Y53C10A.4	-0.87	-1.83	2.8E-02
ZK688.10	-0.87	-1.83	2.0E-02
ZC412.9	-0.87	-1.83	4.5E-02
T22F3.11b	-0.87	-1.83	1.6E-02
F13D2.1	-0.87	-1.83	2.3E-02
F33E2.4	-0.87	-1.83	2.5E-02
Y45F10B.3	-0.87	-1.83	2.1E-02
T02E9.1	-0.87	-1.83	2.2E-02
ZK930.4	-0.87	-1.83	1.9E-02

Supporting Table 2: Significant changes after 48h in H_2S			
gene	logFC (48hr)	fold-change	adj.P.Val (48hr)
F58A4.11	-0.88	-1.84	2.6E-02
ZC190.4	-0.88	-1.84	1.7E-02
F41D9.1	-0.88	-1.84	1.5E-02
F55G11.8	-0.88	-1.84	1.9E-02
T01D3.6a	-0.88	-1.84	1.5E-02
C27D8.1	-0.88	-1.84	1.5E-02
C13D9.9	-0.88	-1.84	1.6E-02
C54D10.5	-0.88	-1.84	1.6E-02
T01B10.4a.2	-0.88	-1.84	2.0E-02
T05B11.1	-0.88	-1.84	1.4E-02
T28F3.4b.2	-0.88	-1.84	1.7E-02
B0403.2	-0.88	-1.84	2.4E-02
ZK430.8	-0.88	-1.84	1.6E-02
Y37A1A.2	-0.88	-1.84	1.5E-02
F49E2.2a.2	-0.88	-1.84	1.6E-02
T07C4.9b.1	-0.88	-1.84	1.5E-02
T07F10.1b	-0.88	-1.84	2.1E-02
C04C11.2.1	-0.88	-1.84	1.4E-02
C25A11.4b	-0.88	-1.84	2.7E-02
C42D8.1	-0.88	-1.84	1.6E-02
B0252.5	-0.88	-1.84	2.4E-02
C33G3.4	-0.88	-1.84	2.1E-02
K08D10.8.1	-0.88	-1.84	1.9E-02
Y54G9A.4	-0.88	-1.84	2.8E-02
B0432.12	-0.88	-1.85	1.5E-02
F22A3.1	-0.88	-1.85	2.3E-02
Y69A2AR.31	-0.88	-1.85	1.8E-02
F13B9.6	-0.88	-1.85	1.4E-02
B0523.1	-0.89	-1.85	1.4E-02
C35D10.2	-0.89	-1.85	1.9E-02
T22F3.11a	-0.89	-1.85	1.6E-02
F16H6.2	-0.89	-1.85	2.3E-02
C52D10.12.1	-0.89	-1.85	1.6E-02
Y22D7AR.13.2	-0.89	-1.85	1.8E-02
T03G6.3.1	-0.89	-1.85	2.5E-02
Y23B4A.1	-0.89	-1.85	2.5E-02
F54G2.1a	-0.89	-1.85	1.4E-02
H20E11.1a	-0.89	-1.85	1.7E-02
C09E8.1b	-0.89	-1.85	1.7E-02
F32D8.12b.2	-0.89	-1.85	1.5E-02
F46F11.1b	-0.89	-1.85	2.4E-02
Y71H2B.7.2	-0.89	-1.85	2.7E-02
ZK593.9	-0.89	-1.85	1.5E-02
T19C3.1	-0.89	-1.85	1.8E-02
ZC581.6	-0.89	-1.85	1.9E-02
T23B7.2	-0.89	-1.85	2.5E-02
K04B12.1	-0.89	-1.85	1.4E-02

Supporting Table 2: Significant changes after 48h in H_2S			
gene	logFC (48hr)	fold-change	adj.P.Val (48hr)
W04E12.4	-0.89	-1.86	1.5E-02
Y87G2A.15	-0.89	-1.86	1.6E-02
F58B4.1b	-0.89	-1.86	1.7E-02
K08A2.5a.1	-0.89	-1.86	1.5E-02
T06C12.10.2	-0.89	-1.86	2.2E-02
F11A6.1b	-0.89	-1.86	1.5E-02
R04B5.6	-0.89	-1.86	1.9E-02
T01G6.8	-0.89	-1.86	1.3E-02
Y73B6BL.19	-0.89	-1.86	1.7E-02
T07C5.1b	-0.89	-1.86	1.5E-02
F07A5.7.2	-0.89	-1.86	1.5E-02
C04E6.5	-0.90	-1.86	1.4E-02
E03A3.4	-0.90	-1.86	3.5E-02
ZK1005.1a	-0.90	-1.86	1.3E-02
Y53C12C.1	-0.90	-1.86	1.4E-02
F35C11.3	-0.90	-1.86	1.4E-02
K08E5.2b.1	-0.90	-1.86	1.3E-02
C06B3.7	-0.90	-1.86	1.3E-02
M04C3.3	-0.90	-1.86	1.7E-02
ZC412.5	-0.90	-1.86	1.8E-02
ZK507.3	-0.90	-1.86	1.6E-02
D1044.2a	-0.90	-1.86	2.3E-02
F53F1.5	-0.90	-1.86	2.7E-02
F44A2.5b	-0.90	-1.86	1.3E-02
T27B7.4	-0.90	-1.86	2.1E-02
AC7.2a.1	-0.90	-1.86	1.3E-02
ZK892.1c	-0.90	-1.87	1.3E-02
B0280.17	-0.90	-1.87	1.9E-02
K11D12.10b	-0.90	-1.87	1.7E-02
T11B7.4e	-0.90	-1.87	1.3E-02
Y47G6A.19a	-0.90	-1.87	1.4E-02
W09B6.1b	-0.90	-1.87	1.5E-02
F47G6.4	-0.90	-1.87	1.3E-02
C44E12.3a	-0.90	-1.87	1.3E-02
C06A6.7	-0.90	-1.87	1.3E-02
B0222.5	-0.90	-1.87	1.4E-02
ZK488.7	-0.90	-1.87	2.1E-02
Y75B7B.1	-0.90	-1.87	1.3E-02
F53A9.6.1	-0.90	-1.87	1.4E-02
Y38E10A.25	-0.90	-1.87	1.3E-02
AC7.2b.1	-0.90	-1.87	1.3E-02
C09E9.2.2	-0.91	-1.87	3.9E-02
C34F11.6	-0.91	-1.87	1.5E-02
F11E6.3.1	-0.91	-1.88	1.6E-02
Y59E9AR.2	-0.91	-1.88	1.3E-02
C18G1.9	-0.91	-1.88	1.4E-02
C14A4.9.1	-0.91	-1.88	2.5E-02

Supporting Table 2: Significant changes after 48h in H_2S			
gene	logFC (48hr)	fold-change	adj.P.Val (48hr)
C27A7.5b	-0.91	-1.88	2.8E-02
Y105C5A.15	-0.91	-1.88	1.2E-02
C44H4.2	-0.91	-1.88	3.2E-02
C50H2.7	-0.91	-1.88	1.6E-02
ZC328.3a	-0.91	-1.88	1.3E-02
F15E6.6	-0.91	-1.88	1.9E-02
F11A1.2	-0.91	-1.88	1.3E-02
F32B6.5	-0.91	-1.88	1.5E-02
C06G1.5	-0.91	-1.88	1.2E-02
T25D10.5	-0.91	-1.88	1.2E-02
Y45F10B.13a	-0.91	-1.88	1.4E-02
H05G16.1	-0.91	-1.88	1.9E-02
F28E10.4	-0.91	-1.88	1.5E-02
E03H12.7	-0.91	-1.88	2.0E-02
T08B1.2b	-0.91	-1.88	1.3E-02
M03E7.1	-0.91	-1.88	1.3E-02
F38E1.3	-0.91	-1.88	1.3E-02
C49A1.3	-0.91	-1.88	1.3E-02
T14G11.3	-0.91	-1.88	1.7E-02
Y65B4BL.6	-0.91	-1.88	1.2E-02
F34D10.2.1	-0.91	-1.88	1.5E-02
T03G11.8	-0.91	-1.88	2.8E-02
F22A3.5	-0.91	-1.88	1.4E-02
Y38C1AA.5a	-0.91	-1.88	1.6E-02
F28A12.2	-0.91	-1.88	1.5E-02
T07A5.3	-0.91	-1.88	1.2E-02
C13D9.8	-0.91	-1.88	1.2E-02
Y57A10B.7	-0.91	-1.88	1.2E-02
K10F12.3b	-0.91	-1.88	1.4E-02
B0379.2	-0.91	-1.89	1.4E-02
K07C10.1	-0.91	-1.89	1.3E-02
T25B9.7	-0.91	-1.89	3.0E-02
F25H5.1b	-0.91	-1.89	2.8E-02
ZC581.2	-0.92	-1.89	1.2E-02
T23G5.5	-0.92	-1.89	1.4E-02
C46H11.10a	-0.92	-1.89	1.4E-02
C46H11.11b	-0.92	-1.89	1.4E-02
T07D4.2b	-0.92	-1.89	1.7E-02
F11D5.3b.1	-0.92	-1.89	1.4E-02
F42A10.7.1	-0.92	-1.89	1.9E-02
W09B6.1a.1	-0.92	-1.89	1.2E-02
C53A5.4	-0.92	-1.89	1.3E-02
F42G4.6	-0.92	-1.89	1.2E-02
F37A4.7a.1	-0.92	-1.89	1.7E-02
K11D9.3.3	-0.92	-1.89	1.3E-02
B0399.1b	-0.92	-1.89	2.4E-02
F10G8.2	-0.92	-1.89	1.4E-02

Supporting Table 2: Significant changes after 48h in H_2S			
gene	logFC (48hr)	fold-change	adj.P.Val (48hr)
C37C3.6c.1	-0.92	-1.89	1.3E-02
ZC477.2	-0.92	-1.89	1.2E-02
F44D12.1	-0.92	-1.89	2.0E-02
T07H8.4a	-0.92	-1.89	1.3E-02
C50F7.5	-0.92	-1.89	1.2E-02
Y22D7AR.8	-0.92	-1.89	1.4E-02
T23B3.5	-0.92	-1.90	1.2E-02
C44E12.3e	-0.92	-1.90	1.2E-02
K06A5.8a.2	-0.92	-1.90	1.3E-02
F13B9.1a	-0.92	-1.90	1.2E-02
F36D3.4	-0.92	-1.90	2.2E-02
F11E6.3.2	-0.92	-1.90	1.6E-02
C52B9.1b	-0.92	-1.90	1.3E-02
ZK270.2d	-0.92	-1.90	1.4E-02
C49A9.2	-0.92	-1.90	1.8E-02
C08F1.4a	-0.93	-1.90	2.5E-02
ZC190.1	-0.93	-1.90	1.5E-02
T05A10.1f	-0.93	-1.90	1.5E-02
K03H1.1	-0.93	-1.90	1.2E-02
Y59E9AL.2	-0.93	-1.90	1.3E-02
C14A6.5	-0.93	-1.90	1.1E-02
ZC581.7	-0.93	-1.90	1.1E-02
C47E12.12	-0.93	-1.90	1.1E-02
K11C4.1	-0.93	-1.90	1.3E-02
Y106G6G.3	-0.93	-1.90	1.1E-02
F47B3.5	-0.93	-1.90	1.2E-02
Y73F8A.19	-0.93	-1.90	1.1E-02
K10D11.5	-0.93	-1.90	1.3E-02
F12D9.1b	-0.93	-1.91	1.2E-02
F53B1.9	-0.93	-1.91	1.6E-02
K04H4.5	-0.93	-1.91	1.4E-02
Y57G11C.32	-0.93	-1.91	1.2E-02
F28B4.2	-0.93	-1.91	1.2E-02
C31H5.3	-0.93	-1.91	1.1E-02
F59F5.7	-0.93	-1.91	2.5E-02
F10E9.6a.1	-0.93	-1.91	1.1E-02
Y18D10A.8	-0.93	-1.91	1.2E-02
F45H7.6	-0.93	-1.91	1.5E-02
B0207.7	-0.93	-1.91	1.0E-02
Y6E2A.9b	-0.93	-1.91	1.3E-02
C02C6.2b	-0.93	-1.91	1.2E-02
ZK20.1	-0.93	-1.91	1.2E-02
Y75B8A.3	-0.93	-1.91	1.3E-02
W06F12.2b	-0.93	-1.91	1.0E-02
B0213.17.1	-0.93	-1.91	2.8E-02
C25A8.5	-0.94	-1.91	1.3E-02
C08E3.7	-0.94	-1.91	1.1E-02

Supporting Table 2: Significant changes after 48h in H_2S			
gene	logFC (48hr)	fold-change	adj.P.Val (48hr)
K12B6.1	-0.94	-1.91	1.2E-02
W03G11.4.1	-0.94	-1.91	2.6E-02
F55G1.15	-0.94	-1.91	1.6E-02
F44A2.5a.1	-0.94	-1.91	1.7E-02
M02H5.1	-0.94	-1.92	1.5E-02
ZK1225.5	-0.94	-1.92	2.6E-02
F46B6.10	-0.94	-1.92	1.0E-02
B0496.6	-0.94	-1.92	1.7E-02
C33A12.15	-0.94	-1.92	1.3E-02
F36H5.2c.1	-0.94	-1.92	2.4E-02
ZK177.2	-0.94	-1.92	1.1E-02
ZK418.2a	-0.94	-1.92	1.1E-02
Y53G8AM.5	-0.94	-1.92	1.3E-02
C10G11.8	-0.94	-1.92	1.0E-02
C15A11.3.1	-0.94	-1.92	1.5E-02
Y59H11AL.1a	-0.94	-1.92	1.2E-02
K10B4.3	-0.94	-1.92	1.1E-02
F35D11.2b	-0.94	-1.92	1.0E-02
F55A12.9d.1	-0.94	-1.92	9.9E-03
T01B7.8	-0.94	-1.92	1.5E-02
C07A9.8	-0.94	-1.92	1.2E-02
F20D6.8	-0.94	-1.92	1.1E-02
C27H6.1	-0.94	-1.92	1.2E-02
M04C3.1a	-0.94	-1.92	2.8E-02
C44B7.11	-0.94	-1.92	1.3E-02
C34F11.3a.1	-0.94	-1.92	1.0E-02
Y32F6A.2	-0.94	-1.92	1.3E-02
F16H11.5	-0.94	-1.92	9.8E-03
F36H2.5	-0.94	-1.92	1.0E-02
Y69A2AR.23	-0.94	-1.92	1.1E-02
C18C4.5b	-0.95	-1.93	1.6E-02
F36F12.7	-0.95	-1.93	1.3E-02
Y6E2A.9a	-0.95	-1.93	9.7E-03
F57B1.3	-0.95	-1.93	1.2E-02
C33D12.3	-0.95	-1.93	1.2E-02
T28H11.5	-0.95	-1.93	2.0E-02
Y57G11C.14	-0.95	-1.93	1.1E-02
ZK287.1.1	-0.95	-1.93	1.4E-02
ZK524.1.1	-0.95	-1.93	1.2E-02
T21B10.6	-0.95	-1.93	1.5E-02
K09F6.10	-0.95	-1.93	2.3E-02
ZK1010.9	-0.95	-1.93	1.3E-02
W01A11.3	-0.95	-1.93	1.4E-02
F55C12.2	-0.95	-1.93	1.1E-02
T22D1.11	-0.95	-1.93	1.5E-02
C52B9.9	-0.95	-1.93	1.3E-02
T21D12.9c.1	-0.95	-1.93	1.3E-02

Supporting Table 2: Significant changes after 48h in H_2S

gene	logFC (48hr)	fold-change	adj.P.Val (48hr)
F21F12.1	-0.95	-1.93	9.9E-03
C36C5.12	-0.95	-1.93	1.4E-02
ZK849.5	-0.95	-1.93	1.2E-02
R11F4.3	-0.95	-1.93	1.3E-02
F10E9.6c	-0.95	-1.93	1.0E-02
Y46G5A.22b	-0.95	-1.93	1.7E-02
T04H1.9	-0.95	-1.93	1.1E-02
M04G12.4a	-0.95	-1.93	2.1E-02
K03H1.9	-0.95	-1.93	1.1E-02
K03H1.5	-0.95	-1.94	1.8E-02
F27D9.8a	-0.95	-1.94	9.3E-03
C54H2.4	-0.95	-1.94	1.1E-02
Y39D8A.1a	-0.95	-1.94	9.2E-03
F16H6.7	-0.95	-1.94	9.5E-03
Y60A3A.24	-0.95	-1.94	1.0E-02
D2045.5	-0.96	-1.94	1.1E-02
K07E3.1	-0.96	-1.94	1.4E-02
Y57E12B.3	-0.96	-1.94	9.7E-03
F12F3.1d	-0.96	-1.94	9.6E-03
F02G3.1c	-0.96	-1.94	1.5E-02
Y43F8C.2.1	-0.96	-1.94	1.3E-02
C30H7.2a.4	-0.96	-1.94	9.0E-03
W05H9.3.2	-0.96	-1.94	9.2E-03
Y106G6D.4	-0.96	-1.94	1.1E-02
C33A12.6	-0.96	-1.94	1.1E-02
T11B7.3	-0.96	-1.94	1.8E-02
ZK353.4	-0.96	-1.94	9.9E-03
Y57G11C.5	-0.96	-1.94	1.0E-02
R166.5b	-0.96	-1.94	1.2E-02
C01G6.1b.2	-0.96	-1.94	1.5E-02
T01B10.4a.1	-0.96	-1.94	1.2E-02
Y105C5A.9a	-0.96	-1.94	8.9E-03
K09D9.11	-0.96	-1.94	1.8E-02
K10C2.1	-0.96	-1.94	8.8E-03
K06A4.6	-0.96	-1.94	1.1E-02
K01A11.4.1	-0.96	-1.94	1.1E-02
ZC328.3b	-0.96	-1.94	8.8E-03
F47B8.4	-0.96	-1.94	9.4E-03
C04G2.7	-0.96	-1.94	1.1E-02
F54F3.1	-0.96	-1.95	9.7E-03
F59G1.2	-0.96	-1.95	9.6E-03
F11A1.3b	-0.96	-1.95	1.1E-02
C17G10.4d	-0.96	-1.95	9.0E-03
F17A9.5	-0.96	-1.95	1.1E-02
ZK596.2	-0.96	-1.95	1.0E-02
Y46H3D.8	-0.96	-1.95	2.3E-02
C06G1.4.2	-0.96	-1.95	9.1E-03

**Supporting Table 2: Significant changes after 48h in H_2S**			
gene	logFC (48hr)	fold-change	adj.P.Val (48hr)
Y53F4B.28	-0.96	-1.95	9.2E-03
T05F1.8	-0.96	-1.95	8.6E-03
K09C6.2	-0.96	-1.95	1.1E-02
F46F5.14	-0.96	-1.95	3.9E-02
F41C6.1.1	-0.96	-1.95	8.7E-03
W08D2.3a	-0.97	-1.95	1.4E-02
K07A3.3b	-0.97	-1.95	9.5E-03
F35E8.8	-0.97	-1.95	1.3E-02
Y42A5A.1	-0.97	-1.95	1.3E-02
T11B7.4a	-0.97	-1.95	8.4E-03
K04G2.4	-0.97	-1.95	8.6E-03
W10D5.1	-0.97	-1.96	8.5E-03
F54H5.2	-0.97	-1.96	1.0E-02
F41C3.8a	-0.97	-1.96	1.1E-02
T12G3.2b.2	-0.97	-1.96	1.4E-02
F35H10.4.2	-0.97	-1.96	9.0E-03
Y48A6B.8	-0.97	-1.96	8.5E-03
Y102A11A.1	-0.97	-1.96	1.5E-02
F37A4.7d	-0.97	-1.96	9.9E-03
C49H3.12	-0.97	-1.96	1.4E-02
C01F6.6e.3	-0.97	-1.96	9.8E-03
C44E12.3b	-0.97	-1.96	1.0E-02
T01G5.1	-0.97	-1.96	1.0E-02
B0432.11	-0.97	-1.96	1.2E-02
F11C1.3	-0.97	-1.96	8.3E-03
C44E12.3d	-0.97	-1.96	8.8E-03
F21H7.5	-0.97	-1.96	1.1E-02
T27A3.4	-0.97	-1.96	9.7E-03
T07H6.1b.1	-0.97	-1.96	8.9E-03
C26F1.1a	-0.97	-1.96	8.4E-03
AC3.5	-0.97	-1.96	9.5E-03
C09F9.3	-0.97	-1.97	8.3E-03
F47B10.3	-0.98	-1.97	8.9E-03
T16G12.1	-0.98	-1.97	1.1E-02
T02B11.9	-0.98	-1.97	1.4E-02
F25E2.4	-0.98	-1.97	8.1E-03
F29G6.3b.1	-0.98	-1.97	9.7E-03
F15H9.1	-0.98	-1.97	1.7E-02
K03H9.3	-0.98	-1.97	8.2E-03
T07C5.1a	-0.98	-1.97	8.4E-03
C18B2.3	-0.98	-1.97	1.3E-02
C50E10.2	-0.98	-1.97	9.5E-03
C03A7.5	-0.98	-1.97	1.6E-02
T23F11.2	-0.98	-1.97	8.3E-03
K09E10.2	-0.98	-1.97	1.3E-02
R09E10.6	-0.98	-1.97	2.3E-02
Y43F8B.14	-0.98	-1.97	8.1E-03

Supporting Table 2: Significant changes after 48h in H_2S			
gene	logFC (48hr)	fold-change	adj.P.Val (48hr)
F13E6.2	-0.98	-1.97	9.7E-03
K09A11.2	-0.98	-1.97	1.1E-02
R01H10.3b	-0.98	-1.97	1.2E-02
F56C11.1	-0.98	-1.97	8.2E-03
K01C8.1.1	-0.98	-1.97	8.0E-03
C18H7.7	-0.98	-1.97	8.7E-03
F23A7.5	-0.98	-1.97	1.2E-02
C54D1.1	-0.98	-1.98	8.7E-03
C25F6.3	-0.98	-1.98	8.1E-03
Y49F6B.8	-0.98	-1.98	8.7E-03
R07C3.13	-0.98	-1.98	7.7E-03
C17H12.3	-0.98	-1.98	1.1E-02
ZK1193.5a	-0.99	-1.98	1.3E-02
Y49F6B.5	-0.99	-1.98	1.1E-02
F21H11.3.1	-0.99	-1.98	7.7E-03
C50F7.3	-0.99	-1.98	8.1E-03
F54G8.2	-0.99	-1.98	1.7E-02
T07H6.5	-0.99	-1.98	8.3E-03
Y95B8A.12b	-0.99	-1.98	1.5E-02
C33A11.4	-0.99	-1.98	9.8E-03
F41B5.3	-0.99	-1.98	8.5E-03
B0034.1	-0.99	-1.98	9.0E-03
C55C2.4	-0.99	-1.98	7.9E-03
B0496.3b	-0.99	-1.98	7.5E-03
T17H7.4f.2	-0.99	-1.98	1.3E-02
K02A2.3	-0.99	-1.98	1.7E-02
T11F9.12	-0.99	-1.98	1.4E-02
F56E3.3b	-0.99	-1.99	1.0E-02
Y23H5A.4	-0.99	-1.99	8.3E-03
F59B1.9	-0.99	-1.99	8.4E-03
F32A5.2a	-0.99	-1.99	7.3E-03
C45G9.9	-0.99	-1.99	1.1E-02
C52E4.7	-0.99	-1.99	8.7E-03
F58G1.6	-0.99	-1.99	9.5E-03
K12C11.4	-0.99	-1.99	7.1E-03
F37C4.8	-0.99	-1.99	8.2E-03
W03D8.10	-0.99	-1.99	8.1E-03
F45E4.6	-0.99	-1.99	7.2E-03
F53B2.8	-1.00	-1.99	1.2E-02
ZK673.11b	-1.00	-2.00	7.1E-03
H14N18.1a	-1.00	-2.00	9.7E-03
F57G4.5	-1.00	-2.00	2.7E-02
ZC123.1	-1.00	-2.00	1.3E-02
ZC482.1	-1.00	-2.00	9.0E-03
C38C6.5	-1.00	-2.00	7.2E-03
F15A8.5a	-1.00	-2.00	8.7E-03
K09E9.1.3	-1.00	-2.00	7.1E-03

Supporting Table 2: Significant changes after 48h in H_2S			
gene	logFC (48hr)	fold-change	adj.P.Val (48hr)
Y17G7B.11	-1.00	-2.00	9.1E-03
F58H12.1	-1.00	-2.00	9.6E-03
ZK1193.4	-1.00	-2.00	7.2E-03
F56C3.4	-1.00	-2.00	2.4E-02
F46F5.7	-1.00	-2.00	1.4E-02
F25H8.5b	-1.00	-2.00	7.3E-03
F58A4.7c	-1.00	-2.00	1.2E-02
F14D12.5	-1.00	-2.00	7.1E-03
C04E7.4	-1.00	-2.00	8.2E-03
B0511.3	-1.00	-2.00	8.1E-03
T25B9.2	-1.00	-2.00	7.2E-03
Y38E10A.19	-1.00	-2.00	1.3E-02
R90.5	-1.00	-2.00	8.3E-03
Y105E8A.25	-1.00	-2.00	1.0E-02
F42G10.1.1	-1.00	-2.00	8.8E-03
T28F4.3	-1.00	-2.00	7.1E-03
C05C12.1	-1.00	-2.00	7.7E-03
ZC449.5.1	-1.00	-2.00	8.7E-03
Y57G11C.24g	-1.00	-2.00	8.6E-03
C25G4.6	-1.00	-2.00	1.2E-02
M04C3.1b	-1.00	-2.00	1.4E-02
C37A2.3	-1.00	-2.00	8.4E-03
C07A4.3	-1.00	-2.00	7.1E-03
F54B3.2	-1.00	-2.00	8.3E-03
K06H7.8	-1.00	-2.00	8.6E-03
ZK688.2.2	-1.00	-2.00	8.3E-03
F49E2.5f.1	-1.00	-2.00	1.4E-02
B0513.6	-1.00	-2.01	7.4E-03
F46C5.6	-1.00	-2.01	7.8E-03
B0310.6	-1.00	-2.01	6.8E-03
F42G9.9c.1	-1.01	-2.01	9.2E-03
C25D7.2	-1.01	-2.01	8.4E-03
F23B2.7	-1.01	-2.01	9.8E-03
Y15E3A.4	-1.01	-2.01	7.7E-03
F41C3.2	-1.01	-2.01	9.9E-03
T13A10.11a.1	-1.01	-2.01	9.8E-03
Y105E8A.27	-1.01	-2.01	8.6E-03
W01D2.3	-1.01	-2.01	9.0E-03
Y53F4B.12	-1.01	-2.01	1.2E-02
Y57A10A.22	-1.01	-2.01	8.9E-03
F56E3.3a	-1.01	-2.01	8.5E-03
C06A8.6	-1.01	-2.01	9.6E-03
C41D7.2	-1.01	-2.01	6.7E-03
K12H4.1	-1.01	-2.01	7.4E-03
F22B8.4	-1.01	-2.01	1.2E-02
B0304.3	-1.01	-2.01	2.1E-02
ZK892.1a	-1.01	-2.01	7.7E-03

Supporting Table 2: Significant changes after 48h in H_2S			
gene	logFC (48hr)	fold-change	adj.P.Val (48hr)
F26E4.5	-1.01	-2.01	8.1E-03
C54D2.4a	-1.01	-2.01	6.6E-03
ZK688.2.1	-1.01	-2.01	9.9E-03
Y53F4B.36	-1.01	-2.01	7.2E-03
F08B12.1	-1.01	-2.02	9.0E-03
Y57G11A.3	-1.01	-2.02	1.2E-02
C31B8.1	-1.01	-2.02	1.4E-02
C47E8.7.1	-1.01	-2.02	6.4E-03
C34F11.9b	-1.01	-2.02	7.6E-03
F19H8.3	-1.01	-2.02	8.8E-03
Y37D8A.5	-1.01	-2.02	6.5E-03
Y66D12A.11	-1.01	-2.02	7.0E-03
C18B2.5a.1	-1.01	-2.02	8.2E-03
F31F4.14	-1.01	-2.02	8.3E-03
C15B12.4	-1.02	-2.02	6.7E-03
T17H7.4d	-1.02	-2.02	1.4E-02
C06B8.2b	-1.02	-2.02	1.4E-02
T19F4.1a	-1.02	-2.02	8.3E-03
B0218.5	-1.02	-2.02	7.2E-03
ZK546.7	-1.02	-2.02	1.4E-02
F15A8.5d	-1.02	-2.02	8.0E-03
T17H7.4a.1	-1.02	-2.02	1.0E-02
C16B8.1.1	-1.02	-2.02	8.4E-03
T21C12.1a	-1.02	-2.03	6.9E-03
Y37E11AL.4	-1.02	-2.03	6.2E-03
R01H10.3a.1	-1.02	-2.03	7.7E-03
F58G6.9	-1.02	-2.03	1.0E-02
F31B12.1c	-1.02	-2.03	7.2E-03
ZK337.1c.1	-1.02	-2.03	7.5E-03
Y43C5B.3	-1.02	-2.03	6.5E-03
Y48G1BR.1	-1.02	-2.03	1.2E-02
F16B4.12b	-1.02	-2.03	1.3E-02
C36F7.1	-1.02	-2.03	6.6E-03
ZC190.7	-1.02	-2.03	6.5E-03
Y43F4A.1a	-1.02	-2.03	7.5E-03
F55A12.9b	-1.02	-2.03	6.7E-03
F17C8.7	-1.02	-2.03	7.1E-03
K08E3.1	-1.02	-2.03	7.8E-03
F59E11.2	-1.02	-2.03	7.2E-03
C44H4.3	-1.02	-2.03	7.2E-03
C37C3.10	-1.02	-2.03	8.3E-03
D2005.6	-1.02	-2.03	1.4E-02
C31A11.5	-1.02	-2.03	7.2E-03
C34E7.1.1	-1.03	-2.04	6.6E-03
C04E12.10	-1.03	-2.04	1.0E-02
F23B2.11.1	-1.03	-2.04	6.4E-03
R01H2.2	-1.03	-2.04	1.4E-02

Supporting Table 2: Significant changes after 48h in H_2S			
gene	logFC (48hr)	fold-change	adj.P.Val (48hr)
T17H7.4f.1	-1.03	-2.04	9.9E-03
C35C5.5	-1.03	-2.04	6.3E-03
F48C11.1	-1.03	-2.04	9.2E-03
C38C6.2	-1.03	-2.04	8.5E-03
Y37D8A.23a	-1.03	-2.04	6.1E-03
B0399.1a	-1.03	-2.04	8.6E-03
T10H9.2	-1.03	-2.04	5.9E-03
F10F2.4	-1.03	-2.04	8.0E-03
C06A1.3	-1.03	-2.04	6.6E-03
Y59H11AR.2a.2	-1.03	-2.04	6.1E-03
AH6.3	-1.03	-2.04	1.6E-02
F58A3.2d	-1.03	-2.04	7.5E-03
T23F11.3.2	-1.03	-2.04	8.6E-03
ZC477.1	-1.03	-2.04	6.0E-03
M60.2.1	-1.03	-2.04	7.8E-03
K12G11.2	-1.03	-2.04	5.8E-03
C16D9.2a	-1.03	-2.04	7.8E-03
F33D11.7	-1.03	-2.04	5.6E-03
T09B4.7	-1.03	-2.05	5.8E-03
Y1H11.1	-1.03	-2.05	7.6E-03
C02F4.2a	-1.03	-2.05	7.3E-03
W10G11.17	-1.03	-2.05	1.0E-02
K09C4.8	-1.03	-2.05	6.3E-03
Y39B6A.4	-1.03	-2.05	1.4E-02
T08G11.2	-1.03	-2.05	7.0E-03
C17G10.4e	-1.04	-2.05	7.2E-03
C46H11.11a	-1.04	-2.05	1.0E-02
Y105C5A.6	-1.04	-2.05	4.5E-02
C24A8.3	-1.04	-2.05	2.1E-02
D1044.2b	-1.04	-2.05	6.7E-03
ZK484.2a	-1.04	-2.05	5.6E-03
F56F4.3	-1.04	-2.05	6.8E-03
C47D2.2	-1.04	-2.05	8.3E-03
Y60A3A.2	-1.04	-2.05	6.1E-03
F47B3.4	-1.04	-2.05	6.1E-03
F35C11.2	-1.04	-2.05	6.6E-03
T14D7.2	-1.04	-2.05	6.7E-03
F55H12.5	-1.04	-2.05	5.6E-03
Y47D3A.10	-1.04	-2.06	9.5E-03
F34D10.7.1	-1.04	-2.06	6.2E-03
C16C8.18	-1.04	-2.06	6.2E-03
ZK809.1	-1.04	-2.06	8.2E-03
C35A5.11	-1.04	-2.06	6.7E-03
Y74E4A.1a	-1.04	-2.06	9.2E-03
T05A10.1b	-1.04	-2.06	1.3E-02
F16C3.4	-1.04	-2.06	1.6E-02
C34D4.2	-1.04	-2.06	6.7E-03

Supporting Table 2: Significant changes after 48h in H_2S			
gene	logFC (48hr)	fold-change	adj.P.Val (48hr)
F56F3.4	-1.04	-2.06	5.3E-03
Y71F9B.5b.2	-1.04	-2.06	5.2E-03
F58B4.5	-1.05	-2.06	7.8E-03
C03C11.1	-1.05	-2.06	9.5E-03
F55H12.3	-1.05	-2.07	6.5E-03
Y11D7A.5	-1.05	-2.07	1.1E-02
B0344.2	-1.05	-2.07	1.1E-02
C46A5.1	-1.05	-2.07	1.8E-02
T17H7.4g.1	-1.05	-2.07	7.7E-03
F37E3.3	-1.05	-2.07	5.1E-03
C32C4.3	-1.05	-2.07	5.1E-03
K09E9.1.1	-1.05	-2.07	5.2E-03
T07H3.2	-1.05	-2.07	6.1E-03
F48C1.1	-1.05	-2.07	9.5E-03
T05A1.1a	-1.05	-2.07	5.8E-03
T17H7.4b.1	-1.05	-2.07	9.0E-03
ZK945.7	-1.05	-2.07	6.6E-03
T17H7.4l	-1.05	-2.07	8.7E-03
T06C10.6	-1.05	-2.07	7.7E-03
Y59E9AR.10	-1.05	-2.07	7.6E-03
ZK250.5b	-1.05	-2.08	1.1E-02
C38C10.3	-1.05	-2.08	5.5E-03
B0511.4	-1.06	-2.08	6.7E-03
ZC477.10	-1.06	-2.08	5.8E-03
F59C6.2	-1.06	-2.08	5.9E-03
W08E12.4	-1.06	-2.08	1.6E-02
T23F1.7a	-1.06	-2.08	4.9E-03
F09F7.5a	-1.06	-2.08	5.0E-03
R09E10.1	-1.06	-2.08	5.1E-03
K08D10.9	-1.06	-2.08	6.6E-03
Y75B8A.2b	-1.06	-2.08	1.1E-02
C52D10.3	-1.06	-2.08	4.9E-03
T13B5.1.2	-1.06	-2.08	6.4E-03
F32B6.11	-1.06	-2.08	6.3E-03
Y47D3A.31	-1.06	-2.08	5.7E-03
K07E3.7a.3	-1.06	-2.08	7.7E-03
B0350.2g.2	-1.06	-2.09	5.8E-03
T23G11.1	-1.06	-2.09	6.7E-03
T23H2.2	-1.06	-2.09	5.4E-03
Y18H1A.11	-1.06	-2.09	5.4E-03
C32E12.4	-1.06	-2.09	5.2E-03
W03D8.6.1	-1.06	-2.09	6.2E-03
F56D6.2	-1.06	-2.09	5.0E-03
C52D10.1	-1.06	-2.09	7.5E-03
C03F11.1	-1.06	-2.09	4.9E-03
T27A1.6	-1.06	-2.09	5.1E-03
D2024.3	-1.06	-2.09	1.0E-02

Supporting Table 2: Significant changes after 48h in H_2S			
gene	logFC (48hr)	fold-change	adj.P.Val (48hr)
R07H5.9	-1.06	-2.09	5.5E-03
Y47D3A.30	-1.06	-2.09	5.0E-03
F38B2.1b	-1.06	-2.09	5.1E-03
F17C8.1	-1.06	-2.09	5.8E-03
F27B3.2	-1.07	-2.09	5.3E-03
E03H4.8	-1.07	-2.09	7.1E-03
SSSD1.1	-1.07	-2.09	5.5E-03
C18H7.4	-1.07	-2.09	6.1E-03
F25H9.3	-1.07	-2.09	4.8E-03
C06E1.6	-1.07	-2.09	2.0E-02
Y113G7A.4a	-1.07	-2.10	4.7E-03
F54C9.4	-1.07	-2.10	1.2E-02
F47D12.7	-1.07	-2.10	5.4E-03
F14E5.3	-1.07	-2.10	9.8E-03
T12D8.9b	-1.07	-2.10	4.6E-03
F21F8.4.1	-1.07	-2.10	6.7E-03
Y43F8C.5	-1.07	-2.10	6.7E-03
ZC376.8	-1.07	-2.10	5.1E-03
EGAP1.3	-1.07	-2.10	5.0E-03
Y59H11AR.2b	-1.07	-2.10	9.9E-03
H10D12.2	-1.07	-2.10	4.7E-03
C47C12.6	-1.07	-2.10	4.6E-03
C37C3.6a.1	-1.07	-2.10	4.7E-03
F28F9.1.1	-1.07	-2.10	5.5E-03
R13A5.9	-1.07	-2.10	5.8E-03
F58A6.5	-1.07	-2.10	5.2E-03
F08B12.3a	-1.07	-2.10	4.5E-03
T22B7.1d	-1.07	-2.10	5.1E-03
F39C12.2e	-1.07	-2.10	4.7E-03
ZK337.1a.1	-1.07	-2.10	4.8E-03
F56D6.15	-1.07	-2.10	1.0E-02
K10G4.3	-1.07	-2.10	9.1E-03
F22D3.4	-1.07	-2.10	5.5E-03
F35B12.9	-1.07	-2.10	6.9E-03
F11C3.3.1	-1.07	-2.10	6.2E-03
K02F6.9	-1.07	-2.10	5.4E-03
F07A5.5	-1.07	-2.10	4.8E-03
F49E2.5g.1	-1.07	-2.10	6.0E-03
ZK265.3	-1.07	-2.10	8.3E-03
Y9C9A.8	-1.07	-2.10	4.6E-03
ZK1248.20	-1.07	-2.10	8.4E-03
T27D12.1.1	-1.07	-2.10	4.6E-03
Y77E11A.10	-1.07	-2.10	4.5E-03
C03G5.7	-1.07	-2.10	5.1E-03
T22A3.4a	-1.07	-2.11	5.7E-03
Y40H4A.1a.2	-1.07	-2.11	4.8E-03
ZK354.2a	-1.08	-2.11	4.6E-03

Supporting Table 2: Significant changes after 48h in H_2S			
gene	logFC (48hr)	fold-change	adj.P.Val (48hr)
F37H8.4	-1.08	-2.11	5.9E-03
F42H11.1	-1.08	-2.11	6.2E-03
W03D8.5	-1.08	-2.11	6.9E-03
ZC239.14	-1.08	-2.11	5.1E-03
ZK1193.2	-1.08	-2.11	6.1E-03
Y57G11C.21	-1.08	-2.11	6.4E-03
ZC116.3	-1.08	-2.11	1.4E-02
C05D11.4	-1.08	-2.11	5.1E-03
C41G7.6	-1.08	-2.11	6.0E-03
T28C12.3	-1.08	-2.11	4.4E-03
K02A4.2	-1.08	-2.11	9.5E-03
K11D12.10a	-1.08	-2.11	1.0E-02
F58E6.1a	-1.08	-2.11	1.0E-02
C02H7.3a	-1.08	-2.11	6.7E-03
AC3.10	-1.08	-2.11	4.5E-03
C29E6.2	-1.08	-2.11	7.2E-03
F55D12.2	-1.08	-2.11	1.8E-02
Y69E1A.2	-1.08	-2.11	4.2E-03
R05C11.3	-1.08	-2.12	5.2E-03
F30B5.7	-1.08	-2.12	9.9E-03
C41G11.3	-1.08	-2.12	4.9E-03
Y46C8AL.5.2	-1.08	-2.12	4.5E-03
D1065.4a	-1.08	-2.12	5.5E-03
T28C6.9	-1.08	-2.12	1.7E-02
R155.4	-1.08	-2.12	7.3E-03
F39C12.2a	-1.08	-2.12	4.8E-03
C40H5.5	-1.08	-2.12	4.7E-03
C04G2.9	-1.08	-2.12	5.2E-03
C10C5.2	-1.08	-2.12	2.0E-02
C05E4.14b	-1.09	-2.12	4.5E-03
T27F2.2	-1.09	-2.12	4.7E-03
C04G2.2	-1.09	-2.12	5.9E-03
C34B2.3	-1.09	-2.12	4.2E-03
T05A1.1b.1	-1.09	-2.12	4.8E-03
C33F10.12	-1.09	-2.13	4.9E-03
W01B6.2	-1.09	-2.13	4.1E-03
Y44A6D.5	-1.09	-2.13	5.2E-03
F41B5.6	-1.09	-2.13	1.5E-02
C14H10.3	-1.09	-2.13	4.8E-03
T27E7.1	-1.09	-2.13	5.8E-03
M01A8.2a	-1.09	-2.13	5.6E-03
W06B3.2b	-1.09	-2.13	4.3E-03
C31H1.5	-1.09	-2.13	4.3E-03
R01H10.3d	-1.09	-2.13	4.4E-03
F22E5.1	-1.09	-2.13	5.7E-03
R13H9.5	-1.09	-2.13	4.3E-03
F32D8.12b.1	-1.09	-2.13	4.3E-03

Supporting Table 2: Significant changes after 48h in H_2S			
gene	logFC (48hr)	fold-change	adj.P.Val (48hr)
T02B11.3b	-1.09	-2.13	8.3E-03
Y47G6A.13	-1.09	-2.13	9.7E-03
K07E3.7a.1	-1.09	-2.14	5.4E-03
Y34B4A.4b	-1.10	-2.14	5.9E-03
E02C12.8b	-1.10	-2.14	4.1E-03
T04B8.1	-1.10	-2.14	5.9E-03
F35E2.5	-1.10	-2.14	7.4E-03
T22A3.6	-1.10	-2.14	4.3E-03
F47B10.2	-1.10	-2.14	4.5E-03
K03B8.9.1	-1.10	-2.14	4.2E-03
F07A5.7.1	-1.10	-2.14	5.3E-03
T10H4.12	-1.10	-2.14	4.5E-03
B0511.11	-1.10	-2.14	4.0E-03
Y73B6BL.25	-1.10	-2.14	5.4E-03
T28H11.1	-1.10	-2.14	5.7E-03
C55B7.9.1	-1.10	-2.14	4.3E-03
F42A10.7.2	-1.10	-2.14	6.7E-03
Y56A3A.6.1	-1.10	-2.14	1.3E-02
C29E6.3	-1.10	-2.14	4.2E-03
F35D11.11a	-1.10	-2.14	1.2E-02
Y37E3.19	-1.10	-2.14	4.7E-03
F58B4.1a	-1.10	-2.15	4.6E-03
Y41E3.18	-1.10	-2.15	4.3E-03
C01F6.2	-1.10	-2.15	3.8E-03
ZK105.3	-1.10	-2.15	9.0E-03
ZK354.2b	-1.10	-2.15	4.0E-03
K10D6.3	-1.10	-2.15	5.0E-03
F31B12.1b	-1.10	-2.15	5.0E-03
K09E4.1	-1.10	-2.15	8.8E-03
K07A1.5	-1.11	-2.15	5.7E-03
T06E4.5	-1.11	-2.15	4.3E-03
D1014.1	-1.11	-2.16	3.8E-03
ZK849.1	-1.11	-2.16	4.6E-03
C12D12.1b	-1.11	-2.16	4.8E-03
Y48G1BM.9	-1.11	-2.16	5.3E-03
F36H12.14	-1.11	-2.16	7.2E-03
F23B12.4	-1.11	-2.16	4.9E-03
F18H3.3a.4	-1.11	-2.16	5.5E-03
T09B4.6	-1.11	-2.16	4.0E-03
C31A11.7	-1.11	-2.16	3.5E-03
DY3.5	-1.11	-2.16	1.8E-02
T22B7.1b.4	-1.11	-2.16	3.5E-03
C24G7.4	-1.11	-2.16	4.5E-03
Y71F9AL.2	-1.11	-2.16	4.0E-03
M04D8.6	-1.11	-2.16	6.6E-03
T21C12.1c.1	-1.11	-2.16	4.4E-03
F55D10.1	-1.11	-2.16	4.4E-03

Supporting Table 2: Significant changes after 48h in H_2S			
gene	logFC (48hr)	fold-change	adj.P.Val (48hr)
Y47H10A.1	-1.11	-2.16	5.9E-03
T05F1.5	-1.11	-2.16	6.2E-03
F34D10.7.2	-1.11	-2.17	4.5E-03
R102.10	-1.11	-2.17	3.7E-03
F18H3.3a.1	-1.11	-2.17	5.4E-03
F58E6.5	-1.12	-2.17	6.1E-03
F13E9.5	-1.12	-2.17	4.5E-03
C14A4.13	-1.12	-2.17	3.6E-03
F19H8.5	-1.12	-2.17	3.4E-03
D2092.7	-1.12	-2.17	1.4E-02
Y46C8AL.5.1	-1.12	-2.17	3.7E-03
C27D6.11	-1.12	-2.17	3.4E-03
C43E11.5	-1.12	-2.17	3.7E-03
F57A8.1.2	-1.12	-2.17	3.6E-03
ZK287.1.2	-1.12	-2.17	5.2E-03
W06H8.4	-1.12	-2.17	6.8E-03
Y51H4A.8	-1.12	-2.17	8.2E-03
F35E12.4	-1.12	-2.17	3.4E-03
T13C2.3a	-1.12	-2.17	3.8E-03
F57C12.5c	-1.12	-2.17	4.1E-03
C15A7.2	-1.12	-2.17	3.8E-03
Y71G12B.11b	-1.12	-2.17	4.0E-03
ZC416.8a	-1.12	-2.17	4.9E-03
E02C12.6	-1.12	-2.17	5.9E-03
C08E3.10b	-1.12	-2.17	1.0E-02
T19D12.2b	-1.12	-2.17	3.5E-03
F13B10.1b	-1.12	-2.17	5.7E-03
C04G2.8	-1.12	-2.18	9.2E-03
T23F1.7b	-1.12	-2.18	3.9E-03
T06G6.6	-1.12	-2.18	4.5E-03
T16D1.1	-1.12	-2.18	6.1E-03
H06H21.10b	-1.12	-2.18	4.3E-03
R13A1.4	-1.12	-2.18	6.0E-03
F31D5.3a	-1.12	-2.18	4.4E-03
EGAP798.1	-1.12	-2.18	5.1E-03
F35D11.11b	-1.12	-2.18	4.1E-03
C44B11.3.1	-1.12	-2.18	3.9E-03
F57C12.5a	-1.12	-2.18	3.9E-03
Y95B8A.10	-1.12	-2.18	6.2E-03
C13E3.1	-1.12	-2.18	5.1E-03
Y47G6A.5a	-1.12	-2.18	3.7E-03
C42D4.2	-1.12	-2.18	4.2E-03
F46G10.2	-1.12	-2.18	3.6E-03
C02F4.2b	-1.12	-2.18	5.9E-03
F35H10.4.3	-1.12	-2.18	4.2E-03
C16C10.12	-1.12	-2.18	4.6E-03
M7.7	-1.12	-2.18	3.3E-03

Supporting Table 2: Significant changes after 48h in H_2S			
gene	logFC (48hr)	fold-change	adj.P.Val (48hr)
Y54F10BM.3	-1.13	-2.18	3.5E-03
ZC239.3	-1.13	-2.18	3.6E-03
C42D8.8b.3	-1.13	-2.18	9.6E-03
F58A4.1	-1.13	-2.18	1.3E-02
T12D8.9a	-1.13	-2.18	3.4E-03
F35E12.9b	-1.13	-2.18	5.2E-03
F47A4.1b	-1.13	-2.18	3.2E-03
Y51A2B.4	-1.13	-2.18	3.3E-03
C36B1.10	-1.13	-2.18	3.2E-03
F36D1.6	-1.13	-2.19	1.2E-02
ZK783.5	-1.13	-2.19	3.6E-03
T13F3.8	-1.13	-2.19	7.1E-03
F25E5.10	-1.13	-2.19	1.8E-02
F52H2.6	-1.13	-2.19	3.3E-03
B0222.6	-1.13	-2.19	3.8E-03
C23H4.6	-1.13	-2.19	1.1E-02
ZK484.2b	-1.13	-2.19	3.1E-03
Y57G11C.20	-1.13	-2.19	3.3E-03
C33D9.3b	-1.13	-2.19	4.3E-03
F35D2.5c.1	-1.13	-2.19	3.5E-03
F57C12.5d.2	-1.13	-2.20	3.0E-03
R08A2.1	-1.14	-2.20	5.1E-03
Y39D8A.1c	-1.14	-2.20	3.9E-03
Y51A2D.19b	-1.14	-2.20	3.1E-03
W10G11.3	-1.14	-2.20	4.6E-03
F31E8.5	-1.14	-2.20	3.1E-03
C30A5.4	-1.14	-2.20	7.4E-03
C05E7.1	-1.14	-2.20	3.0E-03
Y18H1A.1	-1.14	-2.20	4.4E-03
D1037.5	-1.14	-2.21	3.1E-03
C38H2.2	-1.14	-2.21	3.0E-03
F28A12.1	-1.14	-2.21	4.1E-03
Y113G7A.11	-1.14	-2.21	3.2E-03
H27C11.1a	-1.14	-2.21	3.8E-03
T01C3.10	-1.14	-2.21	4.8E-03
Y67D8B.5	-1.14	-2.21	3.8E-03
C55C3.3	-1.14	-2.21	5.8E-03
H06H21.10a	-1.14	-2.21	3.6E-03
C39D10.3a	-1.14	-2.21	4.5E-03
F58G1.7	-1.14	-2.21	3.9E-03
M153.2	-1.14	-2.21	3.2E-03
W08D2.3b	-1.15	-2.21	3.4E-03
W01F3.3	-1.15	-2.21	1.5E-02
Y37E11AR.3a	-1.15	-2.21	4.1E-03
F42G4.2	-1.15	-2.21	3.4E-03
H24G06.1a	-1.15	-2.21	4.9E-03
F01G12.5b.1	-1.15	-2.22	3.3E-03

Supporting Table 2: Significant changes after 48h in H_2S			
gene	logFC (48hr)	fold-change	adj.P.Val (48hr)
ZK563.2	-1.15	-2.22	2.9E-03
R11E3.1	-1.15	-2.22	2.8E-03
T03F7.1.1	-1.15	-2.22	4.5E-03
K08F8.5b	-1.15	-2.22	3.3E-03
F46C8.6.2	-1.15	-2.22	3.1E-03
F20D6.1	-1.15	-2.22	2.8E-03
F54C1.9	-1.15	-2.22	4.8E-03
C44F1.5	-1.15	-2.22	3.4E-03
W06B3.2c	-1.15	-2.22	2.8E-03
F08G2.8	-1.15	-2.22	3.8E-03
F19B2.5	-1.15	-2.22	2.7E-03
ZK228.4	-1.15	-2.22	6.2E-03
Y43F8B.1a	-1.15	-2.22	6.1E-03
T21E12.5	-1.15	-2.23	2.6E-03
R12H7.1.1	-1.16	-2.23	3.5E-03
T20D4.11	-1.16	-2.23	3.6E-03
M01E11.7d	-1.16	-2.23	2.9E-03
T10H9.6a	-1.16	-2.24	1.1E-02
F54A3.4	-1.16	-2.24	5.6E-03
R10E8.6	-1.16	-2.24	2.6E-03
T03G11.3	-1.16	-2.24	2.7E-03
W05F2.4a	-1.16	-2.24	4.5E-03
C54D2.5f	-1.16	-2.24	2.8E-03
C53B4.2	-1.16	-2.24	4.5E-03
H35N03.1	-1.16	-2.24	2.9E-03
C33D12.2	-1.16	-2.24	4.9E-03
F07F6.6	-1.16	-2.24	2.6E-03
F53C3.1	-1.16	-2.24	3.2E-03
T06A4.3b	-1.16	-2.24	3.4E-03
ZK973.6	-1.16	-2.24	2.9E-03
W03C9.4	-1.16	-2.24	2.5E-03
Y46D2A.2	-1.16	-2.24	5.4E-03
Y40H4A.1a.1	-1.17	-2.24	2.8E-03
T07H8.4b	-1.17	-2.24	2.8E-03
Y54G2A.10b	-1.17	-2.24	3.0E-03
C08H9.1	-1.17	-2.24	3.2E-03
C47A4.3	-1.17	-2.24	3.0E-03
F18C12.1	-1.17	-2.24	3.9E-03
F12B6.1	-1.17	-2.25	2.5E-03
F42G9.9b.1	-1.17	-2.25	3.3E-03
H03A11.2	-1.17	-2.25	2.9E-03
R07E5.6	-1.17	-2.25	3.3E-03
B0244.10	-1.17	-2.25	3.9E-03
F46C8.6.1	-1.17	-2.25	5.4E-03
Y116A8A.4	-1.17	-2.25	3.6E-03
Y95B8A.12a	-1.17	-2.25	5.4E-03
H27C11.1b.1	-1.17	-2.25	2.5E-03

Supporting Table 2: Significant changes after 48h in H_2S			
gene	logFC (48hr)	fold-change	adj.P.Val (48hr)
F31F6.5	-1.17	-2.25	1.2E-02
ZC8.4d	-1.17	-2.25	2.8E-03
F28A10.3	-1.17	-2.25	2.6E-03
F40F8.8	-1.17	-2.25	2.6E-03
C48A7.1b	-1.17	-2.25	5.2E-03
T16A9.4	-1.17	-2.25	8.0E-03
T23F2.2a	-1.17	-2.25	2.4E-03
F56H1.1	-1.17	-2.25	3.6E-03
F12F3.1a.1	-1.17	-2.25	2.6E-03
T27D12.1.3	-1.17	-2.26	3.6E-03
C50C3.9a	-1.17	-2.26	5.4E-03
C35A5.4	-1.17	-2.26	2.3E-03
T05E11.7	-1.17	-2.26	2.5E-03
T06D8.3	-1.17	-2.26	3.7E-02
Y73F8A.5	-1.18	-2.26	2.6E-03
Y71D11A.5	-1.18	-2.26	2.4E-03
ZK354.6	-1.18	-2.26	2.7E-03
T06F4.2a	-1.18	-2.26	2.5E-03
T14E8.3d	-1.18	-2.26	3.0E-03
Y97E10AR.1	-1.18	-2.26	2.6E-03
T04B2.2	-1.18	-2.26	2.9E-03
C05D9.3	-1.18	-2.27	4.7E-03
ZK892.3	-1.18	-2.27	2.3E-03
Y46G5A.22a	-1.18	-2.27	2.7E-03
D1009.3b	-1.18	-2.27	2.4E-03
K10G6.4	-1.18	-2.27	3.1E-03
Y47G6A.5b	-1.18	-2.27	2.2E-03
F21C10.3a	-1.18	-2.27	2.2E-03
T14E8.3a	-1.18	-2.27	3.1E-03
T15B7.5	-1.18	-2.27	3.0E-03
K08D10.10	-1.18	-2.27	2.3E-03
B0350.2g.1	-1.18	-2.27	2.6E-03
ZC434.9b	-1.19	-2.27	2.5E-03
R01H10.3c	-1.19	-2.27	3.1E-03
F43C11.3	-1.19	-2.27	5.9E-03
T03G6.3.2	-1.19	-2.28	2.3E-03
ZK1053.6	-1.19	-2.28	2.3E-03
T01C8.7	-1.19	-2.28	2.5E-03
ZK783.1	-1.19	-2.28	2.7E-03
C24G7.2	-1.19	-2.28	2.7E-03
Y40H7A.2	-1.19	-2.28	3.1E-03
C17G10.3	-1.19	-2.28	2.2E-03
R13H7.2c	-1.19	-2.28	5.7E-03
F25H5.7	-1.19	-2.28	2.2E-03
F38A3.1	-1.19	-2.28	4.3E-03
C27B7.7	-1.19	-2.28	8.9E-03
Y49C4A.8a	-1.19	-2.28	2.8E-03

Supporting Table 2: Significant changes after 48h in H_2S			
gene	logFC (48hr)	fold-change	adj.P.Val (48hr)
C14C6.4	-1.19	-2.28	3.3E-03
F16F9.5	-1.19	-2.28	2.1E-03
F08A8.4	-1.19	-2.28	2.2E-03
Y59H11AR.2a.1	-1.19	-2.29	2.4E-03
F07A5.2	-1.19	-2.29	3.1E-03
F13H6.1	-1.19	-2.29	8.2E-03
Y76A2A.1	-1.19	-2.29	3.8E-03
Y39G10AR.16	-1.19	-2.29	2.7E-03
D1065.1	-1.19	-2.29	4.1E-03
Y57G11C.24a.1	-1.19	-2.29	2.3E-03
F43B10.2a	-1.19	-2.29	3.6E-03
T16G12.7	-1.19	-2.29	2.1E-03
Y69F12A.1	-1.20	-2.29	2.1E-03
Y46G5A.10	-1.20	-2.29	2.1E-03
C06B8.2a	-1.20	-2.29	4.8E-03
T22B7.1b.2	-1.20	-2.29	2.1E-03
F40F12.3	-1.20	-2.29	2.1E-03
F28A10.1	-1.20	-2.29	4.4E-03
R05G9.2a	-1.20	-2.29	3.0E-03
D1037.2	-1.20	-2.29	2.7E-03
B0379.7.1	-1.20	-2.30	2.9E-03
W04G3.3	-1.20	-2.30	3.8E-02
K08D8.4d	-1.20	-2.30	2.3E-03
ZK783.3	-1.20	-2.30	3.6E-03
Y44A6E.1a	-1.20	-2.30	2.5E-03
C27B7.6	-1.20	-2.30	4.7E-03
T21E3.3	-1.20	-2.30	2.1E-03
C29F3.5	-1.20	-2.30	3.9E-03
F57F4.1	-1.20	-2.30	2.0E-03
C33G3.1b.1	-1.20	-2.30	2.0E-03
F14F9.3	-1.20	-2.30	4.7E-03
F10E7.4	-1.20	-2.30	2.1E-03
T02E1.7	-1.20	-2.30	2.2E-03
F59A2.6	-1.20	-2.30	2.2E-03
Y95B8A.2	-1.20	-2.30	4.1E-03
T22B7.1a.2	-1.21	-2.31	2.1E-03
F58D2.2	-1.21	-2.31	2.0E-03
F21C10.8b	-1.21	-2.31	4.3E-03
ZC196.7	-1.21	-2.31	2.5E-03
C04E12.2.2	-1.21	-2.31	2.1E-03
M05B5.1	-1.21	-2.31	2.3E-03
K08F4.5	-1.21	-2.31	3.3E-03
C44F1.1	-1.21	-2.31	2.2E-03
C10C6.3	-1.21	-2.31	2.0E-03
F25F2.1b	-1.21	-2.31	2.2E-03
T26A5.1	-1.21	-2.31	1.9E-03
F47B3.6	-1.21	-2.32	2.5E-03

Supporting Table 2: Significant changes after 48h in H_2S			
gene	logFC (48hr)	fold-change	adj.P.Val (48hr)
C17H12.9	-1.21	-2.32	3.0E-03
ZK896.4	-1.21	-2.32	2.1E-03
T25B6.2	-1.21	-2.32	1.9E-03
F52E1.5	-1.21	-2.32	2.2E-03
Y73B6A.1	-1.21	-2.32	1.9E-03
F41D3.4	-1.21	-2.32	2.2E-03
K05F1.3	-1.21	-2.32	1.9E-03
F42E11.4.2	-1.21	-2.32	2.9E-03
C56C10.6	-1.22	-2.32	2.2E-03
C03H5.6	-1.22	-2.32	1.9E-03
C17H12.12	-1.22	-2.32	1.9E-03
F38B2.1a	-1.22	-2.32	2.0E-03
Y46G5A.26a	-1.22	-2.32	4.9E-03
B0554.5	-1.22	-2.32	2.0E-03
C29F5.2	-1.22	-2.32	2.1E-03
C55C2.3	-1.22	-2.32	2.2E-03
ZC416.8b	-1.22	-2.33	2.3E-03
F56H11.1c	-1.22	-2.33	2.5E-03
C24A1.3b	-1.22	-2.33	2.2E-03
F09A5.4e	-1.22	-2.33	5.4E-03
B0496.3c	-1.22	-2.33	2.0E-03
F33D4.6a	-1.22	-2.33	1.9E-02
W06D4.2	-1.22	-2.33	2.2E-03
F57F10.1b	-1.22	-2.33	1.8E-03
T05D4.5	-1.22	-2.33	1.8E-03
T04C12.7	-1.22	-2.33	2.3E-03
K02F6.3	-1.22	-2.33	3.1E-03
T23E7.2b	-1.22	-2.33	3.4E-03
T27A10.6.2	-1.22	-2.33	3.3E-03
Y97E10C.1.2	-1.22	-2.33	2.7E-03
F37B4.2.1	-1.22	-2.33	2.1E-03
Y51H7C.9	-1.22	-2.33	1.9E-03
F13D11.2a	-1.22	-2.33	2.3E-03
ZC410.4a	-1.22	-2.33	2.2E-03
F14D12.1b	-1.22	-2.34	3.7E-03
C29F5.3	-1.22	-2.34	1.9E-03
K08C9.2	-1.23	-2.34	3.2E-03
F18H3.3b	-1.23	-2.34	4.8E-03
F49E2.5e.1	-1.23	-2.34	2.8E-03
T13H5.1b	-1.23	-2.34	2.6E-03
K08D8.4a	-1.23	-2.34	2.1E-03
ZC250.1	-1.23	-2.34	1.7E-02
ZK484.7	-1.23	-2.34	2.1E-03
H14N18.1b.1	-1.23	-2.34	2.2E-03
F33D4.2g	-1.23	-2.34	1.7E-03
W03D8.9	-1.23	-2.34	2.7E-03
ZK849.4	-1.23	-2.34	2.7E-03

Supporting Table 2: Significant changes after 48h in H_2S

gene	logFC (48hr)	fold-change	adj.P.Val (48hr)
H27C11.1b.2	-1.23	-2.34	2.8E-03
F26A3.5	-1.23	-2.35	1.8E-03
F47A4.3a	-1.23	-2.35	1.9E-03
T20G5.5	-1.23	-2.35	5.0E-03
ZK6.8	-1.23	-2.35	2.1E-03
T22B7.1a.1	-1.23	-2.35	1.7E-03
F07A11.4	-1.23	-2.35	1.7E-03
Y39G10AR.6	-1.23	-2.35	1.8E-03
F49C12.10	-1.23	-2.35	2.5E-03
F31D5.3b	-1.23	-2.35	2.4E-03
F21C10.8a	-1.23	-2.35	5.0E-03
F36H2.3	-1.24	-2.36	1.9E-03
Y40H4A.1b.1	-1.24	-2.36	1.8E-03
B0545.3	-1.24	-2.36	2.1E-03
Y18D10A.6b.1	-1.24	-2.36	1.7E-03
K04E7.3	-1.24	-2.36	1.7E-03
R09G11.2d	-1.24	-2.36	4.2E-03
F31D5.3d	-1.24	-2.36	1.0E-02
C17C3.12b	-1.24	-2.36	3.1E-03
C52E12.2a.1	-1.24	-2.36	1.6E-03
Y71G12B.18	-1.24	-2.36	1.6E-03
T10A3.1a	-1.24	-2.36	2.1E-03
Y51H4A.7.1	-1.24	-2.36	2.2E-03
C50C3.9b	-1.24	-2.36	2.2E-03
W06G6.10	-1.24	-2.36	2.4E-03
Y70G10A.2	-1.24	-2.36	1.6E-03
ZK1290.3a	-1.24	-2.36	3.7E-03
Y51B9A.3	-1.24	-2.36	1.8E-03
F55F8.7	-1.24	-2.36	2.5E-03
M01A10.2a	-1.24	-2.37	2.7E-03
Y22D7AL.16	-1.24	-2.37	2.6E-03
F32H2.7	-1.24	-2.37	2.0E-03
R07E5.15	-1.24	-2.37	1.8E-03
R01H2.4	-1.24	-2.37	2.8E-03
T25G12.6	-1.24	-2.37	1.8E-03
F13B10.1a	-1.25	-2.37	2.6E-03
T07H6.1b.2	-1.25	-2.37	3.0E-03
T21G5.1	-1.25	-2.37	1.6E-03
C47E12.10	-1.25	-2.37	2.1E-03
Y6E2A.8	-1.25	-2.37	2.0E-03
4R79.1b	-1.25	-2.37	1.1E-02
C07H6.3.1	-1.25	-2.37	1.8E-03
C14F5.3a	-1.25	-2.38	1.6E-03
T07D3.4	-1.25	-2.38	3.1E-03
T06C10.3	-1.25	-2.38	1.6E-03
F21F8.4.2	-1.25	-2.38	1.6E-03
ZC239.4	-1.25	-2.38	4.1E-03

Supporting Table 2: Significant changes after 48h in H_2S			
gene	logFC (48hr)	fold-change	adj.P.Val (48hr)
F58G1.3	-1.25	-2.38	3.1E-03
F14D7.1	-1.25	-2.38	1.6E-03
C37C3.6a.2	-1.25	-2.38	1.6E-03
T26C5.2	-1.25	-2.38	7.1E-03
F43D9.1	-1.25	-2.38	1.8E-03
W04C9.1	-1.25	-2.38	1.7E-03
C09E9.2.1	-1.25	-2.38	6.0E-03
C02F12.7	-1.25	-2.38	2.2E-03
F28E10.1d	-1.25	-2.39	1.6E-03
F31D5.3c	-1.25	-2.39	1.8E-02
Y39A3CR.8	-1.25	-2.39	1.7E-03
C27D8.2	-1.25	-2.39	1.7E-03
ZC443.5	-1.25	-2.39	2.2E-03
K07D8.1.1	-1.26	-2.39	1.6E-03
T08B6.4	-1.26	-2.39	1.9E-03
F01G12.5a	-1.26	-2.39	1.6E-03
Y102A5C.21	-1.26	-2.39	3.9E-03
M176.7	-1.26	-2.39	2.0E-03
T19D12.2a.2	-1.26	-2.39	1.5E-03
F54G8.4	-1.26	-2.39	1.7E-03
Y57G11C.6	-1.26	-2.40	1.6E-03
F13B6.3.1	-1.26	-2.40	4.0E-03
T12G3.1.1	-1.26	-2.40	1.7E-03
C49C8.1	-1.26	-2.40	1.6E-03
Y48A6B.2	-1.26	-2.40	1.6E-03
F14H3.2	-1.26	-2.40	1.9E-03
F49E10.5	-1.26	-2.40	2.1E-03
F27E5.3	-1.26	-2.40	1.4E-03
F58A3.2a	-1.26	-2.40	2.4E-03
C08F8.6	-1.26	-2.40	1.4E-03
Y52D5A.1	-1.26	-2.40	1.9E-03
F32B6.10	-1.27	-2.40	2.4E-03
C23F12.1b	-1.27	-2.41	1.5E-03
C48E7.6	-1.27	-2.41	2.5E-03
F56D5.2	-1.27	-2.41	3.6E-03
ZK154.3	-1.27	-2.41	1.4E-03
K09C8.5	-1.27	-2.41	1.8E-03
F41C6.1.2	-1.27	-2.41	1.6E-03
K09C8.4	-1.27	-2.41	1.8E-03
C26H9A.2	-1.27	-2.41	1.5E-03
K01H12.2	-1.27	-2.41	1.4E-03
C23H3.7	-1.27	-2.42	2.1E-03
K01A11.4.2	-1.27	-2.42	1.5E-03
Y41D4B.15	-1.27	-2.42	1.3E-03
C01G6.1a.1	-1.27	-2.42	2.1E-03
W01F3.1a	-1.27	-2.42	1.5E-03
C05B5.6	-1.27	-2.42	3.0E-03

Supporting Table 2: Significant changes after 48h in H_2S			
gene	logFC (48hr)	fold-change	adj.P.Val (48hr)
Y59E9AL.3	-1.27	-2.42	1.4E-03
F35E12.8	-1.27	-2.42	1.5E-03
F31F4.1	-1.27	-2.42	1.6E-03
F35A5.4	-1.28	-2.42	1.3E-02
C33H5.16	-1.28	-2.42	2.4E-03
Y47D9A.5.1	-1.28	-2.42	2.1E-03
F13B6.1	-1.28	-2.42	1.6E-03
Y39D8A.1b	-1.28	-2.42	1.7E-03
Y19D10B.6	-1.28	-2.42	1.5E-03
C41C4.5.2	-1.28	-2.42	1.7E-03
F53B6.2b	-1.28	-2.42	1.4E-03
K09E4.4.2	-1.28	-2.43	2.0E-03
F15H10.7	-1.28	-2.43	1.4E-03
F18A12.5	-1.28	-2.43	4.2E-03
T25F10.3	-1.28	-2.43	1.6E-03
B0507.3b	-1.28	-2.43	2.0E-03
F21D12.3	-1.28	-2.43	1.6E-03
F53B2.5	-1.28	-2.43	1.4E-03
C18H9.7	-1.28	-2.43	1.4E-03
H23L24.2	-1.28	-2.43	1.3E-03
Y49C4A.8b.2	-1.28	-2.43	1.3E-03
C25F6.7b	-1.29	-2.44	1.6E-03
F56H11.3	-1.29	-2.44	1.3E-03
C05D2.3	-1.29	-2.44	1.6E-03
F18E9.4	-1.29	-2.44	4.9E-03
F09C3.1	-1.29	-2.44	1.7E-03
C05E4.14a	-1.29	-2.44	2.0E-03
R11G1.6b	-1.29	-2.44	1.4E-03
C44F1.2	-1.29	-2.44	2.0E-03
T07H6.1a	-1.29	-2.44	3.0E-03
W08D2.8	-1.29	-2.44	1.4E-03
ZK1055.7.1	-1.29	-2.44	1.3E-03
ZC504.2	-1.29	-2.45	1.6E-03
Y1A5A.1	-1.29	-2.45	1.3E-03
K04H4.1a	-1.29	-2.45	1.6E-03
C39B5.5	-1.29	-2.45	1.7E-03
R13H7.2a	-1.29	-2.45	3.6E-03
W03G9.5	-1.29	-2.45	2.2E-03
F09C12.8	-1.29	-2.45	2.2E-03
C54G4.2	-1.29	-2.45	1.5E-03
C55C3.8	-1.29	-2.45	1.3E-03
F52B10.1	-1.29	-2.45	1.2E-03
W06F12.3	-1.29	-2.45	1.4E-03
ZC373.6	-1.30	-2.45	2.3E-03
C29A12.4	-1.30	-2.46	1.5E-03
K06B4.12	-1.30	-2.46	1.8E-03
ZK1307.2	-1.30	-2.46	2.1E-03

Supporting Table 2: Significant changes after 48h in H_2S			
gene	logFC (48hr)	fold-change	adj.P.Val (48hr)
Y18D10A.6a	-1.30	-2.46	1.5E-03
C30F2.3	-1.30	-2.46	1.2E-03
C31H2.4	-1.30	-2.46	2.1E-03
Y116F11B.8	-1.30	-2.47	1.3E-03
C29F5.4b	-1.30	-2.47	1.2E-03
F59A7.9	-1.30	-2.47	1.7E-03
F56B6.4a	-1.30	-2.47	1.3E-03
T22B11.2	-1.30	-2.47	2.8E-03
T03G11.1	-1.30	-2.47	1.4E-03
F49E11.7	-1.30	-2.47	1.1E-03
ZK1290.6	-1.30	-2.47	1.2E-03
C10G8.5a	-1.30	-2.47	1.1E-03
W01B11.2	-1.30	-2.47	2.1E-03
F13A7.12	-1.30	-2.47	1.4E-03
C42D8.8a	-1.30	-2.47	1.4E-03
F47D12.1a	-1.31	-2.47	1.2E-03
ZK896.5.1	-1.31	-2.47	1.2E-03
K01H12.4	-1.31	-2.47	1.1E-03
ZK1225.4	-1.31	-2.47	1.5E-03
F38H4.4	-1.31	-2.48	1.3E-03
F16H6.5	-1.31	-2.48	2.3E-03
ZC101.2a	-1.31	-2.48	2.1E-03
C43G2.2	-1.31	-2.48	1.1E-03
F58D5.6	-1.31	-2.48	4.6E-03
C02F5.11	-1.31	-2.48	2.0E-03
T12A7.5	-1.31	-2.48	4.2E-03
F09A5.1	-1.31	-2.48	2.1E-03
Y39D8C.1	-1.31	-2.49	1.7E-03
R03D7.8	-1.31	-2.49	1.4E-03
F49E2.5a.1	-1.31	-2.49	1.3E-03
Y57G11C.23	-1.31	-2.49	1.6E-03
T27A10.6.1	-1.32	-2.49	1.2E-03
ZK180.2	-1.32	-2.49	2.8E-03
C01G12.7	-1.32	-2.49	1.9E-03
F53B1.4	-1.32	-2.49	3.7E-03
ZC101.2b	-1.32	-2.49	2.5E-03
B0416.1	-1.32	-2.49	1.0E-03
Y38E10A.17	-1.32	-2.49	1.8E-03
W06B3.2a	-1.32	-2.49	1.1E-03
D1054.9c	-1.32	-2.49	3.6E-03
F44F4.6	-1.32	-2.49	1.5E-03
C01G12.13	-1.32	-2.49	1.4E-03
R90.1	-1.32	-2.50	9.9E-04
Y37F4.1	-1.32	-2.50	2.8E-03
F36H12.9	-1.32	-2.50	1.4E-03
ZK1127.2	-1.32	-2.50	1.2E-03
ZC477.9c	-1.32	-2.50	1.3E-03

Supporting Table 2: Significant changes after 48h in H_2S			
gene	logFC (48hr)	fold-change	adj.P.Val (48hr)
Y49E10.9	-1.32	-2.50	1.4E-03
C34G6.6b	-1.32	-2.50	4.3E-03
F38A1.1	-1.32	-2.50	1.4E-03
W03G1.2	-1.32	-2.50	1.1E-03
C33G3.1a	-1.32	-2.50	1.7E-03
C24A11.2	-1.33	-2.51	1.3E-03
B0350.2b.1	-1.33	-2.51	1.7E-03
K08D8.4c	-1.33	-2.51	5.8E-03
F47D12.1c	-1.33	-2.51	1.3E-03
H42K12.3.2	-1.33	-2.51	1.8E-03
C31B8.4	-1.33	-2.51	1.4E-03
Y116A8C.24	-1.33	-2.51	9.6E-04
ZC47.4	-1.33	-2.51	1.5E-03
F20D6.5	-1.33	-2.51	1.3E-03
C32C4.1	-1.33	-2.51	1.2E-03
C03C10.6	-1.33	-2.52	1.4E-03
Y73C8C.4	-1.33	-2.52	1.3E-03
C53B4.8a	-1.33	-2.52	6.4E-03
F13D12.9	-1.33	-2.52	1.3E-03
Y47D3B.1	-1.34	-2.53	4.2E-03
B0361.11	-1.34	-2.53	9.3E-04
R04E5.10b	-1.34	-2.53	8.9E-04
T08H4.3	-1.34	-2.54	1.0E-03
K11H12.10	-1.34	-2.54	1.6E-03
C31B8.8	-1.34	-2.54	1.1E-03
F33D11.1	-1.34	-2.54	1.2E-03
Y50E8A.10	-1.34	-2.54	1.2E-03
F36H1.3	-1.34	-2.54	1.3E-03
C55C3.4	-1.34	-2.54	9.1E-04
C55C3.6	-1.34	-2.54	8.8E-04
F31F7.2	-1.34	-2.54	1.8E-03
F52H3.7a	-1.34	-2.54	9.8E-04
K02E7.6.1	-1.35	-2.54	8.5E-04
T10B9.8	-1.35	-2.54	1.9E-03
F07A5.6	-1.35	-2.54	5.7E-03
K08B5.1	-1.35	-2.54	1.2E-03
F58A3.2e	-1.35	-2.54	1.8E-03
F53C11.1	-1.35	-2.54	9.3E-04
W10G11.2	-1.35	-2.55	1.3E-03
R12E2.14	-1.35	-2.55	9.0E-04
C55C2.5a	-1.35	-2.55	1.6E-03
B0496.3a	-1.35	-2.55	1.2E-03
F47D12.1d	-1.35	-2.55	9.4E-04
F44F1.3	-1.35	-2.55	1.7E-03
T15H9.4	-1.35	-2.55	1.1E-03
C41C4.5.1	-1.35	-2.55	1.2E-03
Y39B6A.26	-1.35	-2.55	1.1E-03

Supporting Table 2: Significant changes after 48h in H_2S

gene	logFC (48hr)	fold-change	adj.P.Val (48hr)
R11G1.6a	-1.35	-2.55	8.4E-04
C56E6.6	-1.35	-2.56	4.2E-03
T02C5.5d.3	-1.35	-2.56	1.2E-03
T07H8.4c	-1.35	-2.56	1.3E-03
F28D1.8	-1.35	-2.56	8.2E-04
F19G12.7	-1.35	-2.56	1.8E-03
C17C3.12c.2	-1.35	-2.56	1.7E-03
Y49E10.10.1	-1.36	-2.56	1.1E-03
K07F5.6	-1.36	-2.56	8.1E-04
Y106G6E.1	-1.36	-2.56	1.0E-03
T13H5.1a	-1.36	-2.56	1.8E-03
F37C4.7	-1.36	-2.56	8.0E-04
Y44A6E.1b	-1.36	-2.57	1.4E-03
Y38F1A.3	-1.36	-2.57	3.4E-03
F53G12.6	-1.36	-2.57	7.8E-04
C30G7.3	-1.36	-2.57	1.3E-03
F35E12.7b	-1.36	-2.57	1.8E-03
B0495.10a	-1.36	-2.57	7.8E-04
F15B9.7	-1.36	-2.57	8.9E-04
C24H11.1	-1.36	-2.57	1.0E-03
K11C4.5	-1.36	-2.57	1.1E-03
F43B10.2b	-1.36	-2.57	1.2E-03
T13C2.2	-1.36	-2.57	1.9E-03
C17C3.12a	-1.36	-2.57	1.3E-03
K08E5.3a	-1.37	-2.58	9.3E-04
W01B6.5	-1.37	-2.58	9.4E-04
Y41G9A.1	-1.37	-2.58	9.1E-04
W03A5.1	-1.37	-2.58	1.5E-03
Y116A8C.38	-1.37	-2.58	9.3E-04
B0350.2e.1	-1.37	-2.58	1.5E-03
F47F6.4	-1.37	-2.58	1.0E-03
ZK1025.2	-1.37	-2.58	3.8E-03
F37C4.3	-1.37	-2.58	1.0E-03
C31H2.2	-1.37	-2.59	1.2E-03
Y97E10AR.2a	-1.37	-2.59	1.8E-03
K12B6.2	-1.37	-2.59	2.8E-03
F42A9.7	-1.37	-2.59	1.4E-03
T26H10.1	-1.37	-2.59	9.4E-04
C34E11.3	-1.37	-2.59	2.9E-03
C52E12.2a.2	-1.37	-2.59	1.9E-03
F47G6.3	-1.37	-2.59	1.2E-03
C09B9.4	-1.37	-2.59	7.9E-04
F44G3.10	-1.37	-2.59	3.6E-03
T09B9.2	-1.38	-2.59	9.1E-04
F28E10.1b.1	-1.38	-2.60	1.3E-03
M6.1c	-1.38	-2.60	6.0E-03
R12H7.1.2	-1.38	-2.60	7.7E-04

Supporting Table 2: Significant changes after 48h in H_2S			
gene	logFC (48hr)	fold-change	adj.P.Val (48hr)
F07F6.1	-1.38	-2.60	7.7E-04
F53B6.2a	-1.38	-2.60	8.5E-04
Y37A1B.11	-1.38	-2.60	7.4E-04
C36H8.1	-1.38	-2.60	1.2E-03
F18C5.4	-1.38	-2.60	9.5E-04
Y41D4B.16	-1.38	-2.61	7.4E-04
H32C10.1	-1.38	-2.61	8.2E-04
F57B9.9	-1.38	-2.61	7.6E-03
C08F8.4	-1.38	-2.61	6.8E-04
C23H4.2	-1.38	-2.61	1.3E-03
K06A1.2	-1.39	-2.61	8.2E-04
B0554.6	-1.39	-2.62	1.7E-03
H06A10.1	-1.39	-2.62	7.4E-04
R173.3	-1.39	-2.62	7.6E-04
B0222.8	-1.39	-2.62	7.6E-04
C10H11.6	-1.39	-2.62	9.3E-04
F37A4.5	-1.39	-2.62	9.0E-04
T23E7.2e	-1.39	-2.62	1.5E-03
F01D5.6	-1.39	-2.63	1.7E-03
C54G7.3b	-1.39	-2.63	1.5E-03
B0240.2	-1.39	-2.63	6.0E-03
Y37E11B.10a	-1.39	-2.63	7.5E-04
Y39B6A.25	-1.40	-2.63	1.3E-03
Y57G11A.1b	-1.40	-2.63	6.5E-04
K10G9.1	-1.40	-2.64	6.9E-04
Y69E1A.1	-1.40	-2.64	1.0E-03
F39D8.1a	-1.40	-2.64	1.3E-03
Y38A10A.2	-1.40	-2.64	1.6E-02
C46E1.2	-1.40	-2.64	7.2E-04
T19D12.5	-1.40	-2.64	7.3E-04
ZK524.2e	-1.40	-2.64	6.1E-04
C50H2.3	-1.40	-2.65	1.1E-03
F46C3.3	-1.41	-2.65	6.0E-04
F46A8.10	-1.41	-2.65	6.5E-04
K09A9.6	-1.41	-2.65	6.4E-04
K09B11.5a	-1.41	-2.65	7.3E-04
C42D8.5b.3	-1.41	-2.65	3.8E-03
T28F3.5	-1.41	-2.65	2.3E-03
C50C3.2	-1.41	-2.65	7.0E-04
F57B9.8	-1.41	-2.65	6.4E-04
F55G11.7	-1.41	-2.65	6.1E-04
T03F6.4	-1.41	-2.65	1.7E-03
R04E5.10a	-1.41	-2.65	6.0E-04
F37B4.13	-1.41	-2.65	1.6E-03
F28F8.2.2	-1.41	-2.66	6.0E-04
F44D12.8	-1.41	-2.66	6.0E-04
T16A9.5	-1.41	-2.66	1.2E-03

Supporting Table 2: Significant changes after 48h in H_2S

gene	logFC (48hr)	fold-change	adj.P.Val (48hr)
F43G6.6	-1.41	-2.66	9.6E-04
Y38E10A.18	-1.41	-2.66	9.3E-04
F45H11.4	-1.41	-2.66	6.1E-04
F45H7.4.1	-1.41	-2.66	6.1E-04
ZK1151.1a	-1.41	-2.66	6.2E-04
R04D3.1	-1.41	-2.66	6.9E-04
K08H10.3a	-1.42	-2.67	2.8E-03
F32A11.3	-1.42	-2.67	1.1E-03
K08D8.4b	-1.42	-2.67	6.1E-04
T12G3.1.2	-1.42	-2.67	8.4E-04
ZK666.8	-1.42	-2.67	6.4E-04
C55C2.5c	-1.42	-2.67	7.5E-04
Y65B4A.9	-1.42	-2.68	1.4E-03
T04A8.3	-1.42	-2.68	5.8E-04
Y50E8A.16	-1.42	-2.68	6.7E-04
C17D12.6	-1.42	-2.68	6.9E-04
C47A10.1	-1.42	-2.68	7.8E-04
H32C10.3	-1.42	-2.68	1.3E-03
C50H2.12	-1.42	-2.68	1.5E-03
F35D2.5a	-1.42	-2.68	5.7E-04
C48B6.4	-1.43	-2.69	1.2E-03
R52.2	-1.43	-2.69	1.0E-03
F35F10.14	-1.43	-2.69	1.1E-03
F44D12.6	-1.43	-2.69	6.7E-04
C17H12.5	-1.43	-2.69	7.6E-04
K10H10.10	-1.43	-2.69	5.4E-04
F36H12.13	-1.43	-2.69	1.2E-03
M01F1.7	-1.43	-2.69	5.3E-04
B0218.1b.1	-1.43	-2.69	9.5E-04
Y46G5A.30	-1.43	-2.70	5.7E-04
F35A5.1	-1.43	-2.70	1.4E-03
B0205.10.1	-1.43	-2.70	5.5E-04
Y57G7A.6	-1.43	-2.70	5.3E-04
C55B7.10	-1.44	-2.70	5.1E-04
T22H6.6	-1.44	-2.71	8.4E-04
K08E5.3b	-1.44	-2.71	5.4E-04
R09E10.4	-1.44	-2.71	6.3E-04
Y37E11B.10c	-1.44	-2.71	2.2E-03
T28D6.2	-1.44	-2.71	6.9E-04
ZK829.2	-1.44	-2.71	5.3E-04
T19D12.1	-1.44	-2.71	6.1E-04
B0303.11	-1.44	-2.71	5.1E-04
Y55F3C.3	-1.44	-2.71	6.7E-04
F19B6.3	-1.44	-2.72	6.8E-04
Y73F8A.20	-1.44	-2.72	5.3E-04
Y47D7A.6	-1.44	-2.72	6.7E-04
R01E6.4	-1.44	-2.72	6.5E-04

Supporting Table 2: Significant changes after 48h in H_2S			
gene	logFC (48hr)	fold-change	adj.P.Val (48hr)
F47B3.1	-1.44	-2.72	1.6E-03
F49C5.3	-1.44	-2.72	7.8E-04
K08B12.2b	-1.44	-2.72	1.0E-03
B0454.6	-1.45	-2.72	5.5E-04
F59D6.2	-1.45	-2.73	1.4E-03
C30D11.3	-1.45	-2.73	5.9E-04
ZC21.2a	-1.45	-2.73	5.8E-04
T13H10.1	-1.45	-2.73	5.5E-04
ZC21.9	-1.45	-2.73	3.8E-03
R05D7.2	-1.45	-2.73	6.3E-04
C36F7.5	-1.45	-2.73	6.0E-04
C35E7.10b	-1.45	-2.73	5.5E-04
ZK622.1	-1.45	-2.73	5.1E-04
K02F6.2	-1.45	-2.73	4.7E-04
C06B3.8.1	-1.45	-2.73	4.7E-04
F26A1.3	-1.45	-2.74	1.7E-03
K08A2.5c.1	-1.45	-2.74	5.3E-04
F09F9.4	-1.45	-2.74	4.6E-04
F17E5.1a	-1.45	-2.74	5.9E-04
C13D9.1	-1.45	-2.74	5.1E-04
F13B6.3.2	-1.46	-2.75	1.3E-03
ZK180.1	-1.46	-2.75	4.5E-04
Y53H1C.3	-1.46	-2.75	7.5E-04
B0350.2c.1	-1.46	-2.75	9.4E-04
K02E7.6.2	-1.46	-2.75	5.3E-04
C18H2.5	-1.46	-2.76	1.8E-03
F56A11.6	-1.46	-2.76	7.6E-04
T18D3.4	-1.46	-2.76	1.8E-03
Y119D3B.17	-1.46	-2.76	6.5E-04
ZK1151.1d	-1.46	-2.76	1.3E-03
Y69E1A.4	-1.47	-2.76	8.6E-04
T02C5.5d.2	-1.47	-2.76	1.1E-03
F45E4.3b.1	-1.47	-2.76	1.1E-03
W01B6.8	-1.47	-2.76	1.7E-03
F53B7.5	-1.47	-2.77	4.4E-04
Y73F8A.30	-1.47	-2.77	7.6E-04
R10H10.2	-1.47	-2.77	4.7E-04
F23B12.1	-1.47	-2.77	4.1E-04
R13G10.4	-1.47	-2.77	1.4E-03
D1054.9a	-1.47	-2.77	1.0E-03
F33A8.7	-1.47	-2.77	7.7E-04
F10G8.8	-1.47	-2.77	9.5E-04
ZK896.1	-1.47	-2.78	6.6E-04
T25B9.5	-1.47	-2.78	5.9E-04
C18D1.2	-1.47	-2.78	5.5E-04
H08M01.1	-1.47	-2.78	8.0E-04
F09B9.2b	-1.47	-2.78	1.3E-03

Supporting Table 2: Significant changes after 48h in H_2S			
gene	logFC (48hr)	fold-change	adj.P.Val (48hr)
K07A3.3a	-1.47	-2.78	5.3E-04
E04D5.3	-1.48	-2.78	5.9E-04
C33G3.3	-1.48	-2.78	3.5E-03
T06D10.1	-1.48	-2.78	4.1E-04
F19B6.4	-1.48	-2.78	9.4E-04
F22B3.8	-1.48	-2.79	5.3E-04
R08C7.6	-1.48	-2.79	4.0E-04
F29G6.1	-1.48	-2.79	4.3E-04
C54D10.1	-1.48	-2.79	9.8E-04
K08B12.2a	-1.48	-2.79	3.8E-04
C25D7.5	-1.48	-2.79	6.5E-04
H42K12.3.1	-1.48	-2.80	3.6E-03
T13F2.9	-1.48	-2.80	2.1E-03
F54C1.1	-1.48	-2.80	4.5E-04
R12E2.15	-1.49	-2.80	4.6E-04
C08E8.4	-1.49	-2.80	1.6E-03
F07C3.1	-1.49	-2.80	7.2E-04
F13H8.5	-1.49	-2.81	1.8E-03
F29C12.1a.1	-1.49	-2.81	3.5E-03
C23F12.1a	-1.49	-2.81	5.1E-04
T01B11.4	-1.49	-2.81	4.1E-04
W09C3.6	-1.49	-2.81	6.0E-04
M60.5	-1.49	-2.81	4.1E-04
M01H9.2	-1.49	-2.81	7.6E-04
F14D12.4a	-1.49	-2.81	6.8E-04
C34H4.2	-1.49	-2.81	6.0E-04
Y37E11B.10b	-1.49	-2.81	1.2E-03
Y71G12B.11a	-1.49	-2.82	1.0E-03
C09B9.3	-1.49	-2.82	4.8E-04
T25B9.6	-1.49	-2.82	4.2E-04
K08C7.3b	-1.50	-2.82	3.6E-04
C09D1.1b	-1.50	-2.82	3.8E-04
Y54G2A.24b	-1.50	-2.82	3.8E-04
K09C8.1	-1.50	-2.82	3.7E-04
Y46G5A.20	-1.50	-2.82	1.1E-03
F47C12.4	-1.50	-2.83	3.4E-04
F25F2.1a	-1.50	-2.83	4.3E-04
F45E4.3b.2	-1.51	-2.84	5.1E-04
B0228.4a	-1.51	-2.84	6.4E-04
F27C8.5	-1.51	-2.84	3.4E-04
Y52D5A.2	-1.51	-2.84	8.8E-04
F18A12.4	-1.51	-2.84	7.4E-04
C09D4.3	-1.51	-2.85	3.4E-04
ZK524.2a	-1.51	-2.85	3.5E-04
F07E5.8	-1.51	-2.85	3.6E-04
F13D11.2b	-1.51	-2.86	5.0E-04
F35G2.5	-1.51	-2.86	4.7E-04

Supporting Table 2: Significant changes after 48h in H_2S			
gene	logFC (48hr)	fold-change	adj.P.Val (48hr)
Y116A8C.23	-1.51	-2.86	8.2E-04
T16H12.6	-1.52	-2.87	3.2E-04
T16A1.2	-1.52	-2.87	9.7E-04
F32B4.2	-1.52	-2.87	3.3E-04
K07F5.4a	-1.52	-2.87	6.8E-04
B0280.12a	-1.52	-2.87	4.1E-04
F14D12.1a	-1.52	-2.88	1.0E-03
M28.4	-1.52	-2.88	3.6E-04
F49E10.1	-1.53	-2.89	4.0E-04
F52H3.6	-1.53	-2.89	7.4E-04
K10D11.1	-1.53	-2.89	3.6E-04
Y70G10A.3	-1.53	-2.89	3.3E-04
C32E12.1	-1.53	-2.89	2.9E-04
F54H12.5	-1.53	-2.90	3.2E-04
Y47D7A.11	-1.53	-2.90	7.9E-04
Y69H2.10a	-1.54	-2.91	4.1E-04
ZK1053.2	-1.54	-2.91	3.4E-04
C35E7.10a	-1.54	-2.91	3.3E-04
W10G11.1	-1.54	-2.91	4.0E-04
T13G4.3	-1.54	-2.91	2.9E-04
F54F2.1	-1.54	-2.92	6.0E-04
C08F1.5a	-1.54	-2.92	2.8E-04
B0554.7	-1.55	-2.92	3.2E-04
K09C6.7	-1.55	-2.92	3.9E-04
T20H4.1	-1.55	-2.92	3.1E-04
F38A5.10	-1.55	-2.92	2.7E-04
ZC21.2b	-1.55	-2.92	3.0E-04
F52F10.3	-1.55	-2.93	2.8E-04
C08E3.9	-1.55	-2.93	1.3E-03
Y46C8AL.1	-1.55	-2.93	3.4E-04
T27A3.5	-1.55	-2.93	2.6E-04
F12A10.4	-1.55	-2.93	3.4E-04
T20B6.1	-1.56	-2.94	3.1E-04
F36G9.3	-1.56	-2.94	3.4E-04
R08C7.5	-1.56	-2.94	2.7E-04
T02C5.5b	-1.56	-2.94	6.0E-04
ZK354.8	-1.56	-2.95	3.6E-04
W02A2.3	-1.56	-2.95	9.5E-03
F15G9.3	-1.56	-2.95	7.1E-03
Y18H1A.9	-1.56	-2.95	5.8E-04
T20D4.12	-1.56	-2.95	1.0E-03
F59D6.3	-1.56	-2.96	3.0E-04
R02F2.5	-1.56	-2.96	2.5E-04
T07H8.6	-1.56	-2.96	5.1E-04
B0019.1	-1.57	-2.96	4.1E-04
ZC513.10	-1.57	-2.96	7.0E-04
M28.9.1	-1.57	-2.97	2.8E-04

Supporting Table 2: Significant changes after 48h in H_2S			
gene	logFC (48hr)	fold-change	adj.P.Val (48hr)
F35E12.9a	-1.57	-2.97	7.5E-04
C30B5.3	-1.57	-2.97	2.9E-04
H11E01.3	-1.57	-2.98	3.4E-04
Y37E11AR.7	-1.57	-2.98	5.2E-04
B0491.8a	-1.58	-2.98	3.1E-04
T02E9.3	-1.58	-2.98	2.6E-04
F26F2.1	-1.58	-2.99	2.5E-04
K09E4.6	-1.58	-2.99	2.3E-04
H24G06.1b	-1.58	-2.99	5.3E-04
W02B12.7	-1.58	-3.00	3.0E-04
R09E10.9	-1.58	-3.00	8.8E-04
T22B3.2a	-1.58	-3.00	4.6E-04
R07C3.4	-1.58	-3.00	2.9E-04
C42D8.5b.1	-1.59	-3.00	1.4E-03
D2062.4	-1.59	-3.01	2.1E-04
ZK867.1c	-1.59	-3.01	6.8E-04
C33F10.8	-1.59	-3.01	2.1E-04
M04G7.2	-1.59	-3.01	4.5E-04
F57C7.5	-1.59	-3.01	3.4E-04
Y39F10C.1	-1.59	-3.02	2.1E-04
Y105C5B.11	-1.59	-3.02	5.0E-04
F55B11.1	-1.59	-3.02	3.1E-04
F59A3.8	-1.60	-3.02	2.5E-04
ZK867.1a	-1.60	-3.03	4.6E-04
T11F8.4	-1.60	-3.03	2.2E-04
F35E12.7d	-1.60	-3.03	2.9E-04
K07H8.5	-1.60	-3.03	2.3E-04
F10F2.6	-1.60	-3.03	2.1E-04
F08B1.2	-1.60	-3.03	3.1E-04
Y34B4A.10.1	-1.60	-3.03	2.2E-04
ZC581.3	-1.60	-3.04	4.6E-04
F45D11.1.2	-1.61	-3.04	2.3E-04
C54D2.5b	-1.61	-3.05	5.1E-04
F11G11.9	-1.61	-3.05	3.7E-04
B0379.7.2	-1.61	-3.05	6.5E-04
E02H4.1	-1.61	-3.05	2.1E-04
Y38E10A.5	-1.61	-3.05	2.0E-04
Y102A11A.2b	-1.61	-3.06	2.1E-04
T21B4.9	-1.61	-3.06	1.9E-04
Y11D7A.14	-1.62	-3.06	5.4E-03
F54E2.1	-1.62	-3.07	3.4E-04
B0218.1a.1	-1.62	-3.07	2.1E-04
T06D4.4	-1.62	-3.07	2.6E-04
ZK154.1	-1.62	-3.08	2.5E-04
B0228.4c	-1.62	-3.08	2.9E-04
C30F8.3	-1.62	-3.08	2.1E-04
F55H12.1	-1.62	-3.08	2.0E-04

Supporting Table 2: Significant changes after 48h in H_2S			
gene	logFC (48hr)	fold-change	adj.P.Val (48hr)
C09E8.2b	-1.62	-3.08	6.3E-04
C05E11.4	-1.62	-3.08	1.9E-04
C02B10.6	-1.62	-3.08	1.9E-04
K09F6.3	-1.62	-3.08	2.1E-04
Y46G5A.29	-1.63	-3.09	4.5E-04
ZC101.2e	-1.63	-3.09	1.7E-04
F15C11.1.2	-1.63	-3.09	3.5E-04
R10D12.1	-1.63	-3.10	2.9E-04
F18A12.6	-1.63	-3.10	3.0E-04
Y69E1A.8	-1.63	-3.10	1.8E-04
T05B4.12	-1.63	-3.10	4.5E-04
T23E7.2a	-1.63	-3.11	3.6E-04
ZC477.9a	-1.64	-3.11	2.3E-04
Y9C9A.3	-1.64	-3.11	1.8E-04
F54A5.2	-1.64	-3.11	3.1E-04
F15A2.6	-1.64	-3.12	1.9E-04
Y105C5A.13a	-1.64	-3.12	1.4E-03
F52F10.2	-1.65	-3.13	3.4E-03
F35E2.9	-1.65	-3.13	1.8E-04
M02D8.4b.1	-1.65	-3.14	1.5E-04
T16G1.2	-1.65	-3.14	5.1E-04
T25B9.4	-1.65	-3.14	1.5E-04
Y34B4A.10.2	-1.65	-3.14	2.8E-04
B0228.4e	-1.65	-3.14	2.9E-04
F22E12.1	-1.65	-3.14	2.2E-04
C50F4.2	-1.65	-3.14	3.1E-04
R09A8.4	-1.66	-3.15	2.4E-04
C31H1.1	-1.66	-3.16	2.1E-04
Y54G2A.25a	-1.66	-3.16	1.5E-04
R10F2.4	-1.66	-3.16	2.5E-04
C09G9.4	-1.66	-3.17	1.7E-04
F41F3.3.1	-1.67	-3.17	1.8E-04
F01G10.9	-1.67	-3.17	1.5E-04
Y39B6A.31	-1.67	-3.18	1.3E-04
T06D8.1a	-1.67	-3.18	1.9E-04
W08D2.1	-1.67	-3.18	1.4E-04
T06D8.1b	-1.67	-3.19	1.3E-04
Y73B6BL.34	-1.68	-3.20	2.5E-04
Y102A11A.2a	-1.68	-3.20	1.6E-04
Y106G6D.3	-1.68	-3.20	1.5E-04
C18E9.8	-1.68	-3.20	1.5E-04
C18H2.4	-1.68	-3.20	5.4E-04
Y71F9B.15	-1.68	-3.20	2.9E-04
T07H6.3b.2	-1.69	-3.22	2.6E-04
F35E2.6	-1.69	-3.22	2.0E-04
ZC513.3	-1.69	-3.22	1.6E-04
M162.7	-1.69	-3.22	3.2E-04

Supporting Table 2: Significant changes after 48h in H_2S			
gene	logFC (48hr)	fold-change	adj.P.Val (48hr)
C38D9.5	-1.69	-3.23	3.5E-04
Y94H6A.10	-1.69	-3.23	2.6E-04
F10G7.6	-1.69	-3.23	1.3E-04
F49C12.15	-1.69	-3.23	3.6E-04
C06G1.1	-1.69	-3.24	3.6E-04
F09F7.8	-1.70	-3.24	1.6E-04
F15B9.8	-1.70	-3.24	2.1E-04
Y39G8C.2	-1.70	-3.24	1.3E-04
T10E9.4.1	-1.70	-3.24	1.3E-04
F45D11.1.1	-1.70	-3.24	1.2E-04
Y48G1C.5	-1.70	-3.25	1.3E-04
T20D4.7	-1.70	-3.25	2.6E-04
B0393.4	-1.70	-3.26	2.1E-04
F58A3.2b.3	-1.70	-3.26	1.5E-04
F41A4.1	-1.70	-3.26	4.0E-04
T02C5.5d.1	-1.70	-3.26	1.7E-04
H12I19.4	-1.71	-3.27	1.3E-04
Y69E1A.3	-1.71	-3.27	1.1E-04
M01G12.9	-1.71	-3.27	1.5E-04
T07H3.1	-1.71	-3.28	3.6E-04
ZC434.9a	-1.71	-3.28	1.5E-04
K02E11.1	-1.71	-3.28	2.0E-04
M110.7	-1.71	-3.28	1.9E-04
K09F6.4	-1.72	-3.28	6.0E-04
T22C1.8	-1.72	-3.29	1.3E-04
Y39F10A.3	-1.72	-3.29	5.1E-04
Y18D10A.12	-1.72	-3.30	1.2E-04
C14F5.3c.2	-1.72	-3.30	1.4E-04
T20D4.15	-1.72	-3.30	2.5E-04
Y42H9B.1	-1.73	-3.31	2.2E-04
Y18H1A.15	-1.73	-3.31	1.6E-04
T08B6.9	-1.73	-3.31	3.1E-04
F02E11.1	-1.73	-3.32	1.2E-04
F26H9.8	-1.73	-3.32	1.1E-03
R01E6.5	-1.73	-3.32	1.0E-04
C16C8.2	-1.73	-3.32	2.1E-04
F22E10.3.2	-1.73	-3.32	1.0E-04
AH10.1	-1.73	-3.32	1.8E-04
K08C7.3a	-1.73	-3.33	1.1E-04
F35D11.2a.1	-1.74	-3.33	1.1E-04
Y4C6B.2b	-1.74	-3.33	1.0E-04
T05A7.6	-1.74	-3.34	1.2E-04
Y57G11A.2	-1.74	-3.34	3.7E-04
T01G5.2	-1.74	-3.34	1.0E-04
T06D4.3	-1.74	-3.35	9.5E-05
K12F2.1	-1.75	-3.35	1.3E-04
F39E9.4	-1.75	-3.35	2.1E-04

Supporting Table 2: Significant changes after 48h in H_2S			
gene	logFC (48hr)	fold-change	adj.P.Val (48hr)
F38A3.2	-1.75	-3.36	1.0E-04
Y48G8AL.12	-1.75	-3.36	2.7E-04
C08E3.10a	-1.75	-3.36	7.2E-04
C11H1.5	-1.75	-3.36	5.2E-04
Y71G12B.7	-1.75	-3.36	2.0E-04
T07A5.1	-1.75	-3.37	2.5E-04
Y70D2A.1	-1.75	-3.37	2.4E-04
D1054.9e	-1.75	-3.37	2.0E-04
F26A1.4	-1.76	-3.38	1.5E-04
C50H2.10	-1.76	-3.38	9.7E-05
ZK829.10	-1.76	-3.38	3.0E-04
H04M03.1	-1.76	-3.38	1.9E-04
R10D12.10	-1.76	-3.38	1.1E-04
Y80D3A.8	-1.77	-3.40	8.7E-05
C34C6.3	-1.77	-3.41	1.5E-04
D2092.8	-1.77	-3.41	1.5E-04
C48E7.8	-1.77	-3.42	8.2E-05
ZC247.1	-1.77	-3.42	1.5E-04
F15C11.1.1	-1.77	-3.42	1.5E-04
F10F2.5	-1.78	-3.43	1.6E-04
W01B11.5	-1.78	-3.44	1.5E-04
R155.2	-1.78	-3.44	1.0E-04
H05L14.1	-1.78	-3.45	9.6E-05
C09D1.1c	-1.79	-3.45	9.5E-05
Y43F8A.2	-1.79	-3.45	7.5E-05
C24D10.1	-1.79	-3.45	9.2E-05
F49E10.2a	-1.79	-3.46	1.4E-03
Y116A8C.4.1	-1.79	-3.46	9.5E-05
F21H7.2.1	-1.79	-3.46	8.9E-05
C26F1.1b	-1.79	-3.46	1.0E-04
Y54G2A.13	-1.79	-3.46	1.0E-04
B0491.2.2	-1.80	-3.47	1.2E-04
Y51A2B.6	-1.80	-3.47	2.0E-04
F23B12.3.2	-1.80	-3.48	1.1E-04
F39D8.1b	-1.80	-3.48	2.2E-03
F10F2.8	-1.80	-3.48	8.4E-05
ZC101.3	-1.80	-3.48	8.8E-05
T08G3.7	-1.80	-3.48	1.1E-04
ZK1290.3b	-1.81	-3.49	3.4E-04
Y48B6A.5	-1.81	-3.50	9.4E-05
F59A6.4	-1.81	-3.50	1.1E-04
T10H10.1	-1.81	-3.50	1.9E-04
T20D4.16	-1.81	-3.51	8.9E-05
Y22D7AR.7	-1.81	-3.51	2.5E-04
F38B6.5b.2	-1.81	-3.52	1.1E-04
F57C12.1	-1.82	-3.52	1.6E-04
Y116A8C.33	-1.82	-3.52	1.2E-04

Supporting Table 2: Significant changes after 48h in H_2S			
gene	logFC (48hr)	fold-change	adj.P.Val (48hr)
F10D11.6	-1.82	-3.52	6.0E-04
F36G9.13	-1.82	-3.53	1.2E-04
C17B7.12	-1.82	-3.53	1.8E-04
Y51A2B.1	-1.82	-3.53	3.0E-04
AC3.8	-1.82	-3.53	1.0E-04
T07H6.3b.1	-1.82	-3.54	3.4E-04
C01B12.1	-1.83	-3.55	1.2E-04
C45B2.7	-1.83	-3.56	5.6E-04
F09B12.1b.1	-1.83	-3.57	3.8E-04
B0205.13	-1.84	-3.57	3.4E-04
R09E10.3	-1.84	-3.58	1.3E-04
C14F5.3b	-1.84	-3.58	1.2E-04
T22C1.7	-1.84	-3.59	7.3E-05
F42C5.10	-1.84	-3.59	1.0E-04
F59A1.15	-1.84	-3.59	5.5E-05
Y25C1A.1	-1.84	-3.59	1.0E-04
T10B5.2	-1.84	-3.59	1.1E-04
F46C8.8	-1.85	-3.60	2.7E-04
C18H2.3	-1.85	-3.60	1.7E-03
R06B10.1	-1.85	-3.60	5.6E-05
F26C11.1	-1.85	-3.60	7.0E-05
F01D4.3	-1.85	-3.60	6.2E-05
B0350.2f.1	-1.85	-3.62	8.1E-05
Y54E2A.9	-1.86	-3.62	7.3E-05
K02B12.1	-1.86	-3.63	8.5E-05
H20J04.1	-1.86	-3.63	3.1E-04
K08B4.3	-1.86	-3.63	7.4E-05
F44G3.7	-1.86	-3.64	9.6E-05
C08F1.5b	-1.86	-3.64	8.3E-05
T19C3.2	-1.86	-3.64	6.2E-05
F13A7.7	-1.86	-3.64	9.5E-05
C10G8.5b	-1.86	-3.64	1.6E-04
T01D3.1	-1.87	-3.66	6.1E-05
F36D3.5	-1.88	-3.67	7.7E-05
M05B5.3	-1.88	-3.67	5.2E-05
F48F5.1	-1.88	-3.68	6.0E-05
W09C3.2	-1.88	-3.68	5.4E-05
R09B5.5	-1.88	-3.69	3.4E-04
F55G11.5	-1.89	-3.69	1.0E-04
F46F5.6	-1.89	-3.70	1.2E-04
K07E12.1a	-1.89	-3.70	6.6E-05
ZK616.7	-1.89	-3.70	6.5E-05
K06A9.1a	-1.89	-3.70	1.0E-04
Y47G6A.15b	-1.89	-3.71	4.4E-05
W06H8.8e	-1.89	-3.71	4.9E-05
K10D11.2	-1.89	-3.71	9.2E-05
F22E10.2	-1.89	-3.72	4.9E-05

Supporting Table 2: Significant changes after 48h in H_2S			
gene	logFC (48hr)	fold-change	adj.P.Val (48hr)
C06C6.6	-1.89	-3.72	5.4E-05
F14F9.4	-1.89	-3.72	9.4E-05
T01B7.7	-1.90	-3.72	8.6E-05
K09H11.4	-1.90	-3.73	4.3E-05
W03F9.11	-1.90	-3.73	1.9E-04
M02D8.4a	-1.90	-3.74	4.4E-05
F47F6.5	-1.90	-3.74	6.9E-05
T07H6.4	-1.90	-3.74	5.5E-05
F22E10.3.1	-1.91	-3.75	4.3E-05
T22H9.3	-1.91	-3.75	9.2E-05
C41H7.7	-1.91	-3.75	9.2E-05
T27E4.6	-1.91	-3.76	8.1E-05
Y95B8A.1	-1.91	-3.76	7.4E-04
Y53C10A.9	-1.91	-3.76	1.1E-04
W03B1.5	-1.92	-3.77	5.4E-05
F18A1.1	-1.92	-3.78	1.1E-04
C45G9.6a	-1.92	-3.79	6.5E-05
F01G10.3	-1.92	-3.79	6.6E-05
C49G7.5	-1.93	-3.80	1.6E-04
F54E2.3b	-1.93	-3.81	4.3E-05
K01D12.5	-1.93	-3.81	2.5E-03
Y5H2A.4	-1.93	-3.82	2.0E-03
Y71F9B.14	-1.94	-3.83	8.9E-05
ZK1010.5	-1.95	-3.86	7.8E-05
F47B8.11	-1.95	-3.86	1.9E-04
F01G10.5	-1.95	-3.86	9.2E-05
R10D12.17	-1.95	-3.87	4.8E-05
BE10.2	-1.96	-3.89	3.8E-04
F45E4.1	-1.96	-3.89	3.4E-04
Y43D4A.5	-1.96	-3.89	1.0E-04
Y69A2AR.14	-1.96	-3.90	3.9E-05
C01G10.1	-1.96	-3.90	7.0E-05
M70.1	-1.96	-3.90	3.8E-05
Y47D3A.11	-1.96	-3.90	4.0E-05
W06H8.8f	-1.97	-3.91	2.9E-05
M70.3b	-1.97	-3.91	4.9E-05
F32E10.3	-1.97	-3.92	1.4E-04
B0491.2.1	-1.97	-3.93	3.8E-05
T01D3.3a	-1.97	-3.93	1.1E-04
R11A5.7.1	-1.98	-3.95	7.7E-05
B0222.7	-1.98	-3.96	2.7E-05
Y49F6B.10	-1.99	-3.96	4.9E-05
Y67A10A.3	-1.99	-3.96	6.5E-05
Y41C4A.11	-1.99	-3.97	2.1E-04
K09C6.1	-1.99	-3.98	6.9E-05
B0350.2f.2	-1.99	-3.98	5.0E-05
C08G5.3	-1.99	-3.99	3.6E-05

Supporting Table 2: Significant changes after 48h in H_2S

gene	logFC (48hr)	fold-change	adj.P.Val (48hr)
C01G12.8	-2.00	-3.99	3.4E-05
Y46G5A.25	-2.00	-3.99	6.6E-05
Y38C1BA.3	-2.00	-4.00	8.0E-05
C01G12.10	-2.00	-4.00	3.6E-05
T24E12.5	-2.00	-4.01	2.9E-05
T23F2.1.1	-2.00	-4.01	1.9E-04
M02D8.4c	-2.01	-4.02	2.6E-05
F23B12.3.1	-2.01	-4.02	3.5E-05
C18H7.1.2	-2.01	-4.02	2.9E-05
Y54E10BL.2	-2.01	-4.02	1.1E-04
Y39B6A.24.1	-2.01	-4.02	2.6E-05
Y70C5C.5	-2.01	-4.03	2.5E-05
F18A12.3	-2.01	-4.03	2.5E-05
F23B2.12.1	-2.02	-4.04	2.4E-05
C42D8.5a	-2.02	-4.06	3.7E-04
C31E10.2	-2.02	-4.06	2.7E-05
C34F11.5	-2.02	-4.06	3.7E-05
C06G1.2	-2.03	-4.07	8.0E-05
T10E9.3	-2.03	-4.08	2.1E-04
Y39B6A.30	-2.03	-4.09	6.6E-05
T19D12.4a	-2.03	-4.09	2.4E-05
F18A12.1	-2.03	-4.09	3.1E-05
C17H12.6	-2.03	-4.09	5.6E-05
ZK757.3b	-2.03	-4.10	6.1E-05
R12E2.7	-2.04	-4.10	5.2E-05
H02K04.1	-2.04	-4.11	1.1E-04
W02D9.9	-2.04	-4.12	1.3E-04
R08F11.3	-2.04	-4.12	3.8E-05
K09C6.8	-2.04	-4.12	2.7E-05
F43C9.1	-2.05	-4.13	2.6E-05
C24F3.5	-2.05	-4.14	4.4E-05
K06A4.4	-2.06	-4.16	3.7E-05
C25F6.4	-2.06	-4.17	2.2E-05
T28A11.20	-2.06	-4.17	2.1E-05
Y69A2AR.19	-2.07	-4.19	2.1E-05
C46A5.3b	-2.07	-4.20	3.7E-05
K11D12.4	-2.07	-4.20	2.7E-05
C04E6.4	-2.07	-4.21	3.0E-05
F21C10.7	-2.07	-4.21	2.2E-05
T10E10.2.2	-2.08	-4.22	3.4E-05
F45E4.3a	-2.08	-4.22	3.5E-05
F22E10.1	-2.08	-4.22	1.8E-05
Y4C6B.2a	-2.08	-4.23	3.5E-05
F10F2.7	-2.08	-4.23	1.8E-05
Y39B6A.18	-2.10	-4.30	4.8E-05
F32A5.2b	-2.11	-4.31	1.6E-05
Y113G7C.1	-2.11	-4.31	1.7E-05

Supporting Table 2: Significant changes after 48h in H_2S			
gene	logFC (48hr)	fold-change	adj.P.Val (48hr)
F26D2.10	-2.11	-4.32	4.9E-05
F40E12.2	-2.11	-4.32	2.1E-05
E03G2.4	-2.11	-4.32	1.7E-05
F22D6.10	-2.11	-4.33	3.7E-05
Y69A2AR.8	-2.12	-4.34	1.9E-05
K04H4.2a.1	-2.12	-4.34	2.6E-03
H39E23.3	-2.12	-4.35	1.3E-04
W03F11.4	-2.13	-4.36	2.2E-05
T22B3.2b	-2.14	-4.39	4.0E-05
M117.4	-2.14	-4.40	2.5E-05
ZK783.6	-2.14	-4.40	2.4E-05
C05E4.9a.1	-2.14	-4.41	2.9E-05
C06C6.7	-2.14	-4.41	4.8E-05
T28C6.4	-2.15	-4.44	2.4E-05
C49G7.1	-2.16	-4.46	4.1E-05
F39B3.3	-2.16	-4.47	1.3E-05
F54D12.3	-2.18	-4.53	1.3E-05
ZK617.1a.1	-2.18	-4.54	1.1E-05
F54E2.3a	-2.19	-4.55	1.2E-05
R155.3	-2.19	-4.56	2.9E-05
F54E2.3d	-2.20	-4.58	1.2E-05
F22B3.7	-2.20	-4.59	1.2E-05
B0024.1	-2.20	-4.60	2.0E-05
F22B8.6.1	-2.20	-4.61	2.9E-05
F32G8.5	-2.21	-4.61	2.5E-05
F44E5.4.1	-2.21	-4.62	2.6E-04
K04H4.2a.3	-2.21	-4.63	1.4E-03
T20D4.10	-2.23	-4.68	1.2E-05
Y11D7A.11	-2.23	-4.70	5.3E-05
CD4.9	-2.24	-4.71	1.8E-04
M70.3a	-2.24	-4.72	3.0E-05
C34H4.1	-2.24	-4.73	3.1E-05
ZK377.1	-2.25	-4.74	4.8E-05
H04M03.2	-2.25	-4.76	2.9E-05
F37A4.4	-2.25	-4.77	8.1E-06
C46A5.3a	-2.26	-4.78	1.0E-04
F47F6.3	-2.27	-4.81	5.3E-05
C09G5.6	-2.28	-4.86	3.9E-04
H02F09.3	-2.28	-4.86	1.3E-05
C44C10.1	-2.29	-4.89	1.8E-05
F25G6.7a	-2.29	-4.90	2.0E-05
F25G6.7b	-2.29	-4.91	1.2E-05
F38B6.5b.1	-2.30	-4.93	1.2E-05
F25G6.6	-2.32	-5.01	6.2E-06
F30B5.1	-2.33	-5.03	2.7E-05
ZK84.1	-2.33	-5.03	9.3E-06
Y37H2A.11	-2.33	-5.03	4.6E-05

Supporting Table 2: Significant changes after 48h in H_2S			
gene	logFC (48hr)	fold-change	adj.P.Val (48hr)
T19D2.1	-2.34	-5.06	4.9E-06
T10E10.2.3	-2.34	-5.06	6.1E-06
Y22D7AL.14	-2.34	-5.06	5.4E-06
T19A5.3a	-2.34	-5.06	6.5E-05
K11H12.4	-2.34	-5.07	1.1E-05
Y22D7AR.12	-2.35	-5.10	5.6E-06
ZK678.5.2	-2.37	-5.15	3.8E-05
C29E4.1	-2.37	-5.16	6.7E-06
B0024.4	-2.39	-5.25	5.1E-06
Y69A2AR.9	-2.40	-5.29	4.9E-06
F56H1.3	-2.41	-5.31	6.6E-05
T01D3.3b	-2.41	-5.32	7.0E-06
F27C1.8.1	-2.41	-5.33	3.6E-06
F15G9.4a	-2.42	-5.35	3.9E-06
T10E10.1	-2.42	-5.36	6.7E-06
C08F11.14	-2.44	-5.44	2.2E-05
C38C3.7	-2.46	-5.52	9.2E-06
T10E10.2.1	-2.49	-5.63	9.4E-06
F54F12.1	-2.50	-5.64	2.4E-06
F56D3.1.1	-2.51	-5.68	6.0E-06
C18H7.1.1	-2.51	-5.68	1.6E-05
ZK757.3a.1	-2.51	-5.69	8.3E-06
T19D12.4b	-2.51	-5.70	3.1E-06
C29F4.1.1	-2.51	-5.70	4.8E-06
T10H4.11	-2.51	-5.70	1.3E-05
F54E2.3c	-2.52	-5.74	2.6E-06
Y53F4B.27a.1	-2.53	-5.77	2.6E-05
C01G5.4	-2.54	-5.84	2.2E-06
F58F6.1	-2.58	-5.96	4.5E-06
C18H7.11	-2.58	-5.97	4.2E-06
H06I04.5	-2.59	-6.03	3.9E-06
C09B9.7	-2.59	-6.04	7.9E-06
F56D3.1.2	-2.60	-6.05	1.9E-06
F27C1.8.2	-2.60	-6.06	1.8E-06
H43E16.1	-2.62	-6.14	2.0E-06
B0207.1	-2.62	-6.15	5.1E-06
T10E10.5	-2.63	-6.21	3.9E-06
E01G4.6	-2.64	-6.21	7.0E-05
F22B8.5	-2.64	-6.23	3.2E-06
F36G9.12	-2.66	-6.33	1.5E-06
R03C1.1	-2.68	-6.42	8.6E-03
M195.2	-2.70	-6.50	4.2E-05
T19A5.3b	-2.72	-6.57	3.1E-04
Y51A2D.4.1	-2.72	-6.59	4.4E-06
H10E21.4	-2.72	-6.61	2.7E-06
F15G9.4b	-2.73	-6.63	1.4E-06
C38C6.6.1	-2.74	-6.67	1.9E-06

Supporting Table 2: Significant changes after 48h in H_2S			
gene	logFC (48hr)	fold-change	adj.P.Val (48hr)
H23L24.5	-2.75	-6.72	9.1E-07
Y116F11B.3.1	-2.76	-6.79	7.5E-07
T20D4.4	-2.79	-6.93	7.6E-07
C38C6.6.2	-2.80	-6.97	8.3E-07
ZC168.5	-2.80	-6.97	2.6E-06
C07G3.2	-2.81	-6.99	9.9E-06
T21E8.2	-2.83	-7.10	1.1E-05
K09H9.3	-2.83	-7.10	6.5E-07
T06E4.4	-2.83	-7.10	4.5E-06
C43C3.1	-2.83	-7.12	6.2E-07
T05A10.5	-2.84	-7.14	1.2E-06
Y65B4BR.6a	-2.85	-7.22	6.0E-07
F52D1.3	-2.87	-7.32	1.8E-05
T01D1.6	-2.87	-7.33	1.7E-04
Y41E3.2	-2.88	-7.35	6.8E-07
C09G5.4	-2.89	-7.43	2.2E-06
C52D10.13	-2.89	-7.44	7.6E-07
F15H10.1.1	-2.89	-7.44	1.0E-06
T05A10.4	-2.90	-7.46	6.0E-07
F41E6.11	-2.90	-7.48	5.5E-04
M195.1	-2.92	-7.57	6.0E-07
F11G11.10	-2.93	-7.61	6.5E-07
F08G5.4	-3.03	-8.15	2.9E-07
F11G11.12	-3.10	-8.58	1.1E-06
C49G7.7	-3.13	-8.78	1.3E-06
C03A7.8	-3.16	-8.92	1.5E-04
C42D4.3	-3.18	-9.07	8.8E-07
C03A7.7	-3.18	-9.08	3.1E-04
C50B6.4	-3.22	-9.34	5.1E-07
W05G11.3.1	-3.22	-9.35	7.5E-07
K02E11.10	-3.23	-9.36	5.7E-07
ZK180.5c	-3.24	-9.42	2.6E-07
F09G8.6	-3.25	-9.52	1.9E-07
F35B3.4	-3.28	-9.69	4.9E-07
T06E4.11	-3.28	-9.69	8.0E-06
ZK1067.7	-3.33	-10.05	5.2E-05
C29F3.2	-3.35	-10.18	4.8E-07
Y65B4BR.6b	-3.35	-10.21	3.2E-07
C03A7.4	-3.35	-10.22	3.4E-04
Y57A10A.11	-3.36	-10.25	2.9E-07
T10E10.6	-3.37	-10.35	1.7E-07
C16E9.1	-3.38	-10.44	9.5E-08
ZK180.6	-3.45	-10.90	1.4E-06
ZK180.5b.1	-3.47	-11.10	2.9E-07
Y53F4B.27b.1	-3.48	-11.14	1.2E-06
F54D1.3	-3.50	-11.35	2.9E-07
F57B7.3	-3.53	-11.57	6.8E-08

Supporting Table 2: Significant changes after 48h in H_2S

gene	logFC (48hr)	fold-change	adj.P.Val (48hr)
T20D4.5	-3.61	-12.25	1.1E-07
C05A9.1	-3.71	-13.08	3.9E-08
ZK180.5a	-3.75	-13.43	4.5E-08
T21E8.1b	-3.79	-13.79	2.9E-08
F54D1.2	-3.87	-14.62	1.2E-07
T06E4.6	-4.11	-17.25	4.2E-07
Y47D7A.15	-4.27	-19.31	6.8E-09
Y47D7A.13.1	-4.27	-19.33	6.8E-09
C15A11.5	-4.33	-20.11	1.2E-07
T21E8.1a	-4.33	-20.17	6.8E-09
T20D4.3	-4.34	-20.27	1.1E-08
T18H9.1	-4.35	-20.45	8.0E-09
F44G3.2	-4.68	-25.67	4.2E-08
