# Supplementary material for: HIF-1 and SKN-1 Coordinate the Transcriptional Response to Hydrogen Sulfide in Caenorhabditis elegans
Source: PLoS One. 2011 Sep 29;6(9):e25476. doi: 10.1371/journal.pone.0025476 (PMC3183046; doi:10.1371/journal.pone.0025476)
Supplement: Table S3 — Genes included in functional annotation clusters. (PDF) [file pone.0025476.s003.pdf]

Supporting Table 3: Functional Annotation Clusters

**12h H_2S Exposure**	
Aging and Stress Response cluster	
hsp-16.48	*ins-7*
hsp-16.2	*mtl-1*
	pes-2.1
F-box associated cluster	
fbxb-13	*sdz-11*
F22E5.20	Y27F2A.8
	pes-2.1
**48h H_2S exposure**	
F-box associated cluster	
C38D9.8	*fbxb-96*
fbxb-22	*fbxb-75*
fbxb-57	*fbxb-103*
fbxb-21	*fbxb-48*
fbxb-43	*fbxb-90*
fbxb-66	*fbxb-20*
fbxb-42	*fbxb-97*
fbxb-95	F22E5.20
fbxb-44	*pes-2.1*
fbxb-101	*sdz-33*
fbxb-41	*sdz-9*
fbxb-13	*sdz-5*
Y27F2A.8	*sdz-16*
Y44A6C.2	*sdz-25*
	sdz-11
BTB/POZ associated cluster	
btb-8	*sdz-28*
btb-11	*skr-9*
	skr-10
transcription cluster	
tbx-43	*sepa-1*
Y51H4A.4	*tbx-38*
F23F12.9	*hnd-1*
	pes-10
metal binding cluster	
Y60A9.3	*ccch-2*
nas-3	Y82E9BR.17
Y73C8C.8	*mtl-1*
	Y116A8C.19
