# Supplementary material for: HIF-1 and SKN-1 Coordinate the Transcriptional Response to Hydrogen Sulfide in Caenorhabditis elegans
Source: PLoS One. 2011 Sep 29;6(9):e25476. doi: 10.1371/journal.pone.0025476 (PMC3183046; doi:10.1371/journal.pone.0025476)
Supplement: Table S4 — Transcripts altered by both 1 h exposure to H2S and hypoxia. (PDF) [file pone.0025476.s004.pdf]

Supporting Table 4: Genes regulated by both H_2S and hypoxia

**genes regulated by H_2S (1h) and hypoxia (*hif-1*-dependent)**	
cysl-2	
nhr-57	
rhy-1	
**genes regulated by H_2S (12h) and hypoxia (*hif-1*-dependent)**	
increased in H_2S	decreased in H_2S
cyp-36A1	F45D11.1.1
cysl-2	*ketn-1*
rhy-1	R10D12.1
**genes regulated by H_2S (12h) and hypoxia (all)**	
increased in H_2S	decreased in H_2S
clec-222	*amx-2*
cyp-36A1	F10D11.6
F19B10.2	K10G6.4
cysl-2	R10D12.1
M05D6.6	*grd-6*
M162.5	Y57G11C.21
rbx-2	
srv-7	

Overlap shown in Figure 2B of main text
hypoxia-regulated genes from Shen et al (2005)
