# Supplementary material for: HIF-1 and SKN-1 Coordinate the Transcriptional Response to Hydrogen Sulfide in Caenorhabditis elegans
Source: PLoS One. 2011 Sep 29;6(9):e25476. doi: 10.1371/journal.pone.0025476 (PMC3183046; doi:10.1371/journal.pone.0025476)
Supplement: Table S5 — Transcripts regulated by skn-1 in other studies that are altered by exposure to H2S. (PDF) [file pone.0025476.s005.pdf]

Supporting Table 5: Genes changed in H_2S and regulated by *skn-1*	
**upregulated by *skn-1* in unstressed conditions***	
increased in H_2S	decreased in H_2S
C31C9.2	*kqt-2*
nspa-9	*pcp-2*
clec-17	*ifp-1*
F01D5.3	*amt-1*
F01D5.5	
gst-4	
cysl-2	
dhs-23	
nspa-8	
rhy-1	
nspa-5	
nit-1	
**down-regulated by *skn-1* in unstressed conditions***	
increased in H_2S	decreased in H_2S
ilys-2	Y39B6A.24
Y47H9C.1	
ins-7	
**upregulated by arsenic stress***	
increased in H_2S	decreased in H_2S
nspa-9	*pcp-2*
F01D5.3	
gst-4	
cysl-2	
M05D6.6	
dhs-23	
nspa-8	
nspa-5	
nit-1	
**upregulated by t-butyl hydroperoxide***	
increased in H_2S	decreased in H_2S
C31C9.2	*amt-1*
gst-4	C01G5.4
nit-1	
**regulated in hyperoxia**[#]	
increased in H_2S	
gst-16	
gst-4	
hsp-16.41[+]	
nsp-16.2[+]	

Overlapping genes referred to in Figure 3B of main text
**skn-1*-dependent transcripts from Oliveira et al (2009)
[#]*skn-1*-dependent transcripts from Park et al (2009)
[+]downregulated in hyperoxia
